# Structural dynamics of substrate processing by the p97-Ufd1-Npl4-FAF1 machinery

**DOI:** 10.1038/s41467-026-78639-x

**Published:** 2026-10-25

**Authors:** Athina Drakonaki, Johannes van den Boom, Alexander Kröning, Hemmo Meyer, Christos Gatsogiannis

**Affiliations:** 1 Institute of Medical Physics and Biophysics, University of Münster, Münster, Germany; 2 Center for Soft Nanoscience (SoN), University of Münster, Münster, Germany; 3 Molecular Biology I, Center of Medical Biotechnology, Faculty of Biology, University of Duisburg-Essen, Essen, Germany

**Keywords:** Cryoelectron microscopy, Mechanism of action, Molecular conformation, Ubiquitylated proteins

## Abstract

The AAA-ATPase VCP/p97 unfolds ubiquitylated proteins in key signaling and cellular homeostasis pathways, yet the mechanisms by which Ufd1-Npl4 and FAF1 adapters handle ubiquitin chains to coordinate substrate loading and processing remain unclear. Our cryo-EM study of p97-Ufd1-Npl4 and FAF1 engaging a substrate reveal interactions that drive substrate processing. While Npl4 is capped by distal ubiquitins, and the initiator ubiquitin can be traced from the Npl4 groove to the ATPase channel at near-atomic resolution, we find that the Npl4 tower adaptively responds to the configurational changes in the ATPase motor’s spiral staircase. A helix in FAF1 together with the Ufd1-UT3 domain engage proximal ubiquitins while providing the necessary structural plasticity to accommodate this motion and deliver ubiquitins to Npl4’s groove. Our study reveals the intricate, dynamic interplay between p97, Ufd1-Npl4, and FAF1 in efficient processing of ubiquitylated substrates.

## Abstract (web summary)

VCP/p97 uses Ufd1-Npl4 and FAF1 to process polyubiquitylated substrates. Here, the authors show how these adapters coordinate ubiquitin-chain engagement and delivery while adapting to conformational changes in the p97 ATPase motor during substrate processing.

## Introduction

p97, also known as valosin- containing protein (VCP) in mammals and Cdc48 in yeast, is a conserved AAA+ ATPase (ATPases Associated with various cellular Activities) that plays a central role in maintaining cellular homeostasis^1–3^. By using ATP hydrolysis, p97 drives conformational changes that remodel or unfold substrate proteins marked with polyubiquitin chains^4^. This activity is essential for a plethora of cellular pathways ranging from clearance of damaged proteins and organelles to key signaling events, which include autophagy, mitochondrial-protein degradation, DNA damage repair, and endoplasmic reticulum-associated degradation (ERAD)^1,2,5^. Consistent with its importance, point mutations in p97 are associated with a degenerative disease, multisystem proteinopathy-1, with features such as inclusion body myopathy, frontotemporal dementia, and familial amyotrophic lateral sclerosis^6,7^. Additionally, p97 is up-regulated in certain types of cancer, thus making it a desirable candidate for drug development^3^.

p97 is a type II AAA+ that forms a hexameric ring with each protomer consisting of an N-terminal domain (NTD), and two ATPase domains (D1 and D2)^4^. Cryo-EM studies have shown that Cdc48/p97 assumes a spiral staircase conformation, allowing aromatic residues of central pore loops to interact with the backbone of the unfolded substrate. Substrate translocation is proposed to occur through a processive, hand-over-hand mechanism^8–13^, where ATP hydrolysis and subsequent ATP binding to the lowest protomer drive its movement to the top of the hexamer, a mechanism likely conserved among all AAA+ unfoldases^14–18^. Thereby, the peptide is pulled through the pore as nucleotide binding and hydrolysis progress around the ring, with a step size of two residues per hydrolyzed ATP.

p97 cooperates with a host of substrate adapters, accessory adapters, and additional modulators^19–21^. The primary adapter for ubiquitylated substrates is the Ufd1-Npl4 (UN) heterodimer, which binds p97 through a UBXL domain in Npl4 and a SHP box in Ufd1 that interact with the NTDs on the substrate-entry side of p97 (cis-side), positioning Npl4 as a tower above p97’s D1 pore, as previously described for the homologous Cdc48 system^10,22^. Studies of the yeast Cdc48-UN complex revealed that UN binds and unfolds an initiator ubiquitin within the ubiquitin chain, and inserts it into the p97 D1 pore for initiation of substrate threading^10,20,23,24^. Initiator unfolding is triggered by binding to a hydrophobic groove on Npl4, which leads to the p97 pore. Notably, Npl4 assumes different conformations through alternating interfaces with the D1 ring and N-termini of the ATPase^25^, whose functional implications remain unclear, including how these conformations are coordinated with the sequential movements of individual subunits of p97 during the ATPase cycle. Structural studies of p97/Cdc48 have shown two to three distal ubiquitin molecules capping Npl4^10,26,27^. Nevertheless, the arrangement of the remaining ubiquitin chain elements and the ubiquitin-binding UT3 domain of Ufd1, as well as the mechanism ensuring processivity during substrate threading beyond initiator insertion, is unclear.

Moreover, mammalian p97-UN was recently shown to require accessory adapters, such as FAF1, for efficient handling and unfolding of ubiquitylated substrates^28,29^. FAF1 assists p97 in a variety of important pathways, including regulation of DNA replication^30–32^. FAF1 comprises a ubiquitin-binding UBA domain, a thioredoxin-fold UAS domain that is linked via a helix to the UBX domain that generally serves to dock cofactors to the NTDs of p97. Although structure predictions indicate that FAF1 interacts with the Ufd1 UT3 domain and facilitates ubiquitin chain binding^28^, high-resolution structural analysis is needed to elucidate how FAF1 orchestrates ubiquitin chain interaction with respect to the substrate. Moreover, it is unclear how this is coordinated with the dynamics of required Npl4 movements relative to the p97 spiral movements for efficient substrate insertion and processive threading.

In this study, we investigate the interaction between p97 and its adapter proteins Ufd1-Npl4 and the C-terminal domain of FAF1 in the presence of a polyubiquitinated substrate. We achieve near-atomic resolution of the unfolded initiator ubiquitin, enabling unambiguous model building and revealing extensive interactions with both p97 and Npl4. A single FAF1 molecule bridges the complex by docking its UBX domain onto a specific NTD of p97, while an extended helix of FAF1 engages the UT3 domain of Ufd1 and two proximal ubiquitin moieties. Concurrently, binding of the UBX domain to the NTD of p97 stabilizes the SHP motif of Ufd1. We resolve four distinct states of the FAF1-UN-p97 complex, which show that Npl4 adopts different conformations depending on the position of the seam subunit of the ATPase. FAF1 remains bound to the same NTD of p97, with the extended helix providing structural plasticity via a tilt-engage mechanism to accommodate the rearrangements of p97/Npl4 and positioning of the substrate along the spiral staircase. Combining these findings with functional data and AlphaFold predictions, our study reveals the orchestrated interplay between FAF1, p97, and UN during substrate processing, providing critical insights into the molecular mechanisms underlying protein quality control and degradation.

## Results

### The overall architecture of substrate-engaged p97 in complex with Ufd1-Npl4 and C-terminal domain of FAF1

To obtain structural insights into the mechanism by which FAF1 co-operates with UN to mediate substrate engagement and facilitate substrate threading, we reconstituted the p97- Ufd1-Npl4 in complex with different truncations of FAF1 in vitro (Supplementary Table 1, Supplementary Fig. 1, Supplementary Fig. 2).

The complexes were then incubated in the presence of ADP and beryllium fluoride (ADP·BeF(x)) to induce substrate engagement and lock the ATPase into an early pre-hydrolysis state. To enhance complex stability, we introduced two mutations, A232E and E578Q, into p97, as previously described^23^. These mutations reduced the unfolding activity of p97 while maintaining its functionality and minimizing conformational heterogeneity of the NTDs. All structural work in this study was conducted using p97 (A232E, E578Q), Ufd1-Npl4 (UN), and biochemically confirmed K48-polyubiquitinated Ub-Eos (Ub_n_-Eos), as a substrate (Supplementary Fig. 1b).

**Fig. 1:**
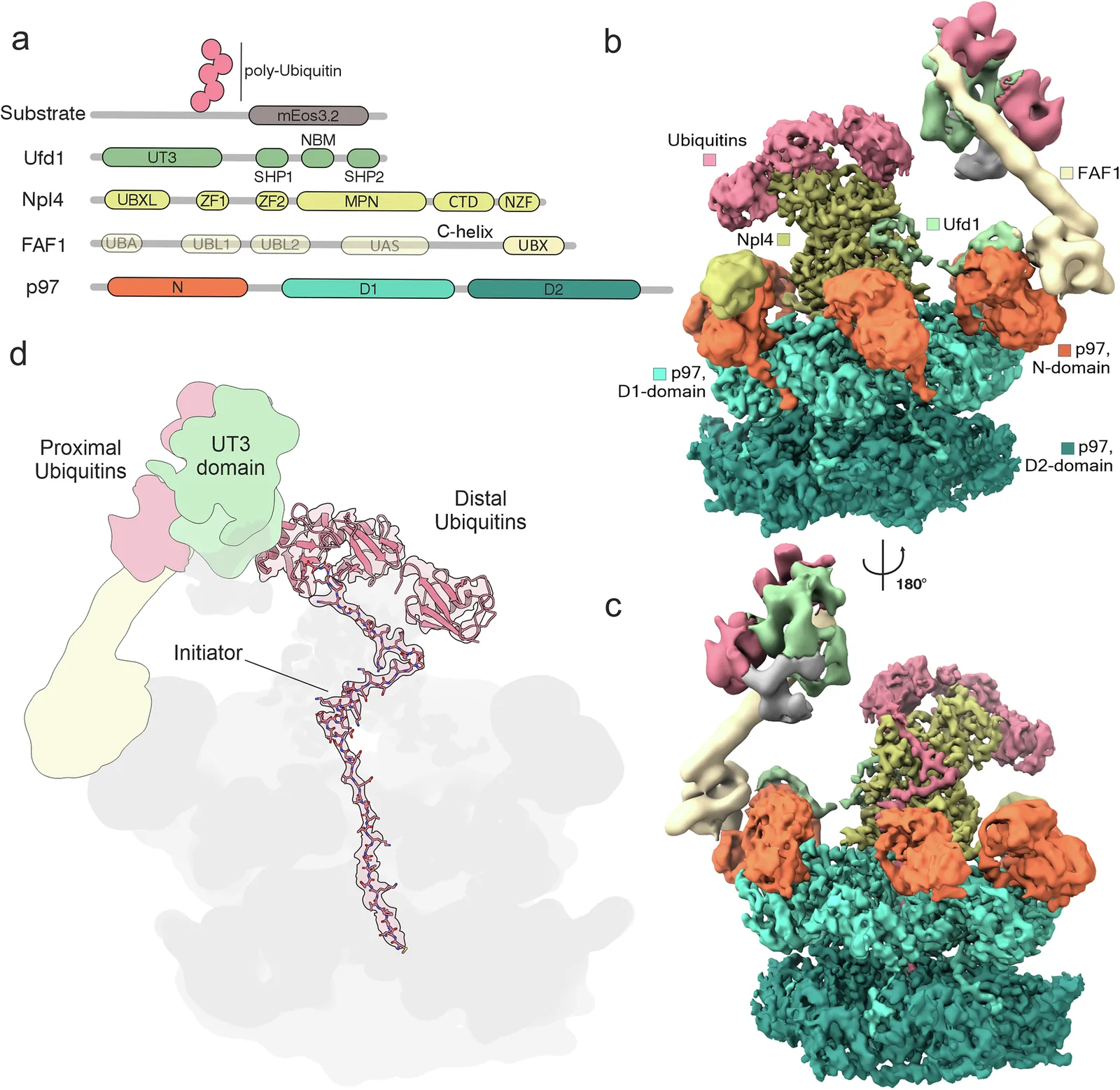
Structure of substrate engaged p97 in complex with Npl4/Ufd1 and C terminal domain of FAF1. **a** Schematic representation of p97 (D2 dark cyan, D1 cyan, NTDs orange), Npl4 (dark khaki), Ufd1 (green), only-C-term FAF1 (yellow) and poly-ubiquitinated substrate (pink) domain organization. This coloring scheme is maintained throughout the paper. **b** Composition map of cryo-EM structure of the substrate-engaged p97 in complex with Ufd1-Npl4 and the C terminal domain of FAF1 as viewed from the front and from the back (**c**). **d** Detailed view of the unfolded initiator ubiquitin along with distal and proximal to the substrate ubiquitins. The State II consensus map (EMD-58590) shows p97, whereas locally refined maps highlight the UN tower (EMD-58591) and the FAF1-UT3-proximal ubiquitin region (EMD-58592). Density is contoured at 15.7σ for FAF1-UT3-proximal ubiquitin region, 6.2σ for UN tower, and 7.8σ for p97. The p97, UN tower and FAF1 components are contoured independently and their apparent occupancy or relative density strength should therefore not be compared directly.

To investigate the dynamic interactions between p97, UN, and FAF1, we used cryoEM to screen p97/UN/Ub_n_-Eos complexes assembled with the assorted truncated FAF1 variants, generating small datasets for each. Despite locking the complex in the initial state of substrate processing, our analyses revealed significant heterogeneity on the cis-side of the ATPase, indicating a highly dynamic complex with multiple conformational states among all components (Supplementary Fig. 3). To tackle this challenge and elucidate the structural dynamics of the p97-UN-FAF1 interplay during substrate processing, we focused on the complex with the Δ_1-480_ FAF1 variant (C-terminal FAF1), which encompasses the C-terminal UBX domain that binds to the NTD of p97 followed by the first 65 residues of an extended α-helix, while omitting the N-terminal region (UAS, UBL1, UBL2, and UBA domains, Fig. 1a and Supplementary Fig. 2). Truncated variants of FAF1, comprising only the C-terminal helix and the UBX domain, preserve their ability to enhance the efficiency of p97-UN in substrate unfolding^28^. Thus, the use of Δ_1-480_ FAF1 (C-terminal FAF1) improved complex stability and decreased heterogeneity, while the complex remained functional. Nevertheless, analysis of a preliminary cryoEM dataset (20,000 movies) revealed that 34% of the particles included all components, while the cis-side of the complex (UN/FAF1/substrate region) still exhibited significant heterogeneity (Supplementary Fig. 3c).

To ensure a sufficient number of particles for an extensive heterogeneity analysis of the complex’s dynamics, we collected and processed approximately 280,000 cryoEM movies. This large-scale dataset was necessary to account for the inherent variability of the system. The data collection spanned ~12.5 days in a Titan KRIOS G4 cryoTEM equipped with a Falcon 4i direct detector.

Following extensive classification of the dataset (Supplementary Fig. 4), we identified multiple states of the complex, which will be detailed in subsequent sections. For clarity, we begin by describing the overall architecture of the complex based on our highest-resolution cryoEM structure (State II), which achieved resolutions of 3.1 Å for p97 and Ufd1-Npl4, and an average resolution of 8 Å for the C-terminal domain of FAF1, in complex with the Ub_n_-Eos model substrate (Fig. 1b, c and Supplementary Fig. 5b).

Npl4 is anchored to the D1 domain of p97 in a slightly tilted configuration with respect to the p97 D1 pore axis. Notably, we observe continuous density for the initiator Ub, stretching from the groove of Npl4 down to the D2 ring of p97 (Fig. 1b–d). p97 is thereby arranged in the characteristic staircase conformation as observed in previous studies^9,10,12^. Ufd1 is tightly associated with Npl4 via its NBM region. We further observe three Ub moieties crowning the Ufd1-Npl4 tower.

In contrast to previous low-resolution studies suggesting three FAF1 molecules bind to p97^33^, our structure reveals a single FAF1 copy per p97 hexamer, as confirmed by mass photometry and functional unfolding assays (Supplementary Fig. 6). Specifically, the UBX domain of FAF1 binds exclusively to the NTD of p97 positioned opposite to Npl4, located four subunits clockwise from the NTD that engages with the UBXL domain of Npl4. The helical region of FAF1 points to the distal Ubs capping the UN tower. Notably, the upper segment of the helix makes contact with two previously unobserved Ubs proximal to the substrate and also the UT3 domain of Ufd1, as detailed below, highlighting a role in substrate recognition and processing. The following sections provide a detailed description of the interactions.

#### The initiator Ubiquitin

**Fig. 2:**
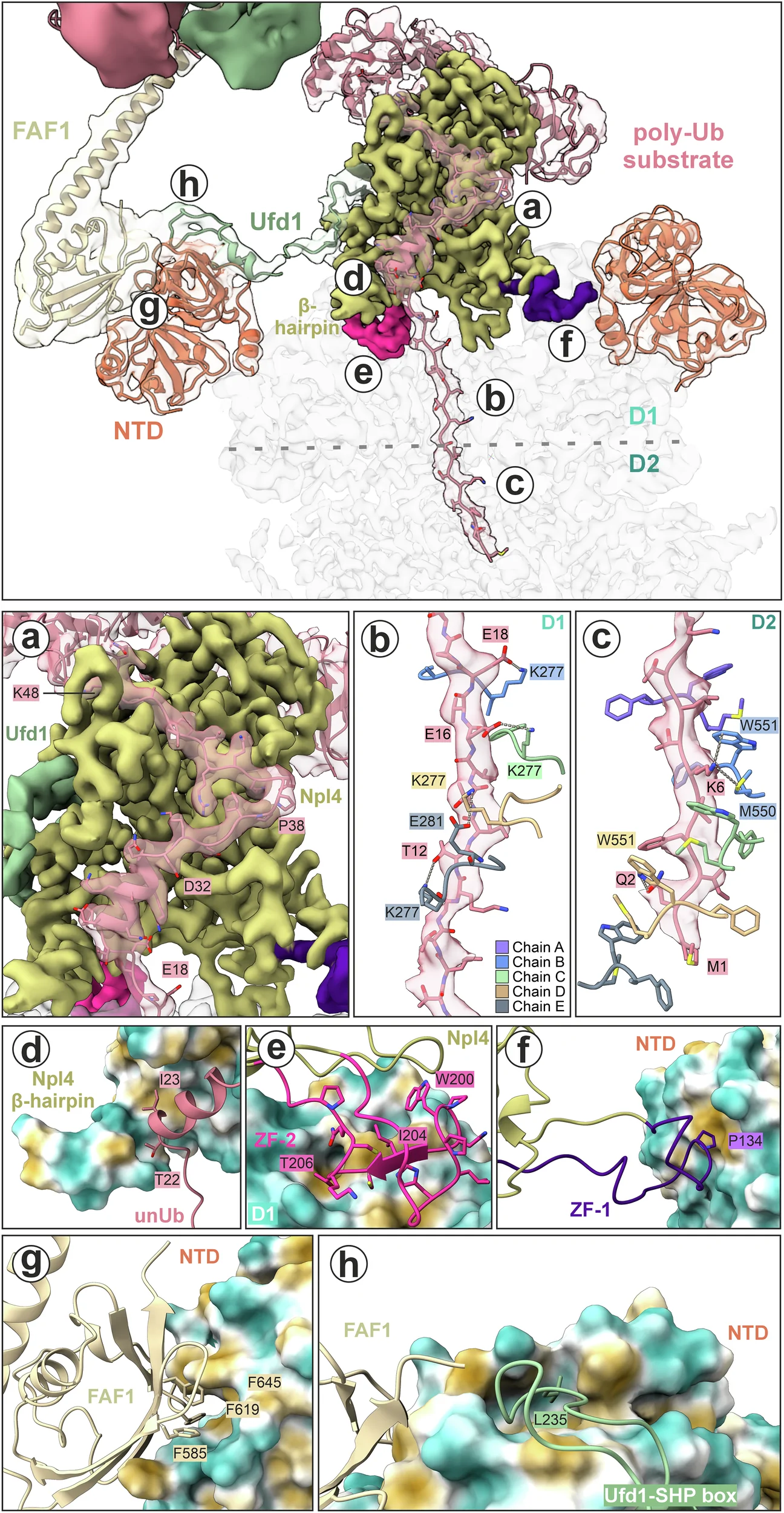
Interactions between UN, the initiator Ub and p97. The upper panel shows a close-up of the complex focusing on the interactions between Npl4, the initiator Ub, Ufd1, FAF1 and the NTDs of p97. The D1 and D2 rings of the ATPase are shown in transparent. ZF1 and ZF2 of Npl4 are colored in magenta and pink, respectively. Key interactions are labeled (**a–f**). Detailed views of the individual interaction sites are shown in the respective **a** Density and model of initiator Ub in the Npl4 groove **b**) Interactions between negatively charged residues of the translocating peptide and K277 of interacting pore loops in the D1 ring. **c** Interactions between the translocating peptide and the pore loops in the D2 ring. **d** Interaction between the folded helix of the translocating initiator Ub with the β-hairpin of Npl4 above the channel of the D1 ring (shown as surface and colored by hydrophobicity). **e,f** Interactions of ZF1 and ZF2 (magenta) with the D1 subunit B and the NTD of subunit D, respectively. **g**,**h** Detailed view of the interactions of the NTD of subunit A with the SHP box of Ufd1and UBX-domain of FAF1. The NTD is represented by surface and colored by hydrophobicity. The State II consensus map (EMD-58590) shows p97, while locally refined maps show the UN tower (EMD-58591) and the FAF1–UT3–proximal ubiquitin region (EMD-58592). Density is contoured at 15.7σ for FAF1-UT3-proximal ubiquitin region, 6.4σ for UN tower, and 6.6σ for p97.

We observe density of the initiator Ub extending across the Npl4 groove all the way through the pore to the D2 ring of the ATPase (Fig. 2). Most of the interactions between Npl4 and initiator Ub are similar to the yeast homolog Cdc48. Nevertheless, the resolution of the density map is superior to that of the yeast complex^10^, allowing modeling of the side chains from residue K48 (branch point of the initiator Ub at the beginning of the Npl4 groove) up to the N-terminal residue Met1, which is located directly below the pore loop II of subunit F in the staircase in the D2 ring, confirming that we successfully locked the complex at an early stage of substrate processing. Similarly, in the yeast homolog^10^, a characteristic kink is featured in the Npl4 groove where the single helix of the Ub (I23-Q31) remains folded (Fig. 2a). Notably, the lower end of this helix is stabilized by the β-hairpin motif 425-439 of Npl4. Specifically, T22 and I23 of initiator Ub snugly fit into two complementary pockets formed by the hairpin, which not only stabilizes the helix but also positions it precisely at the entrance of the ATPase’s central pore (Fig. 2d). Consistent with the functional importance of this interface, mutations that disrupt groove–substrate interactions (E446K + Y447A) significantly impair substrate unfolding in the Eos assay (Supplementary Fig. 7a, b). Given that these pockets exhibit shape complementarity to small, non-polar residues, with the upper pocket being hydrophobic, it is tempting to speculate that this interaction may be disrupted, once bulkier and/or charged residues are encountered during substrate processing, potentially leading to disengagement of the β-hairpin and repositioning of the substrate.

Immediately after entering the pore, the unfolded substrate first engages with pore loop I at the D1 ring of the ATPase. Notably, like its yeast counterpart^10^, the D1 ring pore loops assume a spiral staircase arrangement with loops from subunits B, C, D, and E interacting with the unfolded ubiquitin chain. As previously reported^12^, the D1 ring pore loops (L278, A279) primarily bind to the backbone of the unfolded peptide. We then examined the specific role of the side chains of initiator Ub in the central channel within the context of the interaction network. Remarkably, our analysis reveals that residues E16, E18 and T12 of the Ub chain form specific contacts with K277 of D1 pore loop I in chains B, C, and E (Fig. 2b). However, at those contact sites where the Ub chain lacks a residue with a formal negative charge at the interaction site with pore loop I (chain D), K277 is retracted and instead participates in intersubunit interactions, notably with E281 (chain E). Consequently, K277 in the D1 ring pore loops mediates additional interactions with substrate segments bearing negatively charged residues, thereby enhancing grip on the substrate.

Finally, the N-terminus of the initiator Ub engages with the five D2 ring pore loops (chains A-E), which are arranged in a staircase, with subunit A at the top and subunit E at the bottom, while subunit F is disengaged from the substrate. The D2 ring pore loops interact tightly with the substrate backbone via hydrogen bonds involving M550, W551, and F552 (Fig. 2c). Although we detect additional interactions at positions with large side chains, such as between Ub K6 and W551 of p97 chain B and Ub Q2 and W551 of p97 chain D, the sequence-independent interactions with the backbone dominate, suggesting that these side-chain interactions play a rather minor role in substrate processing efficiency.

#### Interactions of Npl4 with p97

The attachment of Npl4 to the D1 domain of p97 is mediated by a single zinc finger domain (ZF-2) and the β-hairpin (Fig. 2d), whereas its UBXL domain interacts with the NTD of subunit C. Notably, ZF-2 inserts into a hydrophobic groove of subunit B within D1 ring with its conserved residues W200, I204 and T206 (Fig. 2e). As a result of this tilted configuration, ZF-1 is excluded from interacting with the D1 ring, instead fitting into a hydrophobic pocket of the NTD of subunit D, where it forms a tight association (Fig. 2f). Although this state yielded the highest resolution, our analysis revealed three additional conformational states of Npl4, featuring distinct interfaces that vary depending on the D1 ring configuration, which are discussed in greater detail in the following sections.

#### Interactions of FAF1 with UN and p97 at the level of the D1 ring

The UBX domain of FAF1 forms a tight interaction with the NTD of subunit A of p97 (Fig. 2g), consistent with previous crystal structures of the individual domains^34^. Notably, we observed additional density on the NTD that interacts with FAF1, which is absent in the NTDs of p97 not bound to FAF1. Closer inspection and local refinement revealed that this density is connected to the NBM region of Ufd1, which is tightly associated with Npl4. This suggests that the density corresponds to the SHP box of Ufd1, which is located upstream of the NBM region, confirming an interaction that has been previously identified in the absence of FAF1^35^. The SHP box is stabilized by an interaction between residue L235 and a hydrophobic groove in the NTD domain of p97, which is in direct proximity to the binding site of FAF1’s UBX (Fig. 2h). Thus, this interaction effectively bridges Ufd1-Npl4 to the NTD of p97, which also binds FAF1. This spatial arrangement implies a synergistic binding mechanism between UN and FAF1 to p97, and thus explaining the 1:1:1 stoichiometry of the complex.

In summary, the UN adapter complex establishes direct connections with three NTDs of p97: specifically, the NTD of subunit D is linked via ZF1 of Npl4. The NTD of subunit C is bound by the UBXL domain of Npl4, and a third NTD of subunit A is engaged by the SHP motif of Ufd1; notably, this latter NTD also interacts with the accessory adapter FAF1 through its UBX domain.

### FAF1 collaborates with Ufd1 in poly-ubiquitinated substrate engagement and processing

**Fig. 3:**
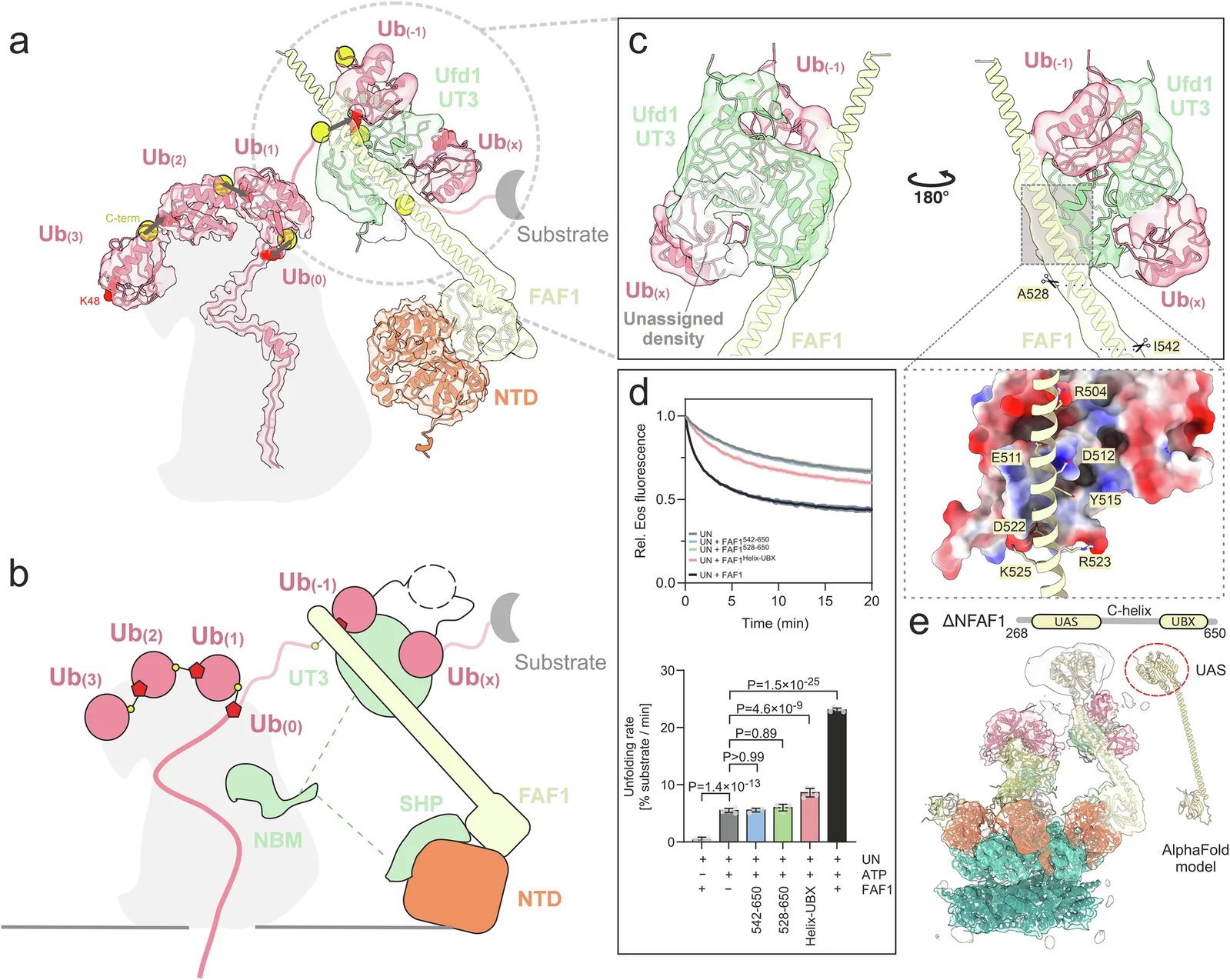
Ubiquitin molecules bound to Npl4, Ufd1 and p97. **a** Side view of the cryoEM volume and fitted models with Npl4 and p97 removed (except the NTD interacting with FAF1). Position of K48 and the approximate position of the flexible C-term in each Ub are indicated (K:48 shown in red spheres; C-term as yellow circle). We employ a numbering convention for Ubs, where positive numbers (+1, +2, etc.) denote Ubs that are N-terminal to the initiator Ub and distal to the substrate, whereas negative numbers (-1, x) indicate Ubs that are proximal to the substrate. **b** Cartoon representation of the polyubiquitin chain and its interactions with FAF1/Npl4 and Ufd1. **c** Magnified view of the boxed area in (**a**) with the AlphaFold3 prediction of FAF1 C-helix – UT3 and 2 Ub moieties rigid body fitted into the cryoEM volume. Mutated amino acids for the functional data are annotated. The panel shows a detailed views of the interaction between UT3 and FAF1. **d** p97 unfoldase activity assay. Loss of fluorescence of the polyUb-Eos substrate was followed during p97-mediated unfolding in the presence of UN without FAF1 or FAF1 full-length or truncations, as indicated upon addition of ATP (t = 0). Bar graph shows quantification of initial rates. Significance was tested by one-way ANOVA with Tukey test. Means ± SD, N ≥ 4 where N is the experimental repeats. Each assay was pipetted individually with the same batch of purified proteins. **e** Cryo-EM structure of substrate-engaged p97 in complex with Ufd1-Npl4 and the C-terminal domain of FAF1, extended by the UAS domain (residues 268-650). Note that this structure includes the UAS domain, whereas other structures presented in this manuscript lack this domain. An AlphaFold3 prediction of the FAF1 variant is shown, with the UAS domain highlighted with a red circle, alongside a schematic representation of the domain organization. The State II consensus map (EMD-58590) is complemented by locally refined maps showing the unUb and distal ubiquitins (EMD-58591) and the FAF1-UT3-proximal ubiquitin region (EMD-58592). Filtered density of substrate-engaged p97 in complex with Ufd1-Npl4 and the C-terminal region of FAF1 extended by the UAS domain (EMD-58586). Density is contoured at 15.7σ for the FAF1-UT3-proximal ubiquitin region, 6.2σ for UN tower, 6.6σ for p97, and 1.69σ for ΔNFAF1.

We conducted classifications and local refinements focusing on the C-helix of FAF1, revealing a density of four regions located in the segment 496–523 of the FAF1 C-helix. Although direct assignment of the central domains, which exhibit extensive interactions with the C-helix, proved challenging, the two peripheral regions were directly identifiable as Ub moieties based on their distinctive characteristics. Notably, the lower Ub molecule interacts with the central region, whereas the upper Ub molecule engages with both the central domain and the FAF1 C-helix directly. A plausible candidate for the central region is the UT3 domain of Ufd1, a segment that was not included in the high-resolution regions of the cryoEM density, known to interact with K48-linked Ubs at the substrate’s proximal site^23^. To further investigate this, we employed AlphaFold3^36^ to predict the structure of the C-helix FAF1, UT3, and 2-5 Ub molecules (Supplementary Fig. 8), followed by rigid-body fitting of the models into the cryo-EM density (Fig. 3a–c). To validate the predicted ubiquitin-binding interfaces, we introduced site-specific mutations in Ufd1 that are predicted to disrupt interaction with the Ub(-1) and Ub(x) sites, which resulted in a marked reduction in substrate unfolding without affecting UN complex assembly (Supplementary 7b,d, 9). The resulting model shows good overall agreement with the cryo-EM data without additional adjustments, confirming that the central region is indeed occupied by the UT3 domain of Ufd1.

Thus, Ufd1 establishes a dual linkage between substrate and FAF1: firstly, through its UT3 domain, Ufd1 engages with Ubs and forms a stable interaction with the C-helix of FAF1; secondly, the SHP-motif box of Ufd1 binds to the same NTD that also interacts with the UBX domain of FAF1 (Fig. 3a, b).

The interface between UT3 of Ufd1 and the C-helix of FAF1 shows extensive electrostatic interactions (Fig. 3c). Thus, the primary role of the C-helix of FAF1 is to position the UT3 domain of Ufd1, which in turn engages Ubs of the substrate. Consistent with this, FAF1 mutants V507A/K508A, which disrupt this interface, show reduced substrate unfolding, providing direct evidence of the importance of this interaction^37^. Importantly, biotinylation-based pulldown assays indicate that both full-length FAF1 and C-terminal truncations bind p97 with comparable efficiency (Supplementary Fig. 10), suggesting that the observed functional defects are not due to impaired complex formation. Furthermore, by deleting the segment of the C-helix that, according to our structure, engages UT3 and the upper Ub, the function of FAF1 in enhancing substrate processing by p97/UN is abolished (Fig. 3d).

Because the structure visualized additional Ubs beyond the initiator and those crowning Npl4, we revisited the arrangement of the polyubiquitin chain on the cis-side of the complex. We closely examined the positions of K48 branchpoints and C-termini of the Ubs. Our analysis revealed that the three Ubs associated with Npl4 are sequentially arranged, with the C-terminus of each preceding ubiquitin oriented towards the K48 residue of the subsequent one, indicating that they form consecutive components of the polyubiquitin chain. Notably, the C-terminus of the most proximal Ub points directly towards the K48 residue of the initiator Ub, suggesting a linkage between them. Based on this arrangement, we designate the initiator Ub as Ub(0) and the three distal Ubs bound to Npl4 as Ub(1), Ub(2), and Ub(3), reflecting their N-terminal positioning within the chain (Fig. 3a, b).

The C-terminus of the initiator Ub(0) is expected to point towards the K48 residue of the preceding Ub(-1), which is proximally located to the substrate. However, the absence of the C-terminal 26 residues of Ub(0) from our density map suggests that this region is partially unfolded. Under this assumption, the flexible C-terminus of Ub(0) may span the ~40 Å distance to the upper Ub anchored to the FAF1 C-helix, supporting its designation as Ub(-1). In contrast, a direct linkage between the C-terminus of Ub(0) and the lower ubiquitin bound to UT3 is improbable due to the substantial distance (>70 Å) separating them, as well as the orientation of the K48 residue, which points in the opposite direction (Supplementary Fig. 11a).

Furthermore, the positioning of the C-termini and branchpoints of the upper and lower Ubs at the FAF1 C-helix reveals that they are non-sequential, implying the presence of at least one intervening ubiquitin between them. We therefore denote the lower Ub as Ub(x). Additionally, a small, unassigned density is observed directly at the C-terminus of Ub(x) (Fig. 3c), which could potentially represent the previous Ub in the polyubiquitin chain or the Npl4 zinc finger (NZF) that recognizes Ubs^38^.

We acquired an additional cryo-EM dataset under identical conditions using a FAF1 variant that extended our previous construct by incorporating the UAS domain, in addition to the UBX domain and C-helix (Fig. 3e and Supplementary Fig. 12). Despite the low resolution, subsequent processing revealed a clear globular density at the expected position atop the C-helix, although the region exhibited high flexibility. Rigid-body fitting of the AlphaFold3 prediction of this FAF1 variant into the cryo-EM volume confirmed the topology of the UAS domain at the end of the C-helix (Fig. 3e). Notably, the UAS domain did not exhibit interactions with Npl4 or the distal Ubs on Npl4. The globular density featured a central domain consistent with the size of UAS and two peripheral densities matching the size of Ubs, but the limited resolution precluded definitive identification. Supporting this observation, AlphaFold3 predictions suggest that UAS might bind Ubs (Supplementary Fig. 8), albeit with medium confidence.

### Dynamic interplay of FAF1 and UN during p97 substrate processing

**Fig. 4:**
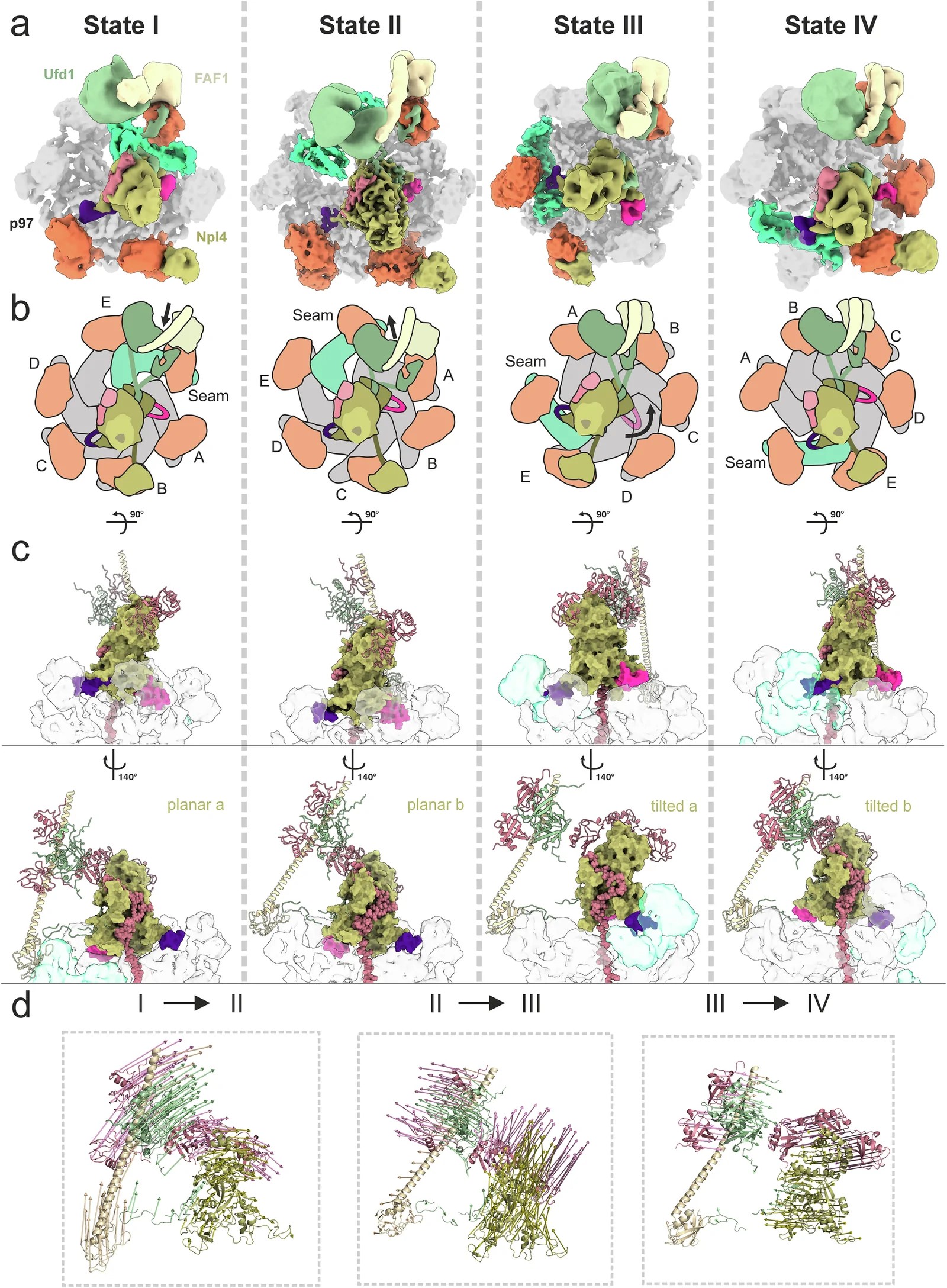
Four states of substrate engaged p97-Ufd1-Npl4-FAF1. **a** Top-views of cryo-EM volumes of states I-IV sorted according the position of the seam subunit. Note that distal and proximal ubiquitins are omitted here for better clarity. NTDs of p97 involved in interactions are shown in orange. **b** Cartoon representations of (**a**) with green lines indicating the Ufd1 linkers to the SHB box and UT3 and a khaki line indicating the Npl4 linker to UBXL (not resolved in our densities). **c** Surface representation of Npl4 and initiator Ub, together with ribbon representation of FAF1, UT3, Ufd1, proximal and distal Ubs, shown for all four states, viewed from two distinct perspectives. ZF1 and ZF2 of Npl4 are colored in purple and pink, respectively. **d** The panel shows a ribbon representation of FAF1, UT3, Ufd1, Npl4 proximal and distal Ubs, together with arrows indicating the direction of motion when transitioning from one state the next one. State I: p97–FAF1 consensus map (EMD-58587; 6.37σ); locally refined UN tower map (EMD-58588; 6.7σ). State II: p97 consensus map (EMD-58590; 7.8σ); locally refined UN tower map (EMD-58591; 6.2σ); locally refined FAF1 map (EMD-58592; 15.7σ). State III: p97-FAF1 consensus map (EMD-58594; 4.05σ); locally refined UN tower map (EMD-58595; 5.27σ). State IV: p97-FAF1 consensus map (EMD-58597; 5.13σ); locally refined UN tower map (EMD-58598; 4.68σ).

We performed extensive classifications and local refinements focusing on UN, which revealed four distinct states of the complex (Supplementary Figs. 5, 13). We compared and aligned the states carefully, taking into account the conformational transitions of the p97 hexamer during the ATPase cycle according to the leading model of the hand-over-hand mechanism (Fig. 4). In accordance with previous studies^10,12^, subunits A–E interact with the initiator Ub chain through pore-loops, forming a staircase-like configuration, with subunit A at the top and subunit E at the bottom. The sixth subunit F (termed seam subunit) is disengaged from the substrate, makes less extensive interactions with its neighboring subunits, and is localized between the ends of the staircase.

All four states exhibit similar overall features with respect to the poly-Ub chain, as described earlier, but differ in their ATPase states and UN conformations. These states were categorized into four distinct phases (I, II, III, IV) based on the position of the seam, reflecting successive stages of the p97 motor during the ATPase cycle. The nucleotide binding pockets of subunits A-E in both D1 and D2 rings contain density consistent with ADP.BeF(x), whereas the seam subunit F, although less resolved, shows density corresponding to ADP (Supplementary Fig. 14). Viewed from the cis-side, ATP hydrolysis proceeds in a clockwise direction, resulting in the anti-clockwise subunit becoming the seam after each cycle. The highest resolution was achieved for state II (3.1 Å for p97 and Ufd1-Npl4, and an average resolution of 8 Å for FAF1 and proximal Ubs; described in previous sections and shown in Figs. 1–3), while reconstructions of states I, III, and IV reached average resolutions of 4–10 Å in the region of UN and FAF1. These resolutions nonetheless enabled unambiguous fitting of the models for all components (Npl4, Ufd1, FAF1, and substrate) obtained from modeling state II. Top-down views of the reconstructions and corresponding cartoons are presented in Fig. 4a, b, respectively. Note that, for clarity, distal and proximal Ubs have been omitted from this representation, while complete reconstructions are provided in Supplementary Fig. 15.

Across all states of the ATPase cycle, FAF1 remains bound on the same NTD of p97 via its UBX domain, and in particular it remains bound on the same NTD as the Ufd1-SHP box. Ufd1 (incl. the UT3, SHP-box and NBM domains) consistently orients towards FAF1, while the opposing face of UN, featuring the ZF-1 and UBXL domain of Npl4, is exclusively positioned on the opposite hemisphere of the ATPase relative to FAF1 (Fig. 4a, b). This spatial arrangement is consistent with our findings, described in the previous sections, which revealed a dual linkage between Ufd1 and both FAF1 and the NTD bound to FAF1. Specifically, the SHP box of Ufd1 interacts with the NTD bound to FAF1, while the UT3 domain of Ufd1 binds to the C-helix of FAF1. Furthermore, the polyUb chain provides an additional point of connection, with proximal Ubs binding to UT3 and the FAF1 C-helix, and distal Ubs capping Npl4 (Supplementary Fig. 11). Based on these findings, we conclude that FAF1 remains bound to Ufd1 and the same NTD, with its flexible connections constraining the overall orientation of the UN complex, thereby limiting its rotational freedom, while still permitting adaptive responses to the conformational dynamics of the staircase during the ATPase cycle, as further elaborated below.

In state I, FAF1 is anchored to the NTD of the seam subunit of the ATPase, while UN assumes a slightly tilted state relative to the axis of the D1 pore, with both Npl4 ZF domains engaged with p97 (Fig. 4c). Specifically, ZF-1 interacts with the NTD of subunit C, and ZF-2 binds to the D1 ring of subunit A. Concurrently, the UBXL domain of Npl4 associates with the NTD of subunit B, two subunits clockwise from the NTD binding FAF1.

#### Transition state I → II

During the transition to state II, the p97 seam subunit transitions to subunit A, and thus relocates from an intermediate position to the apical position at the top of the spiral staircase. There is thus a 26 Å upward movement of the whole subunit, including the NTD binding FAF1 (Fig. 4). This movement is transmitted to the UBX domain of FAF1, the C-helix and also to the connecting domains to UN, SHP and UT3, which finally results in a minor conformational change of UN, while it retains its overall orientation (Fig. 4c, d). Despite the transition, UN maintains its slight tilt, with both ZF domains remaining engaged with p97. However, the ZF interfaces undergo a conformational adjustment to accommodate the new ATPase configuration (Supplementary Fig. 16). Notably, ZF1 snuggles into a groove formed by the NTD of subunit D and the D1 ring interface between subunits D and E, while ZF2 remains bound to the D1 of subunit A.

#### Transition state II → III

The most pronounced conformational changes occur during the transition from state II to state III (Fig. 4). This is because the seam subunit shifts to the anti-clockwise position, disrupting the interface between ZF1 of Npl4 and p97. Notably, the previous ZF1 binding site, which involved subunits D and E, is dismantled as subunit E becomes the new seam, rises, and loses its contacts with neighboring subunits in this region. Importantly, ZF1 establishes a new interface with p97, snuggling into a groove formed between the NTD and D1 of the seam subunit (Supplementary Fig. 16). This interaction induces a ~ 60° clockwise rotation and an ~20 Å elevation of the UN tower (Npl4 and NBM domain of Ufd1), following the relocation of the rising seam subunit. ZF-2 dissociates from p97 and is unbound in state III, leading to a highly tilted conformation that bears resemblance to the previously described seesaw conformation^25^, albeit in a distinct context (in absence of FAF1 and a planar conformation of p97). Notably, this conformational change is transmitted to FAF1, UT3, and the ubiquitin chain, causing the latter to rotate in tandem with the movement of UN and the FAF1 C-helix and UT3 to swing out approximately 13 degrees (Fig. 4c, d). Surprisingly, despite its flexible linkage to UN, the UBXL domain of Npl4 accompanies the rotational movement of UN and repositions itself to the NTD of the clockwise adjacent subunit compared to its position in State II. Meanwhile, the Ufd1 SHP box remains anchored to the same NTD that interacts with the UBX domain of FAF1.

#### Transition state III → IV

During the transition to state IV, once again the sole interface between UN and the p97 seam is disrupted (Fig. 4). In response, UN reverses its rotation ~40 degrees, returning to the orientation of state II and simultaneously, ZF-1 engages with the groove between the NTD and D1 of the new seam subunit, adopting a similar interface to that observed in state III. UN retains its highly tilted configuration, with ZF2 interacting with the NTD of the anti-clockwise adjacent subunit (Fig. 4c, d). Notably, the UBXL of Npl4 domain follows the rotational motion of Npl4, repositioning to the NTD situated two subunits clockwise from the NTD that binds FAF1, thereby restoring its original location.

The lower resolution of FAF1, relative to the other components of the complex, suggests increased heterogeneity in this region. To investigate this further, we performed 3D variability analysis for each state, focusing on FAF1, the Ufd1-UT3 domain, and the proximal ubiquitins (Supplementary Fig. 17). The analysis succeeded for states II, III, and IV and reveals a continuous conformational trajectory that involves FAF1, the Ufd1-UT3 domain, and the proximal ubiquitins. In particular, FAF1 undergoes a continuous tilting motion across the different states, supporting a continuous local conformational trajectory, while Npl4 and p97 retain their distinct state-specific conformations. Interestingly, the continuous motion in state II points in the direction of state III and subsequentially from state III to state IV, consistent with a sequential state transition.

## Discussion

p97/Cdc48 processes substrates via a ‘hand-over-hand’ mechanism^9,12,13,39^, whereas its primary adapter for polyubiquitylated substrates, Ufd1-Npl4 (UN), plays a crucial role in substrate engagement and initiating ubiquitin unfolding^10^. Extensive research has begun to shed light on the interactions between p97/Cdc48 and its adapters and accessory adapters^40,41^. However, we still lack mechanistic insights into how p97 and its adapters collaborate during substrate processing, including the conformational dynamics of UN throughout the ATPase cycle and the role of accessory adapters such as FAF1 in assisting p97-UN function. A major challenge in studying this process lies in the complexity of the ATPase motor, which involves conformational changes of the spiral that are likely transmitted to the UN anchored on the cis-side of the complex. Furthermore, the N-terminal UT3 domain of Ufd1 recruits poly-ubiquitinated substrates^27,42^, a process that is expected to be highly dynamic due to the flexibility of the poly-ubiquitin chain and flexible connection of UT3 to UN. How UT3 engages the proximal Ubs has not yet been experimentally confirmed. The involvement of accessory adapter proteins such as FAF1 adds another layer of complexity to the system, making it even more challenging to understand how this sophisticated machinery interacts with its adapters and how different accessory adapters influence the process. However, recent breakthroughs in cryoEM offer avenues for investigating this highly dynamic process. Leveraging a cutting-edge cryoEM setup with up to a 10-fold increase in throughput compared to previous-generation instruments, we have collected a comprehensive dataset of 280,000 movies to elucidate the coordinated action of p97, UN, and FAF1 in engaging and threading a poly-ubiquitinated substrate. Although such a large-scale dataset posed significant practical challenges, including data acquisition, processing time, and computational demands, it enabled us to capture multiple conformational states of p97-UN-C terminal FAF1 complex.

Our structural analysis reveals that FAF1 does not bind to an unoccupied NTD of p97; instead, it specifically binds via its UBX domain to the NTD of p97 that is also occupied by the SHP-box of Ufd1, which establishes a direct link between FAF1 and UN. However, it remains uncertain whether FAF1 binding to the NTD induces a conformation that is preferentially recognized by the SHP-box or if binding of UN to p97 alters the conformation of this NTD via the SHP-box, thereby facilitating FAF1 binding. Notably, the UBX domain of FAF1 extends a C-helix towards the Npl4 top, recruiting UT3 of Ufd1 and creating another direct interaction between FAF1 and UN. Both linkages are situated on the same side of Npl4, and despite the flexibility of the linkers to UT3 and the SHP-box, they collectively constrain the overall orientation of the UN tower, thereby limiting large-scale rotations during the ATPase cycle, while still allowing for the required dynamics.

Of note, the C-helix of FAF1 positions UT3 at the same level above the p97 pore as the trio of distal Ubs capping Npl4. Our analysis shows that the C-helix of FAF1 and UT3 bind to a Ub molecule (designated as Ub(-1)) close to the C-terminus of the initiator Ub, suggesting it is proximal within the ubiquitin chain. A second Ub is observed to bind to the underside of UT3, suggesting its position is further proximal in the polyubiquitin chain due to its orientation. The polyubiquitin chain, despite its inherent flexibility, most likely acts as an additional tether between UN and FAF1, reinforcing their relative orientation.

Thus, the FAF1 structure resembles a sturdy anchor rod, anchored to an NTD of p97 and connected to the UN complex via two flexible linkers and the polyUb chain. Meanwhile, Npl4 balances precariously atop the spiral staircase, grasped by its ZF clamps. This distinctive design allows FAF1 to restrain UN’s rotational movement, while still permitting the UN complex to exhibit remarkable flexibility and adaptability in response to the conformational shifts of the ATPase motor, including the up-and-down movements of p97 subunits relative to each other that are part of the motor’s function.

Similar seesaw conformations of UN, previously described^25^ as crucial for substrate unfolding, are now contextualized within the ATPase cycle. The multiple interactions established by FAF1 apparently further stabilize UN-p97 interactions, enabling the determination of Npl4 in distinct conformations throughout the ATPase cycle. This was unattainable in this previous study, which lacked FAF1 and could not assign specific ATPase staircase configurations to distinct UN states.

We identify two primary conformations of Npl4 relative to the spiral staircase: planar and tilted, each with two substates (planar a, planar b, tilted a, and tilted b) (Fig. 4c) that correspond to states I, II, III, and IV of the complex. Notably, the planar conformations occur in two consecutive steps of the ATPase cycle (states I and II), when both ZFs of Npl4 are not involved in interactions with the seam subunit. During this transition (Fig. 4, State I → II), Npl4 maintains its overall orientation and remains anchored to p97 via both ZF domains, which interact with adjacent subunits of the spiral staircase that are engaged with the substrate. These subunits are tightly associated without significant height differences. Upon arrival of a seam subunit at the ZF-1 position of UN (Fig. 4, State II → III), the seam subunit undergoes a significant conformational shift, elevating and disengaging from its adjacent subunit, which abolishes the ZF-1 interface. In response, the UN conformation must adapt to accommodate this substantial change. ZF-1 exhibits a strong affinity for a groove on the seam and binds to it, whereas ZF-2 on the opposing side simultaneously disengages from p97, accompanied by a pronounced tilting motion. As a result, the entire UN complex follows the seam, elevates approximately 20 Å and rotates by approximately 60°. In the next step (Fig. 4, State III → IV), the seam becomes subunit A, and the ZF-1 interface is disrupted again, so ZF-1 snuggles into the same preferred interface in the groove of the now anti-clockwise positioned seam, thereby rotating back to its previous orientation, while staying in a tilted conformation. Although the UN complex maintains its overall structure, it thus undergoes a coordinated tilting and rotational motion during transitions between planar and tilted states. This movement is driven by the preferential binding of Npl4’s ZF-1 to the seam, as well as changes in height and distance to adjacent subunits caused by seam disengagement, also leading to the disengagement of ZF-2. In all states of UN, the position of the unfolded peptide is well aligned with the ATPase pore. Although we define states I–IV as distinct based on the conformations of p97 and Npl4, they should be interpreted as conformational ensembles rather than rigid structures. This is because the more flexible cis-components exhibit significant variability within each state. Specifically, FAF1, the Ufd1 UT3 domain, and the proximal ubiquitins follow a continuous conformational trajectory that is consistent with the directionality of the ATPase cycle. While it would be intriguing to visualize the continuation of the full ATPase cycle beyond state IV, the subsequent two states remained elusive, potentially due to the heightened flexibility of UN or the complex being trapped in its initial substrate-processing states. Nevertheless, the notion of an infinite rotation of UN, with ZF-1 binding to the next counter-clockwise seam, as seen between states III and IV, is untenable, as further rotation is hindered by the unidirectional SHP binding to the NTD of p97 and the UT3 binding to FAF1. Instead, we propose that UN reverts to its planar conformation as the seam moves to the next position, enabling UN to re-engage with both ZFs, whose subunits interact with the substrate, thereby facilitating a transition back to the planar state, while positioning the substrate atop p97’s pore.

Our results show that the conformational switch between planar and tilted states of UN is triggered by the seam subunit. Our reconstructions strongly suggest a continuous cycle of Npl4 conformations, comprising a planar→ rotate→ tilted-a→ backrotate→ tilted-b→ planar sequence, which is defined by the strong interface between ZF-1 and the seam subunit. Importantly, the rotations and elevations from planar → tilted are directly transmitted to the FAF1 rod, which swings out. Importantly, when the seam arrives at the NTD bound to FAF1, this motion is again transmitted to the FAF1 helix, which tilts back (Fig. 4d). Thus, there is a continuous swing-in- swing-out motion of the FAF1 helix throughout the ATPase cycle.

This observation is particularly noteworthy, as FAF1 appears to play a crucial role in engaging the polyUb-substrate by binding UT3 at a level aligned with the top of Npl4, thereby creating a continuous platform for the binding of proximal Ubs to the UT3 domain of FAF1 and distal Ubs on top of the Npl4 tower. These results suggest that precise positioning of the initiator Ub at the groove on top of Npl4 is critical, which likely facilitates the initiation of substrate processing and unfolding, explaining the higher unfolding rate observed upon addition of FAF1. Moreover, the alternating swing-in-swing-out motion of FAF1 may provide mechanical assistance to UN and p97 during progressive substrate processing, thus increasing processivity. This is evidenced by the observation that the C-helix of FAF1, upon tilting in, positions Ub(-1) in close proximity to the branch point of the initiator ubiquitin at the entrance of the Npl4 groove. Furthermore, our data reveal that the UAS domain of FAF1 extends the rod with a globular cap, exhibiting additional densities that may correspond to proximal Ubs, suggesting that it could serve as an auxiliary binding platform for the ubiquitin chain. The UAS domain and the additional densities do not appear to interact directly with Npl4 or UT3, but further investigation is required to fully elucidate the interactions. Following Ub(1), the initiator Ub could be resolved all the way from the Npl4 groove to the D2 ring to a resolution that allowed us for tracing and model building to study the interactions between side chains of the substrate with Npl4 and p97.

Our results provide detailed insights into how FAF1 and UN engage poly-ubiquitinated substrate proteins for processing by p97 and importantly, shed light on the dynamic and orchestrated interactions between p97, its adapter UN, and accessory adapter FAF1 during the ATPase cycle and substrate processing, providing mechanistic insights into how FAF1 ultimately boosts the efficiency of the process. Given the combinatorial diversity of p97-adapter complexes, our study offers a paradigm and a strong foundation for future research towards a molecular understanding of the emerging principles governing how different p97 accessory adapters and adapters collaborate with the ATPase motor to regulate its function.

## Methods

### Cloning

Human FAF1 with an N-terminal poly-His tag was cloned into pET28a via Gibson cloning. FAF1 truncations FAF1 ΔN (Δ1-270), C-terminal FAF1 (Δ1-480), FAF1 Δ1-527 and Δ FAF1 Δ1-541 were subsequently generated by Q5 site-directed mutagenesis. Human p97 from Addgene plasmid #23971 was cloned into pET15b with an N-terminal poly-His tag via restriction cloning and point mutations for E578Q and A232E were introduced via two rounds of Q5 site-directed mutagenesis. The ORFs for human Ufd1 and Npl4 were amplified from HeLa cDNA and inserted into pET41 by restriction cloning (Npl4: NdeI, EcoRI; Ufd1: NdeI, XhoI). Point mutations in His-Ufd1 and Npl4 were introduced by QuikChange site-directed mutagenesis or replacement of the region by gBlocks (IDT) with the desired mutations via Gibson cloning. Generation of pFL His-p97^11^ and pET28a His-Ub_2_V-mEos3.2^43^ was described previously. C-terminal TwinStrep tags were inserted via Gibson assembly cloning into pET41b Ufd1 and pET28a His-Ub_2_V-mEos3.2. All coding sequences in the resulting plasmids were verified by Sanger sequencing (Eurofins).

### Protein expression and purification

For the expression of recombinant human p97, the Baculovirus system or Rosetta2 *E. coli* cells were used^11^. Briefly, Sf9 cells (ThermoFisher, 11496015) were transfected with a pFL vector encoding His-tagged p97. After virus amplification in Sf9 cells, the final protein expression was performed in High Five cells (ThermoFisher, B85502) using a HisTrap FF column (Cytiva) in p97 buffer (50 mM HEPES pH 8.0, 150 mM KCl, 5 mM MgCl_2_, 5% glycerol) containing 25 mM imidazole, 1 mM PMSF and 1 mM benzamidine. Protein was directly eluted onto a HiTrap Q HP anion exchange column (Cytiva) using p97 buffer with 300 mM imidazole. p97 was then eluted using a gradient (Buffer A: p97 buffer, Buffer B: p97 buffer including 850 mM KCl) and further purified by size exclusion chromatography on a Superose 6 Increase 10/300 GL in p97 buffer supplemented with 1 mM DTT. Fractions containing p97 were collected, analyzed by SDS-PAGE, aliquoted, and stored at -80 °C.

In case of the bacterial system, Rosetta2 *E. coli* containing the desired plasmid were grown in Lysogeny Broth (LB) medium containing carbenicillin (100 μg/mL) with shaking at 37 °C to OD600 of 0.8. The culture was then cooled down at 18 °C and induced with 0.5 mM IPTG for 14–16 h, and harvested by centrifugation. The purification was carried out similarly to the insect cell-expressed protein. Ufd1-Npl4 variants, His-Ub_2_-Eos, His-Ub_2_-Eos-Strep as well as FAF1 variants were recombinantly expressed in *E. coli* BL21 and lysed as described for p97. For His-Ub_2_-Eos, FAF1 variants, cleared lysates were passed through a HisTrap column (Cytiva), the column was washed with p97 buffer supplemented with 20 mM imidazole and bound protein was eluted with p97 buffer containing 300 mM imidazole. For Ufd1-TwinStrep and untagged Npl4, lysates were combined after lysis. Cleared lysates were passed through a StrepTrap XT column (Cytiva), the column was washed with p97 buffer and bound protein was eluted with p97 buffer containing 50 mM biotin. For Ufd1-Npl4, His-Ub_2_-Eos and FAF1 variants, the eluate was subsequently purified by size-exclusion chromatography using a Superdex 200 16/600 gel filtration column (Cytiva) with p97 buffer containing 1 mM DTT. Purified His-Ub_2_-Eos was irradiated with a 365 nm long-wave UV lamp (UVP Blak-RayTM B-100AP) for 4 × 30 min with 30 min breaks at 4 °C to induce a backbone break in mEos3.2.

Ubiquitin and ubiquitin mutants were expressed and purified as described^43^. Briefly, untagged ubiquitin was expressed in bacteria, and cleared lysates were mixed with 70% perchloric acid, centrifuged for 20,000 g for 1 h and the supernatant was dialyzed in ubiquitin wash buffer (25 mM ammonium acetate pH 4.5), loaded onto a 5 ml HiTrap SP HP column (Cytiva) and gradually eluted using 250 mM ammonium acetate 200 mM NaCl pH 7.6. The protein was loaded onto a Superdex 75 16/600 column (Cytiva) equilibrated in p97 buffer and peak fractions were snap-frozen and stored at -80 °.

### Substrate generation

His-Ub_2_V-mEos3.2 was poly-ubiquitinated as previously described^43^. Briefly, the substrate (10 µM) was mixed with Strep-mUbe1 (2 µM), gp78-ubc7 fusion protein (20 µM) and ATP (10 mM) in ubiquitination buffer. Ubiquitin was added stepwise over the course of 4 h incubation at 37 °C up to a final concentration of 400 µM and the reaction was further incubated overnight. The ubiquitinated His-Ub_2_n-Eos was isolated from the mixture by affinity purification using a HisTrap column (Cytiva). After washing with p97 buffer with 20 mM imidazole, the protein was eluted with p97 buffer containing 300 mM imidazole, followed by size exclusion chromatography using on a Superdex 200 16/600 column (Cytiva) equilibrated with p97 buffer. Fractions with at least 10 attached ubiquitin molecules to the substrate were identified by SDS-PAGE, concentrated and used for experiments.

### Unfolding assay

Polyubiquitinated substrate (Ub_2_(n)-Eos, 35 nM), p97 (175 nM hexamer), Ufd1-Npl4 (500 nM) and FAF1 variants (500 nM) were mixed in unfolding buffer (25 mM HEPES, pH 7.4, 100 mM KCl, 5 mM MgCl_2_, 5 % glycerol, 1 mM DTT) and incubated for 5 min at 37 °C. The unfolding reaction was started by addition of ATP (2 mM) and loss of red mEos3.2 fluorescence (excitation: 540 nm; emission: 580 nm) was monitored using a Cary-Eclipse fluorescence spectrophotometer (Varian). Rates were determined in GraphPad Prism from the slope at t = 0 by one-phase nonlinear regression over the subsequent 5 min. For reactions without FAF1, the initial rate was determined by linear regression over the first 5 min.

### Biotinylation of FAF1 variants

FAF1 and FAF1 truncations were biotinylated using EZ-Link NHS-PEG4-Biotin (ThermoFisher). FAF1 variants (10 mg/ml) were incubated in p97 buffer with 3-fold molar excess of NHS-PEG4-Biotin for 45 min at room temperature before quenching by addition of 50 mM Tris pH 7.5 for 45 min at room temperature. Excess biotin and quencher were removed using 0.5 ml Zeba Spin desalting columns (ThermoFisher) equilibrated in p97 buffer.

### Pulldown assays

For FAF1-p97 interaction analysis, biotinylated FAF1 variants (2 µM) were incubated with p97 (2 µM) and streptavidin agarose (Cytiva) in p97 buffer for 45 min at 4 °C. Beads were washed 3x in wash buffer (50 mM Tris pH 7.4, 50 mM KCl, 5 mM MgCl_2_, 5% glycerol, 1 % Triton X-100, 2 mM β-mercaptoethanol) and eluted with Laemmli sample buffer at 95 °C.

### Mass photometry analysis

Stoichiometry of the p97-Ufd1-Npl4-FAF1 complex was determined using a TwoMP mass photometer (Refeyn). To stabilize the complex at low nanomolar concentrations, p97 (10 µM hexamer), Ufd1-Npl4 (10 µM) and FAF1 (30 µM) were crosslinked with 625 µM Disuccinimidyl Sulfoxide (DSSO) for 45 min at room temperature. Samples were quenched by addition of 100 mM Tris pH 7.5 for 15 min at room temperature. For mass photometry measurements, samples were diluted to 25 nM p97, 25 nM Ufd1-Npl4, 75 nM FAF1 in DPBS (Pan Biotech), spotted on uncoated MassGlass UC carrier slides (Refeyn) and movies were recorded for 1 min using the large image size settings in the AcquireMP software (Refeyn). MassFerence P1 (Refeyn) was used as calibrant. Binning (10 kDa) and analysis were performed using the DiscoverMP software (Refeyn).

### Ubiquitin chain type assays

For ubiquitin chain analyses, His-Ub_2_V-mEos3.2 was poly-ubiquitinated enzymatically as described above. After 1 h of the ubiquitination reaction, samples were added to Strep-Tactin Sepharose (IBA Lifesciences) at 4 °C for 1 h to remove unanchored chains and ubiquitination enzymes. Beads were washed 3x in wash buffer and eluted with Laemmli sample buffer at 95 °C before SDS-PAGE analysis.

### Cryo-EM sample preparation and data acquisition

For structural studies, p97 expressed in bacterial cells was used. The complexes were reconstituted from frozen aliquots at room temperature. Proteins (Supplementary Table 1) were mixed in buffer containing 20 mM HEPES pH 7.5, 100 mM KCl, 5 mM MgCl_2_, 0.5 mM TCEP supplemented with 1 mM ADP, 1 mM BeSO_4_, and 4 mM NaF. Samples were incubated at room temperature for 30 min. To prepare the grids for cryo-EM, 4 μL of each sample solution (6–12 mg/mL) were applied to glow-discharged UltrAuFoil R1.2/1.3 grids (Quantifoil Micro Tools). Prior plunging, 0.5 μL of Triton-X (final concentration of 0.005%) were applied on the sample to diversify the particle orientations on ice. The grids were blotted for 4.5 s and plunged- frozen in liquid ethane after 0.5 s drain time in 100% humidity at 13 °C (Vitrobot Mark IV, Thermo Fisher Scientific). Datasets of the clipped grids were acquired using the EPU software with a Krios G4 Cryo-TEM (Thermo Fischer Scientific) at 300 kV, equipped with an E-CFEG, a Selectris X Energy Filter with a slit width of 10 eV, and Falcon 4i Direct Electron Detector.

### Cryo-EM data processing

All datasets and image processing steps were performed using CryoSPARC (v3.3-v4.6.2)^44,45^. The processing workflow involved frame alignment and dose-weighting was carried out using Patch Motion Correction, followed by CTF correction with Patch CTF. Particles were selected using with Blob picker and extracted at 2.03 Å/pix. They were subjected to several rounds of 2D classification to remove particles lacking distinct features.

For the dataset with only-C-term FAF1, the resulting 2,693,110 particles underwent to several rounds of heterogeneous refinement. The resulting classes included junk particles, particles containing p97 alone, and three distinct promising classes containing additional density corresponding to UN and only-C-term FAF1. Each class was then subjected to two rounds of 2D classifications and heterogeneous refinements to further clean the particle stacks. Next, each class was non uniformly refined and subjected to several rounds of 3D classifications. The particles of promising classes were then re-extracted at the final pixel size of 0.79 Å/pix and refined, yielding states I-IV. Local refinement was applied to specific regions of each state: for states I and III, density corresponding to Npl4 was locally refined, while for states II and IV, densities corresponding to Npl4 and FAF1 were refined separately. 3D variability analysis was performed using particles from consensus refinements for states II, III and IV using a filter resolution of 8 Å and solving for three components. Finally, for each state, locally refined regions were combined to generate composite maps using Phenix (phenix.combine_focused _maps^46^) (Supplementary Fig. 4). However, all figures depict the consensus reconstructions for p97 and locally refined maps wherever available, filtered according to their respective local resolutions. Please refer to Supplementary Table 4 and figure legends. The normalized and non-normalized composite maps are generated exclusively for uploading to EMBD and to allow visualization of the fitting in a single volume.

For the ΔΝ FAF1 dataset, initial 2D classification yielded 822,000 particles, which were subsequently subjected to 3D classification. Classes exhibiting density corresponding to ΔΝ FAF1 und UN bound to p97 were merged and refined non-uniformly. Following re-centering and re-extraction, particles underwent multiple rounds of heterogeneous refinement. The best-classified subset, comprising 43,625 particles, was further refined, ultimately yielding a reconstruction with an average resolution of 4.3 Å (Supplementary Fig. 12).

### Model Building

Model building for states I-IV was performed in locally refined maps. Initial models were generated by docking existing structures of p97 (PDB: 7LN5), human Npl4-Ufd1 (PDB: 7WWQ), unfolded ubiquitin (PDB: 6OA9), and AlphaFold predictions (Supplementary Fig. 8), including FAF1 AlphaFold prediction (UniProt ID: Q9UNN5, AF-Q9UNN5-2-F, Version v6) into the maps. The models were further refined, for high-resolution regions, using ISOLDE^47^ and finally refined using Phenix real space refinement^46^. For low-resolution regions and the ΔΝ FAF1 structure, each protein was rigid-body fitted to the map without any further adjustments. Structural models generated using AlphaFold3 and used for model building are provided as Supplementary Data.

Visualization and analysis were carried out using UCSF ChimeraX^48^. Visualization of the motion between individual states (Fig. 4) was done in PyMol^49^ using the python script Modevectors.

### Reporting summary

Further information on research design is available in the Nature Portfolio Reporting Summary linked to this article.

## Supplementary information

Supplementary Information

Reporting Summary

Description of Additional Supplementary Files

Dataset 1

Dataset 2

Dataset 3

Supplementary Movie 1

Transparent Peer Review file

## Source data

Source Data

## Data Availability

The hybrid cryo-EM maps of States I-IV state generated in this study have been deposited in the Electron Microscopy Data Bank (EMDB) under the following accession codes: State I EMD-58589, State II EMD-58593, State III EMD-58596 and State IV EMD-58599. The non-normalized composite maps can be found under the following accession codes: State I EMD-59986, State II EMD-59990, State III EMD-59988 and State IV EMD-59989. Corresponding maps of focused refinements on individual regions of the complex and the consensus refinements are available under accession codes: EMD-58587 (p97-State I); EMD-58588 (UN-State I); EMD- 58590 (p97-State II); EMD-58591 (UN-State II); EMD-58592 (FAF1-State II); EMD-58594 (p97-State III); EMD-58595 (UN-State III); EMD-58597 (p97-State IV); EMD-58598 (UN-State IV); EMDB-59179 [https://www.ebi.ac.uk/emdb/EMD-59179] (FAF1-State IV). The cryo-EM map of ΔN-FAF1 has been deposited under accession code EMD-58586. The cryo-EM dataset for only-C-term FAF1 has been deposited to EMPIAR under accession codes EMPIAR-13817. The atomic coordinates of the corresponding models have been deposited in the Protein Data Bank (PDB) under accession codes 31ON, 31OP, 31OQ and 31OR, for States I-IV respectively, and 31OL for ΔΝFAF1. Other data are available in the source data published alongside this manuscript. The source data underlying Fig. 3d, and Supplementary Fig. 1,2,6,7,8,9,10 are provided as a Source Data file. Source data are provided with this paper.
