# Supplementary material for: Structural dynamics of substrate processing by the p97-Ufd1-Npl4-FAF1 machinery
Source: Nat Commun. 2026 Oct 25;17:11081. doi: 10.1038/s41467-026-78639-x (PMC13696688; doi:10.1038/s41467-026-78639-x)
Supplement: Supplementary file 1 [file 41467_2026_78639_MOESM1_ESM.pdf]

Supplementary Information

Structural dynamics of substrate processing by the p97-Ufd1-Npl4-FAF1 machinery.

Athina Drakonaki[1,2], Johannes van den Boom[3], Alexander Kröning[3], Hemmo Meyer[3], Christos Gatsogiannis[1,2]*

[1]Institute of Medical Physics and Biophysics, University of Münster, Münster, Germany.

[2]Center for Soft Nanoscience (SoN), University of Münster, Münster, Germany.

[3]Molecular Biology I, Center of Medical Biotechnology, Faculty of Biology, University of Duisburg-Essen, Essen, Germany.

*Correspondence: christos.gatsogiannis@uni-muenster.de

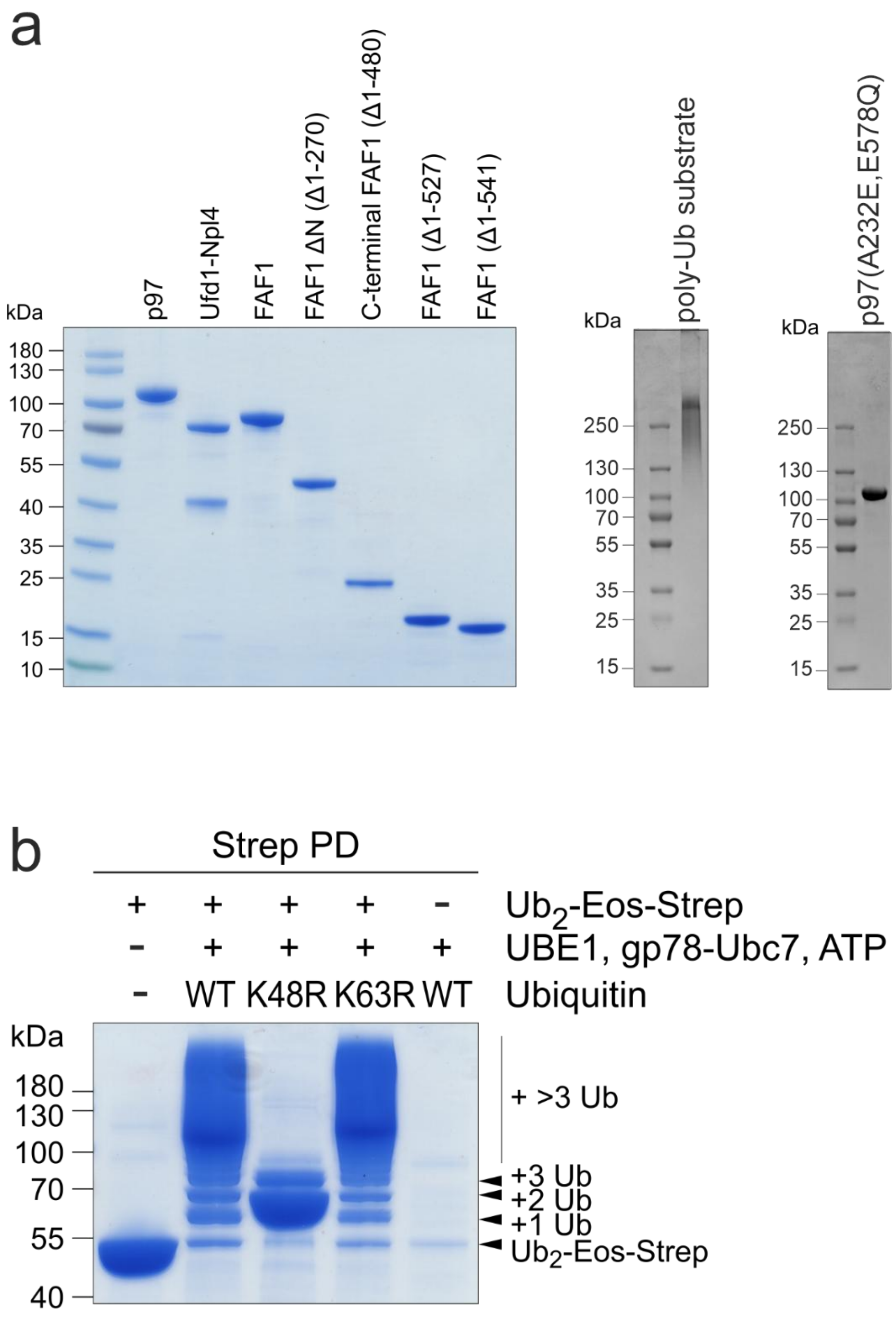

Supplementary Figure 1: Coomassie-stained SDS–PAGE gels of individual protein components and ubiquitination reactions. a) Coomassie- stained gels of individual proteins used in this study. **b)** Coomassie- stained gel of polyubiquitination reactions with ubiquitin mutants. Ub_2-Eos-Strep substrate was ubiquitinated using UBE1 (E1), gp78-Ubc2 (E2), ATP and ubiquitin variants as indicated. Unanchored chains were removed by streptactin pulldown (Strep PD).

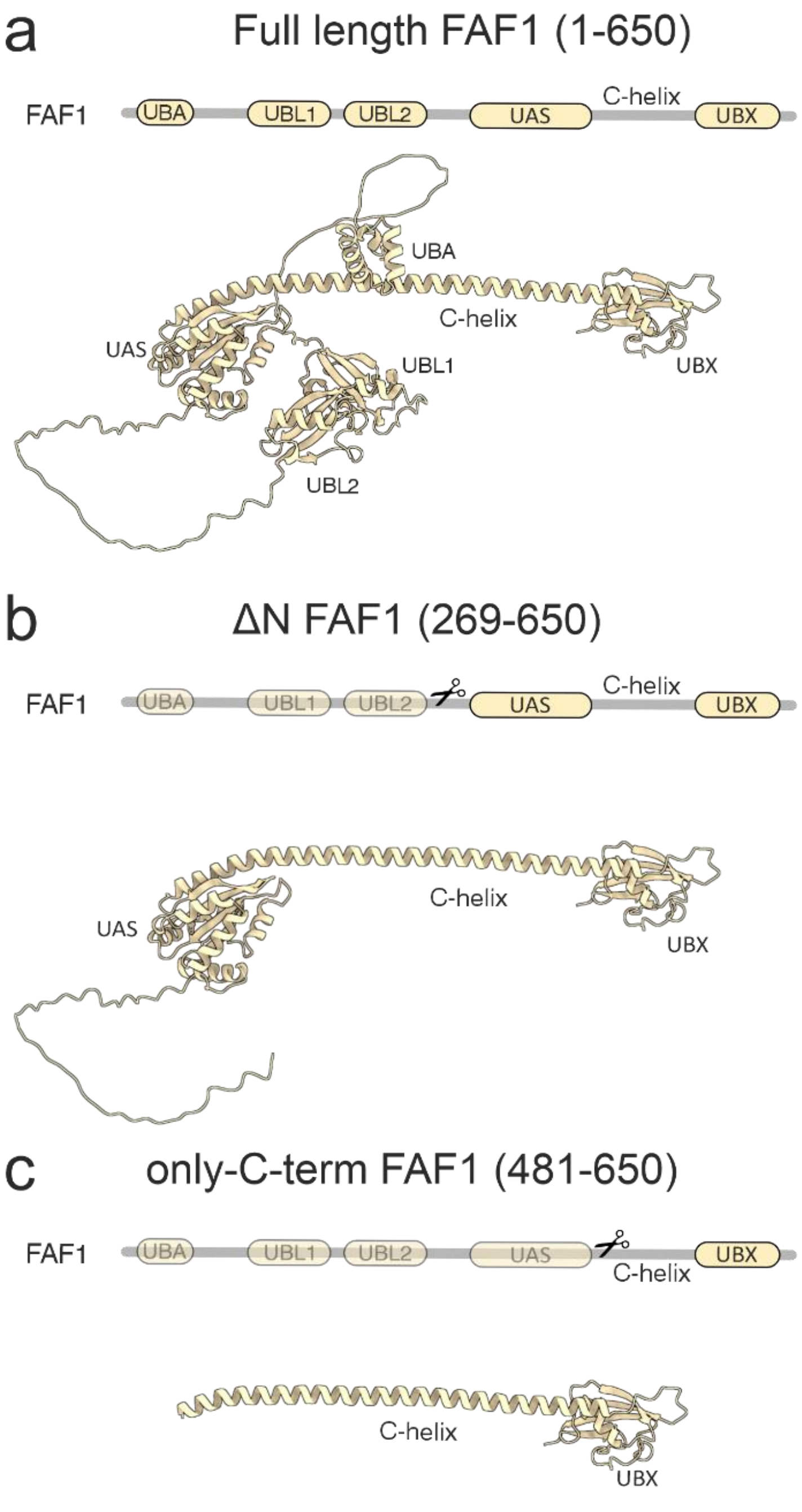

Supplementary Figure 2: AlphaFold3 predictions of full length FAF1 and truncated variants a-c) schematic representation and AlphaFold3 predicted models for full length FAF1 (1-650 incl. all domains) (a), ΔN FAF1 (269-650; truncated variant without the N-terminal UBA, UBL1 and UBL2 domains) (b) and only-C-term FAF1 (481-650, including the C-terminal helix and UBX domain) (c).

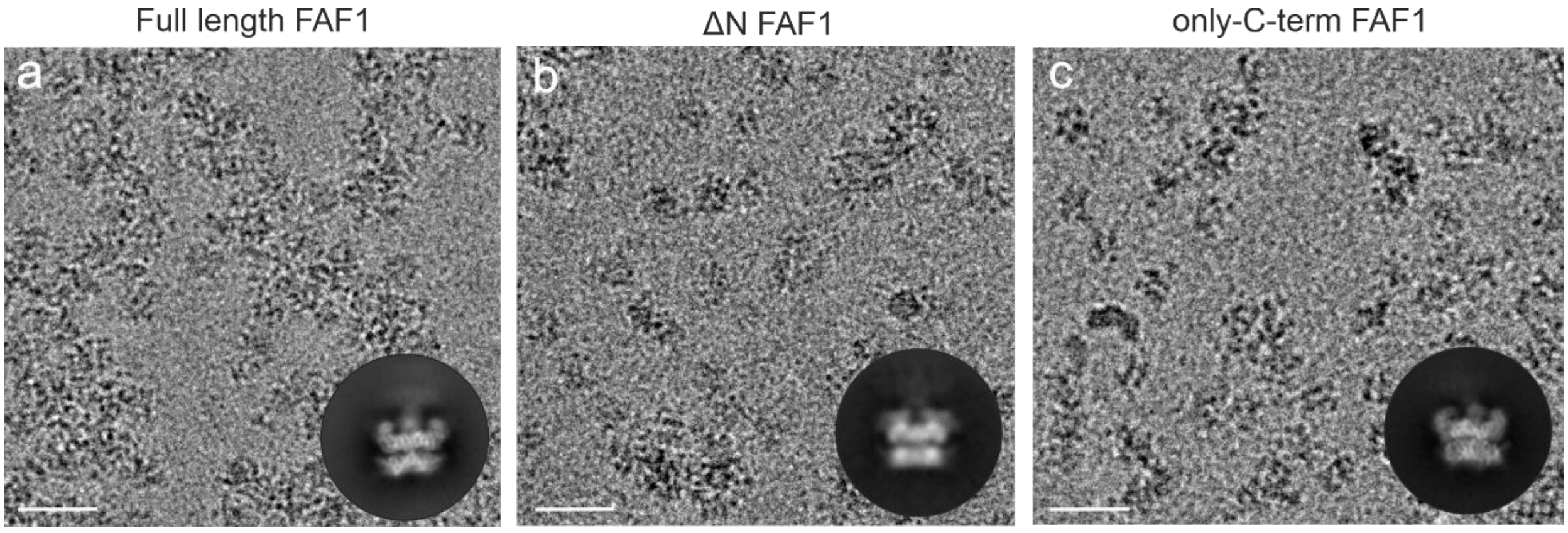

Supplementary Figure 3: CryoEM screening of substrate-engaged p97 in complex with Npl4/Ufd1 and different variants of FAF1. cryoEM micrograph and reference-free 2D class average of full length FAF1 (a), ΔN FAF1 (b) and only-C-term FAF1 (c). Scale bar: 30 nm

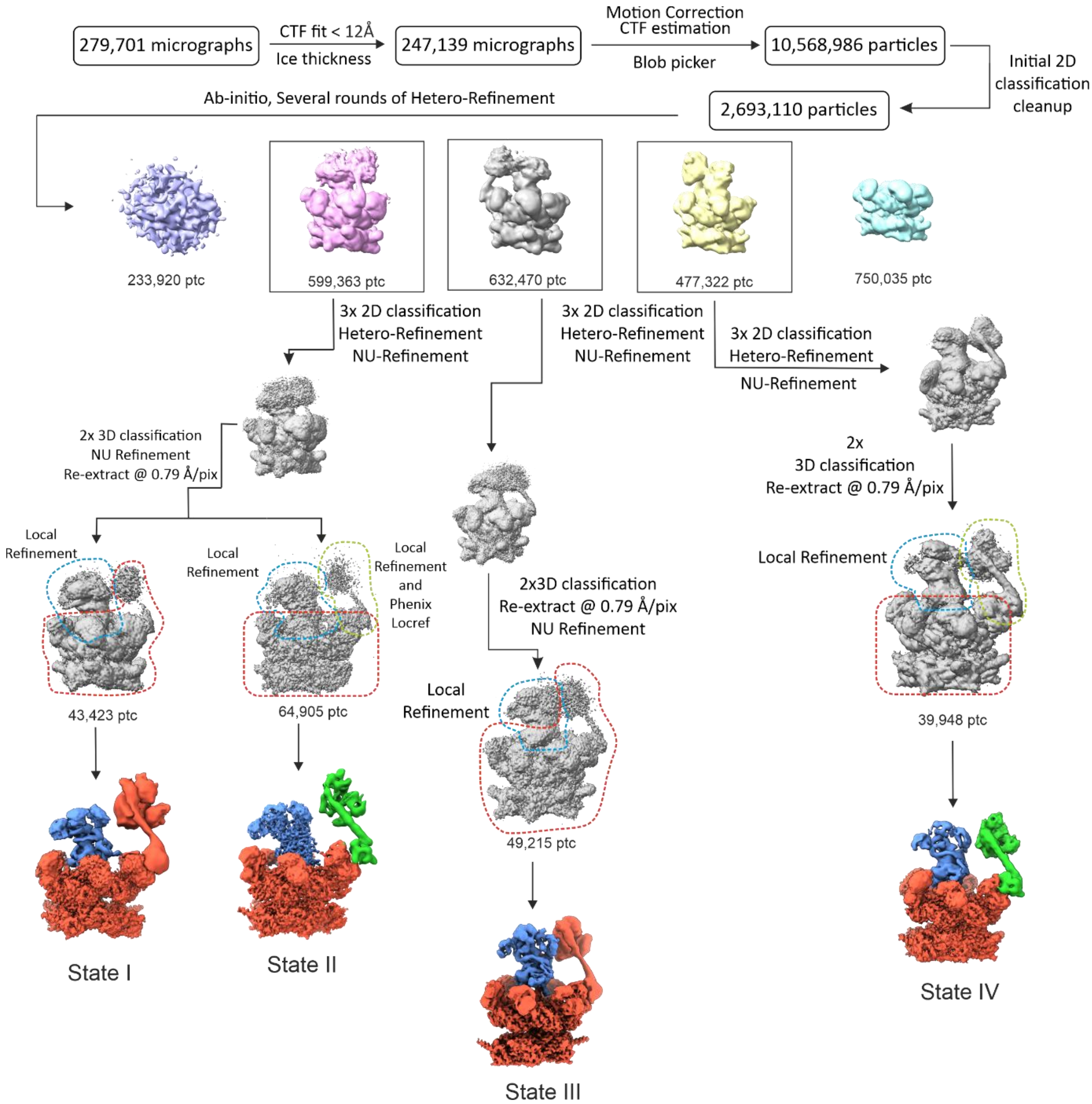

Supplementary Figure 4: Single-particle cryo-EM processing workflow of substrate-engaged p97 in complex with Npl4/Ufd1 and only-C-term FAF1. The regions indicated by dashed lines were refined individually and then merged to create composite maps for each state, with colors corresponding to the specific refined parts**.** The final composite maps of states I, II, III, IV were generated for uploading to allow visualization of the model fitting in a single volume.

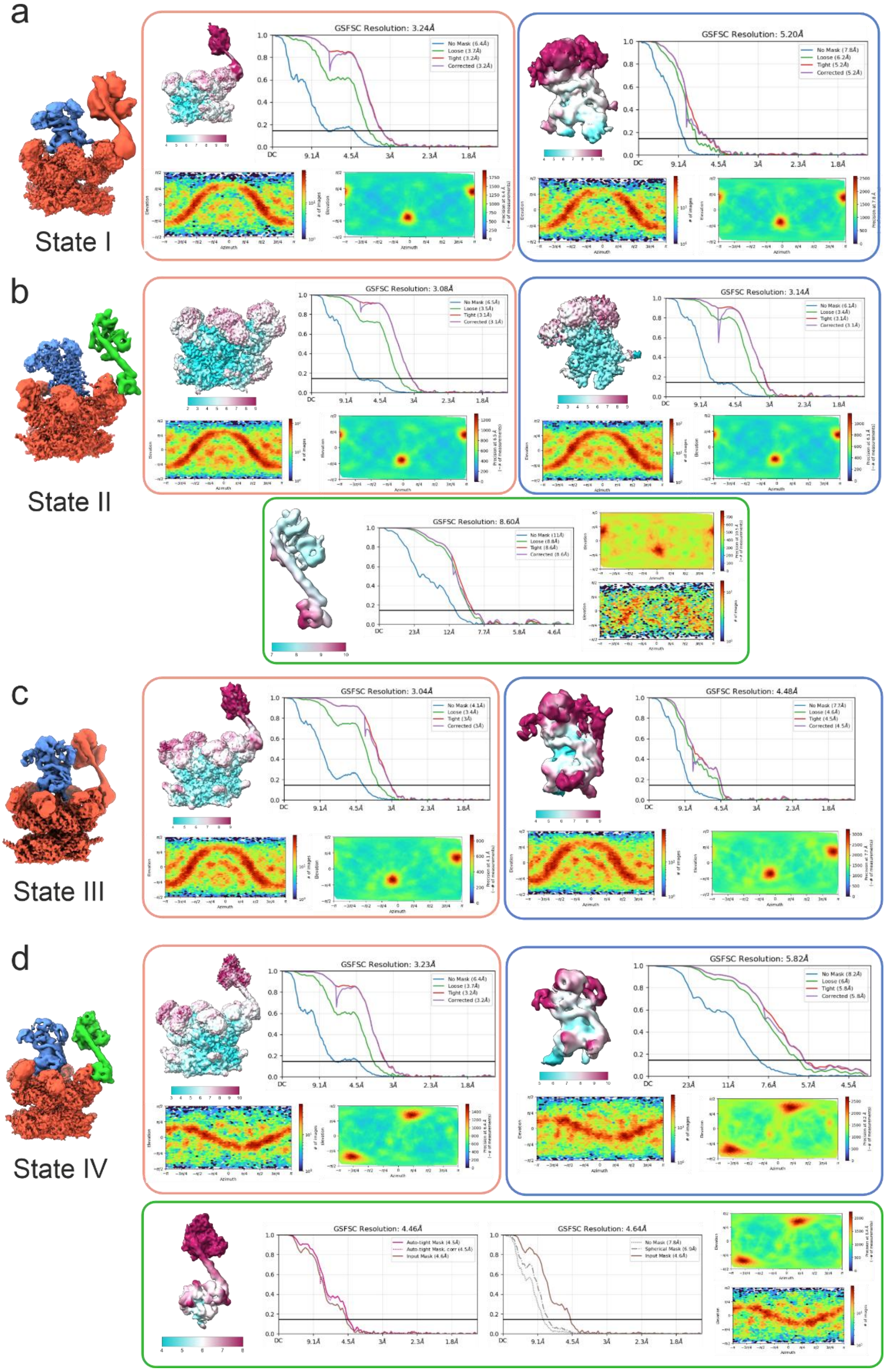

Supplementary Figure 5: CryoEM structures of states I-IV of substrate-engaged p97 in complex with Npl4/Ufd1 and only-C-term FAF1. Composite maps are shown for states I (a), II (b), III (c) and IV (d) together with the respective locally refined volumes colored according the local resolution, half-map FSC curves and angular distributions. Local refinement of the FAF1 region in state IV (lowest panel in d) was performed using cryoSPARC [Version v5.0.6], while all other refinements were conducted with cryoSPARC [Version 4.6.2]. Differences in this FSC curve labels and output format reflect differences between CryoSPARC versions. State I: p97–FAF1 consensus map (EMD-58587;

2.68σ); locally refined UN tower map (EMD-58588; 5.66σ). State II: p97 consensus map (EMD-58590; 5.79σ); locally refined UN tower map (EMD-58591; 4.28σ); locally refined FAF1 map (EMD-58592; 14.07σ). State III: p97–FAF1 consensus map (EMD-58594; 3.83σ); locally refined UN tower map (EMD-58595; 6.17σ). State IV: p97–FAF1 consensus map (EMD-58597; 5.67σ); locally refined UN tower map (EMD-58598; 6.27σ); locally refined FAF1 map (EMD-59179; 6.57σ).

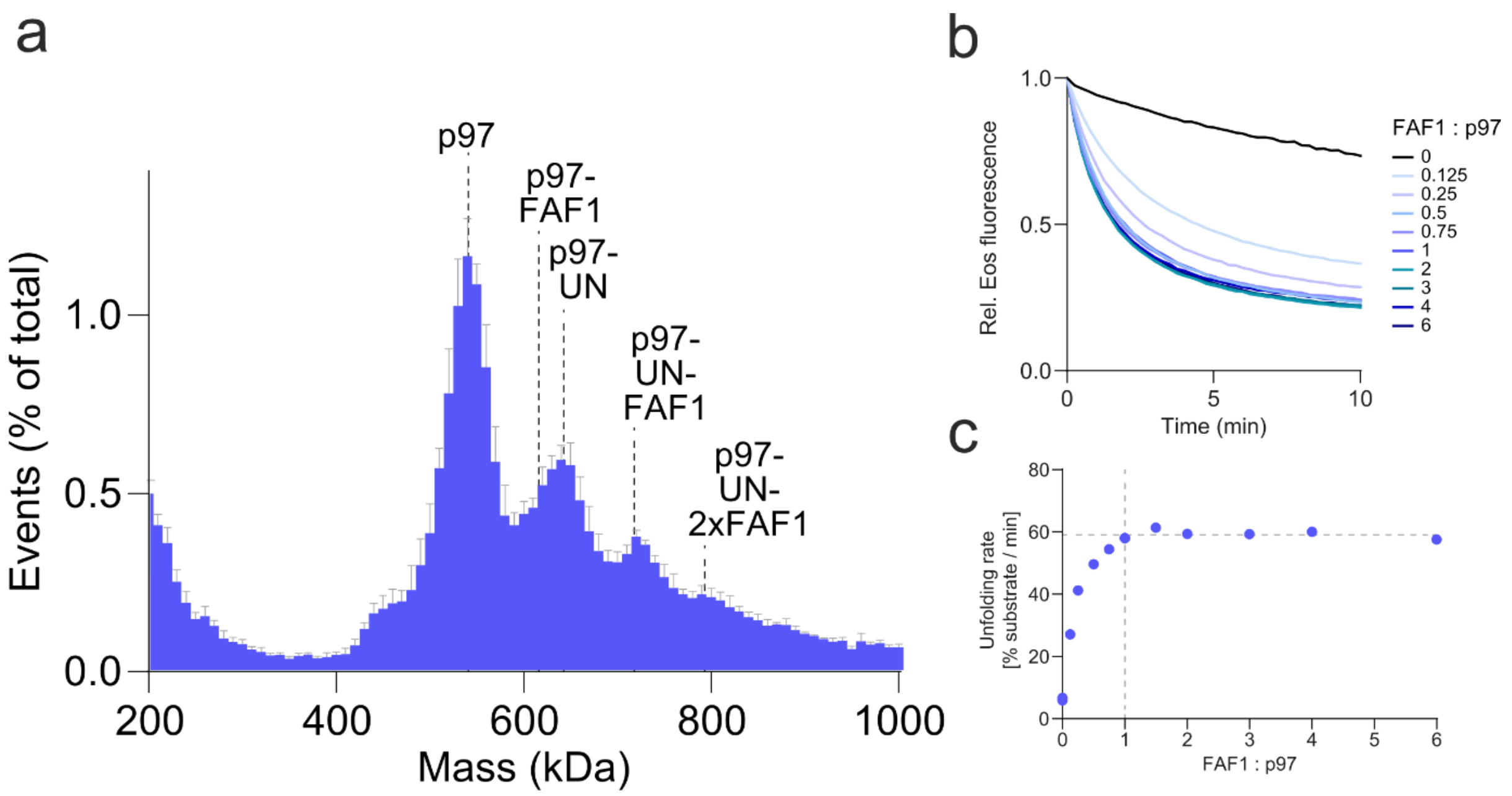

Supplementary Figure 6: Mass photometry and substrate unfolding activity of p97–Ufd1–Npl4–FAF1 assemblies. a) Mass distribution of the p97-Ufd1-Npl4-FAF1 complex crosslinked with DSSO as determined by mass photometry. Expected masses of p97 (541 kDa), p97-FAF1 (616 kDa), p97-Ufd1-Npl4 (643 kDa), p97-Ufd1-Npl4-FAF1 (718 kDa) are indicated. At the expected mass for the complex with 2xFAF1 (793 kDa), no major peak is detected. N=3 ± SD. **b)** p97 substrate unfolding activity with different ratios of FAF1: p97. p97 (500 nM), Ufd1-Npl4 (1500 nM) and FAF1 were incubated for 5 min before Eos fluorescence was recorded upon addition of ATP at t=0. **c)** Quantification of (b). Initial unfolding rates were quantified by nonlinear regression. Dashed lines indicate that maximum velocity is reached at a stoichiometry of 1 FAF1 per p97 complex.

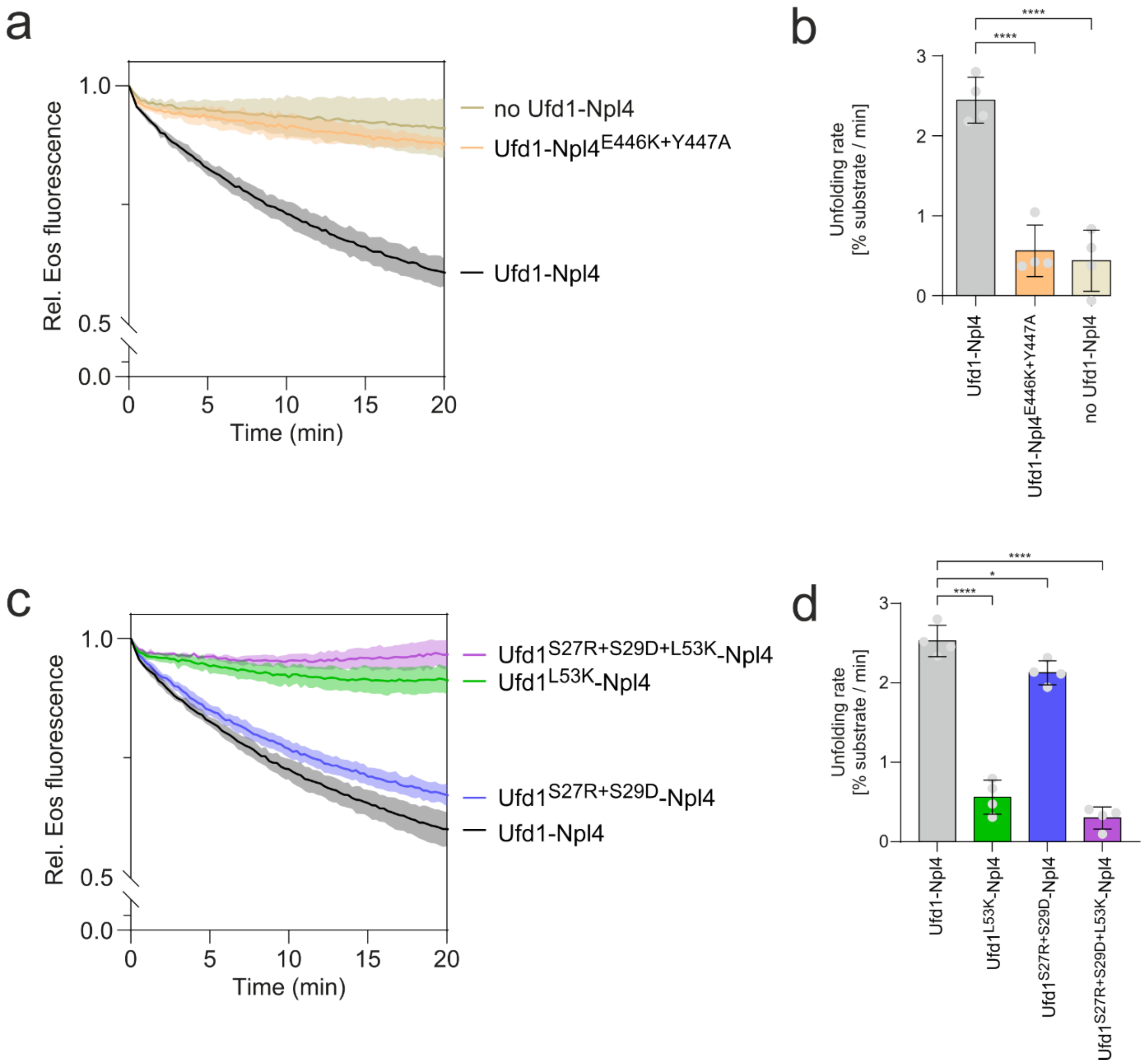

Supplementary Figure 7: Functional analysis of Npl4 and Ufd1 variants in p97-driven substrate unfolding. a) p97 substrate unfolding activity assay with Npl4 mutant. Normalized Eos fluorescence was recorded upon addition of ATP at t=0. Means ± SD; n = 4. **b)** Quantification of (a). Initial unfolding rates were quantified by linear regression. Means ± SD; n = 4. Significance was tested with ANOVA and Tukeys‘s multiple comparison test. **** = p<0.0001. **c)** p97 substrate unfolding activity assay as in (a) with Ufd1 variants. Means ± SD; n = 4. **d)** Quantification of (c). Initial unfolding rates were quantified by linear regression. Means ± SD; n = 4. Significance was tested with ANOVA and Tukeys‘s multiple comparison test. **** = p<0.05.

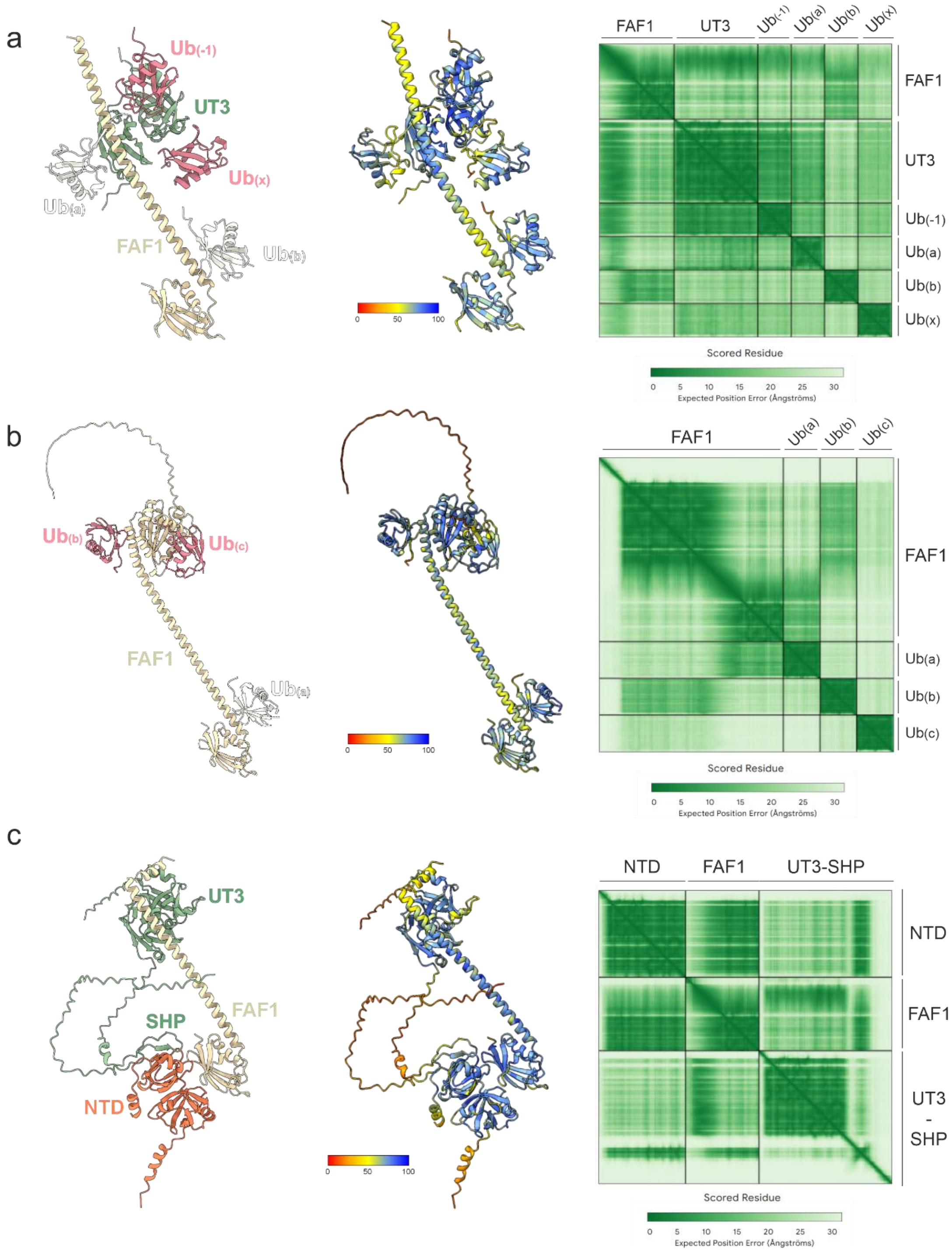

Supplementary Figure 8: AlphaFold3 predicted models used in this study for rigid body fitting into cryoEM volumes. a) Predicted AlphaFold3 model of only-C-termFAF1 (beige) in complex with the UT3 domain of Ufd1 (green) and four ubiquitin molecules (pink and transparent). The model was rigid-body fitted into the cryoEM density without further adjustments, but the two ubiquitin molecules (transparent) that lacked any density were omitted. **b)** Predicted AlphaFold3 of model ΔN FAF1 (beige) and three ubiquitins (pink and transparent). the model was rigid-body fitted into the cryoEM density without further adjustments, but one ubiquitin molecule (transparent) that lacked any density was omitted. **c)** Predicted model of only-C-term FAF1 (beige), Ufd1 (green) and N terminal of p97 (orange). After docking into the high-resolution density map, the model was further refined, omitting disordered regions that lacked clear density. The predicted error estimates (PAE) for the predictions are shown in shades of green.

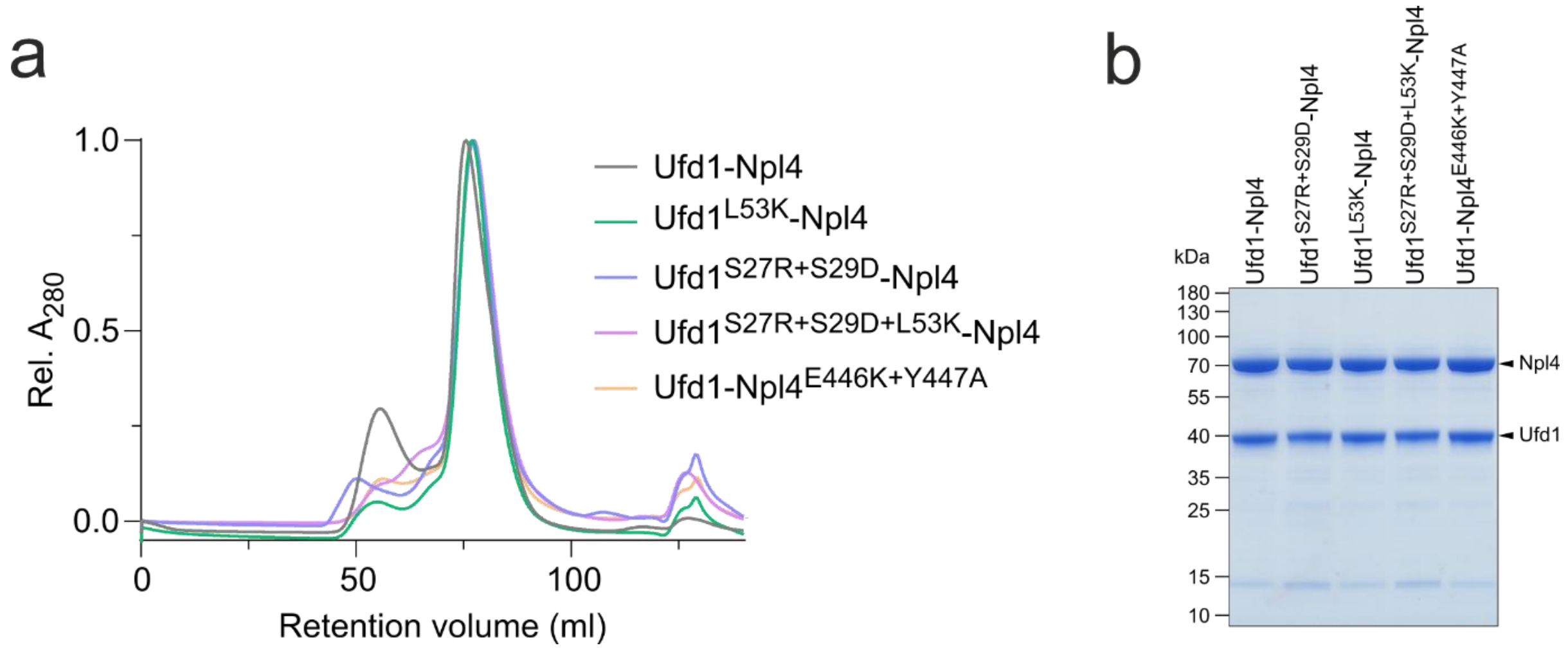

Supplementary Figure 9: Biochemical characterization of Ufd1–Npl4 variants. a) Preparative gel filtration chromatograms of Ufd1-Npl4 variants on a Superdex 200 16/600 column. No signs of increased aggregation or impaired hetero-dimerization was observed for the mutants. **b)** Coomassie-stained gel of purified Ufd1-Npl4 variants. Note that equal amounts of untagged Npl4 are co- purified with the His- tagged Ufd1 mutants.

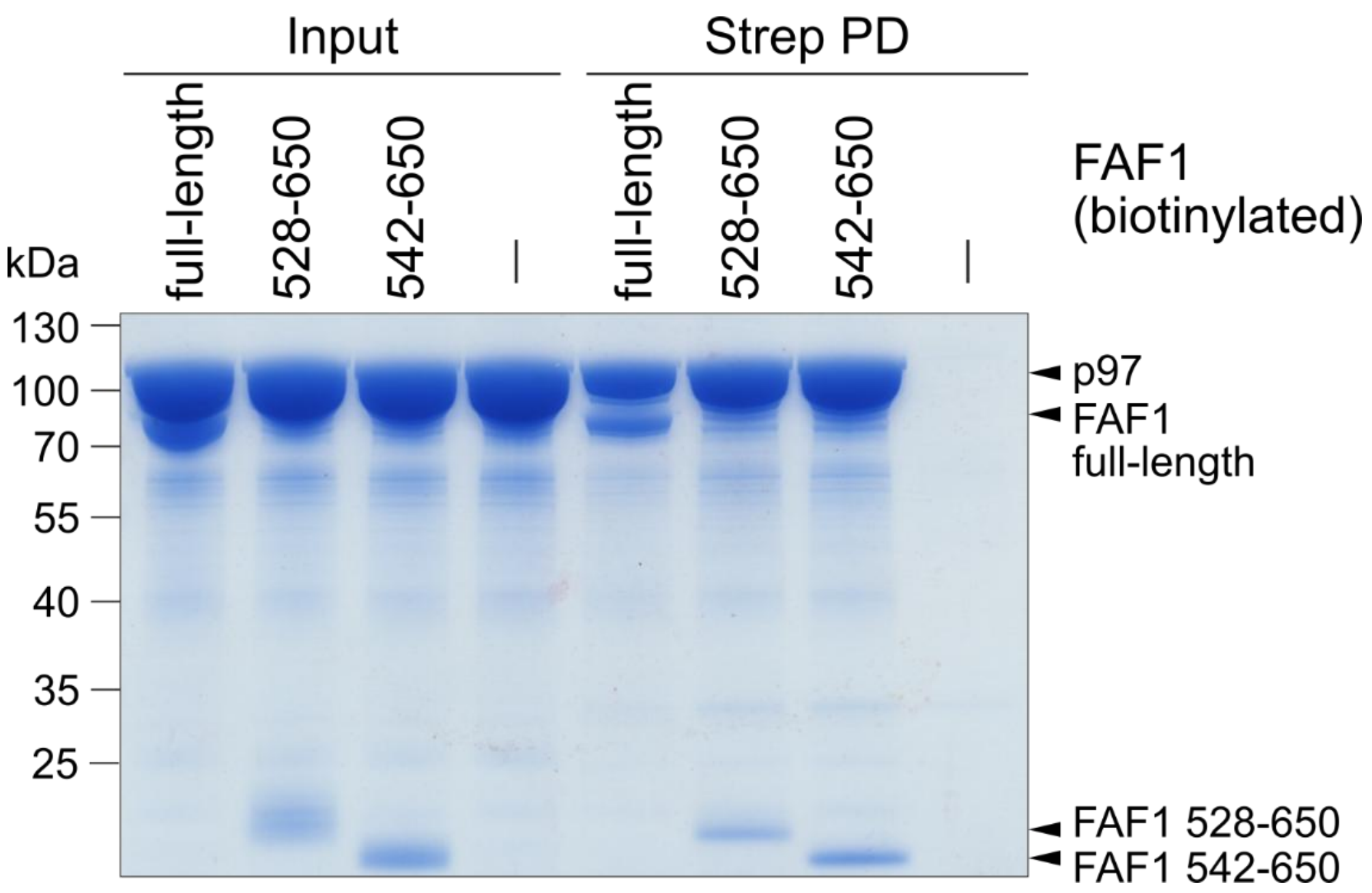

Supplementary Figure 10: FAF1 truncations are not impaired in p97 binding. p97 was incubated with biotinylated full-length or truncated FAF1, as indicated. Coomassie- stained gel shows co- eluted p97 after streptavidin pulldown (Strep PD).

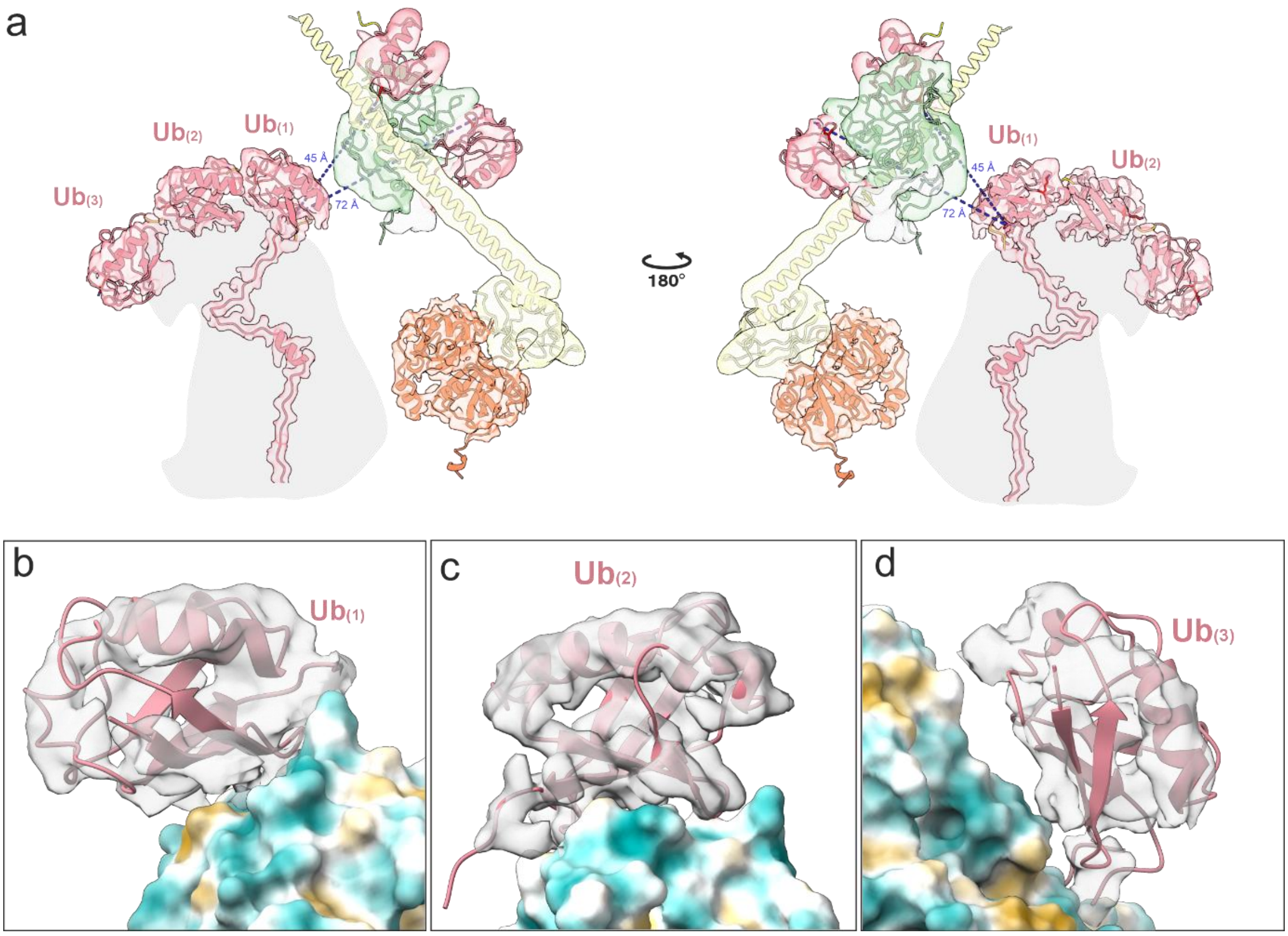

Supplementary Figure 11: Distal ubiquitins capping the Npl4 tower. a) Side view of the cryoEM volume and fitted models of stat II with Npl4 and p97 removed (except the NTD interacting with FAF1) (see Figure 3a). Dashed lines indicate the distance of the C-term of the initiator ubiquitin to the branchpoints of proximal to the substrate ubiquitins Ub(-1) and Ub(x) bound to FAF1 and UT3. **b-d)** View of the distal ubiquitins Ub(1) (b), Ub(2) (c) and Ub(3) (d) on the top of Npl4. Ubiquitins are fitted in their corresponding density colored in gray. Npl4 is represented as surface and colored by hydrophobicity. The State II consensus map (EMD-58590) is complemented by locally refined maps showing the unUb and distal ubiquitins (EMD-58591) and the FAF1–UT3–proximal ubiquitin region (EMD-58592). Density is contoured at 15.7σ for FAF1–UT3–proximal ubiquitin region, 6.2σ for UN tower, and 6.6σ for p97. The distal ubiquitins (Ub1–Ub3) are contoured at 9.24σ, 9.30σ, and 7.07σ, respectively.

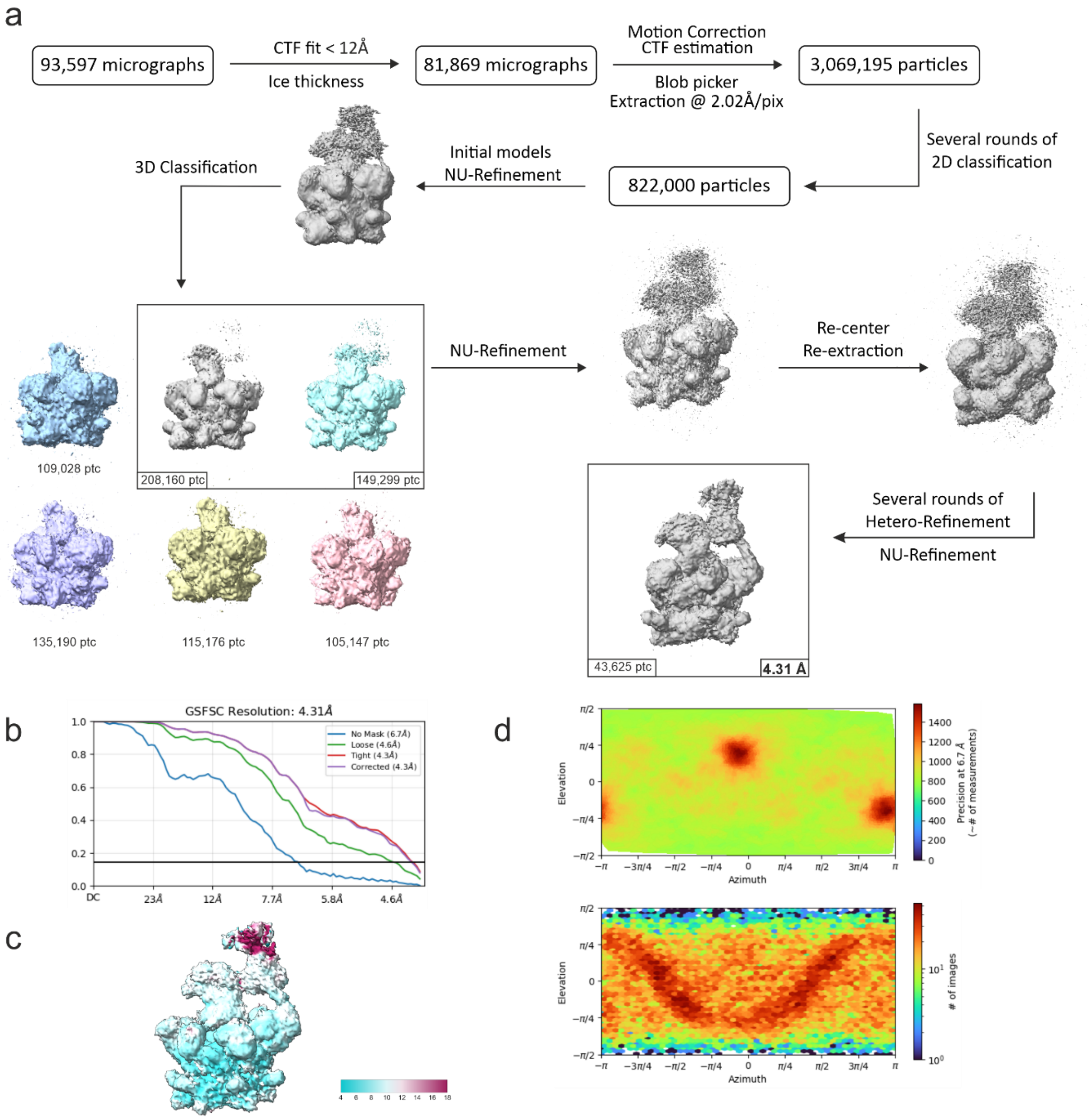

Supplementary Figure 12: Single-particle cryo-EM processing workflow of substrate-engaged p97 in complex with Npl4/Ufd1 and ΔN-FAF1. a) Flowchart for cryo-EM data processing. **b)** Gold standard FSC **c)** Final cryoEM volume colored according to the local resolution (EMDB-58586). **d)** Euler angle distribution. Density is contoured at 1.92σ.

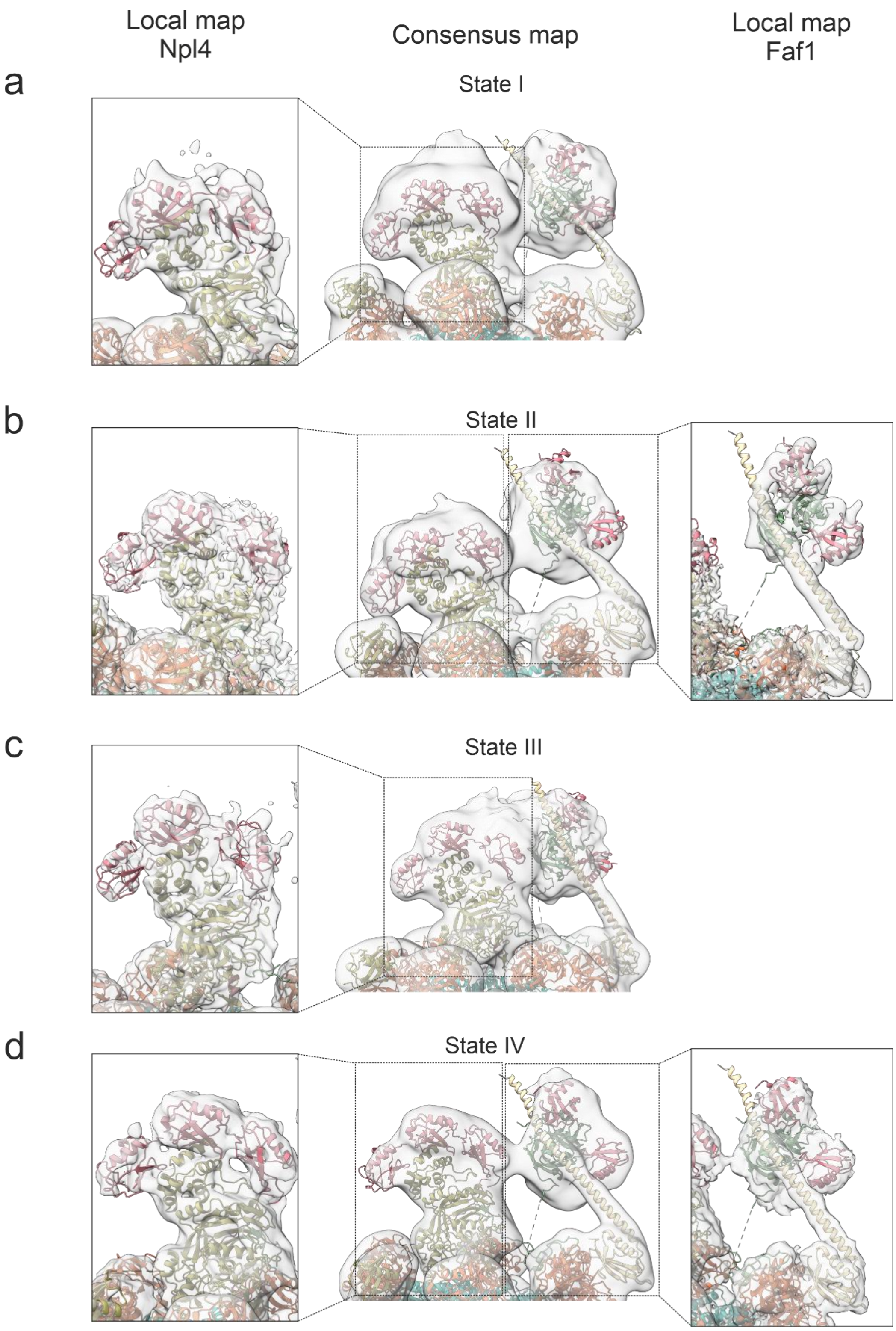

Supplementary Figure 13. Consensus and locally refined maps of the Npl4 and FAF1/UT3–proximal ubiquitin regions across States I–IV. The four states are shown in (a–d), with State I in (a), State II in (b), State III in (c), and State IV in (d). Unmasked filtered consensus maps are shown in the middle column with the corresponding fitted models. Where available, locally refined maps are shown in the left and right columns. The left column shows the locally refined Npl4 region with the corresponding fitted model (for states I, II, III and IV), while the right column shows the locally refined FAF1/UT3–proximal ubiquitin region with the corresponding fitted models (for states II and IV). State I: p97–FAF1 consensus map (EMD-58587; 1.54σ); locally refined UN tower map (EMD-58588;

4.64σ). State II: p97 consensus map (EMD-58590; 2.51σ); locally refined UN tower map (EMD-58591; 5.14σ); locally refined FAF1 map (EMD-58592; 14.1σ). State III: p97–FAF1 consensus map (EMD-58594; 1.69σ); locally refined UN tower map (EMD-58595; 6.12σ). State IV: p97–FAF1 consensus map (EMD-58597; 4.06σ); locally refined UN tower map (EMD-58598; 5.72σ); locally refined FAF1 map (EMD-59179; 6.07σ). Consensus maps for p97 are filtered in this figure.

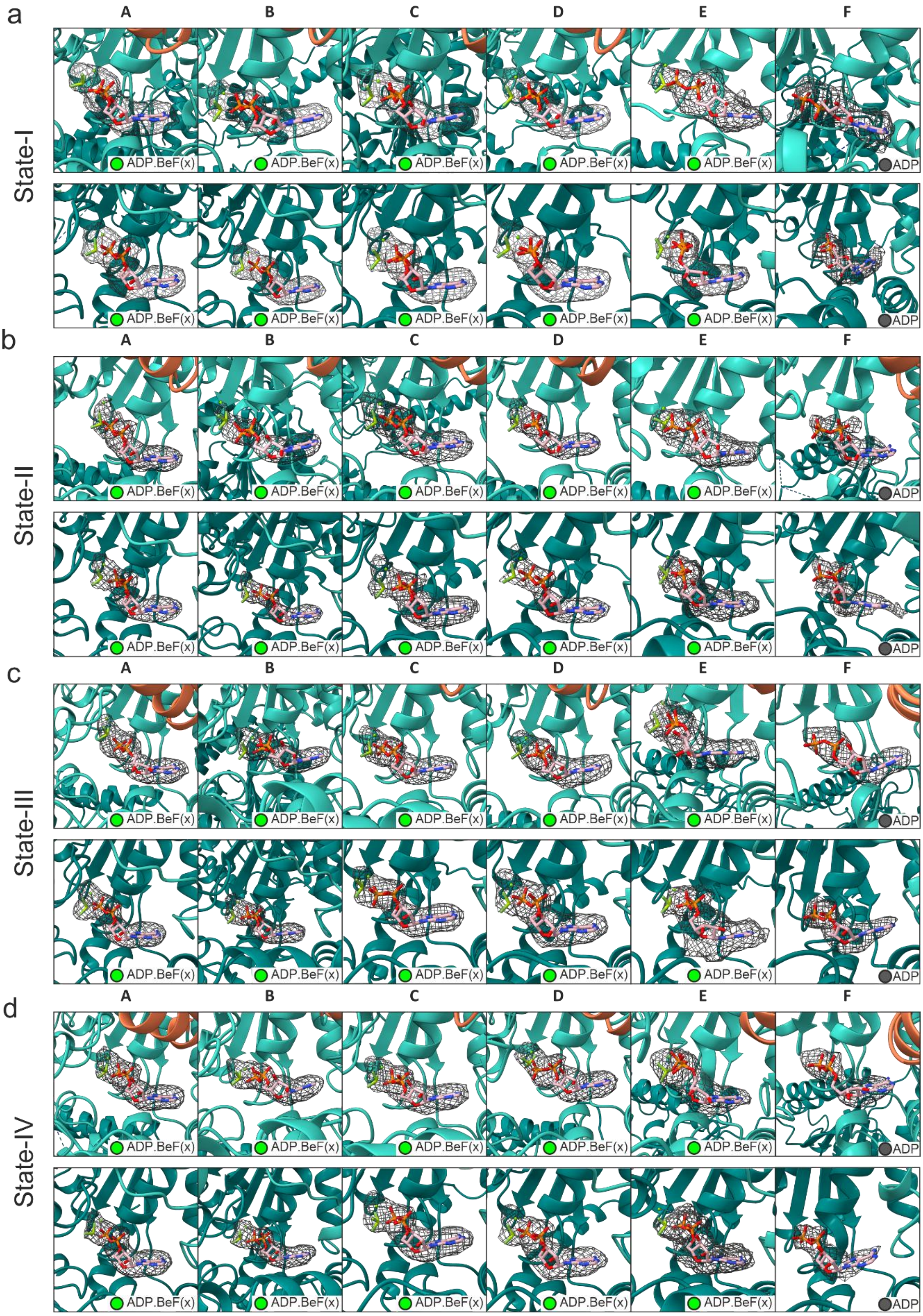

Supplementary Figure 14: Analysis of nucleotide binding pockets. Nucleotide models and corresponding cryo-EM density of the D1 (upper panels) and D2 (lower panels) ring of State I (a), State-II (b), State-III (c) and State-IV (d). State I: p97–FAF1 consensus map (EMD-58587; 8.94σ), State II: p97 consensus map (EMD-58590; 6.84σ), State III: p97–FAF1 consensus map (EMD-58594; 7.84σ), State IV: p97–FAF1 consensus map (EMD-58597; 7.57σ).

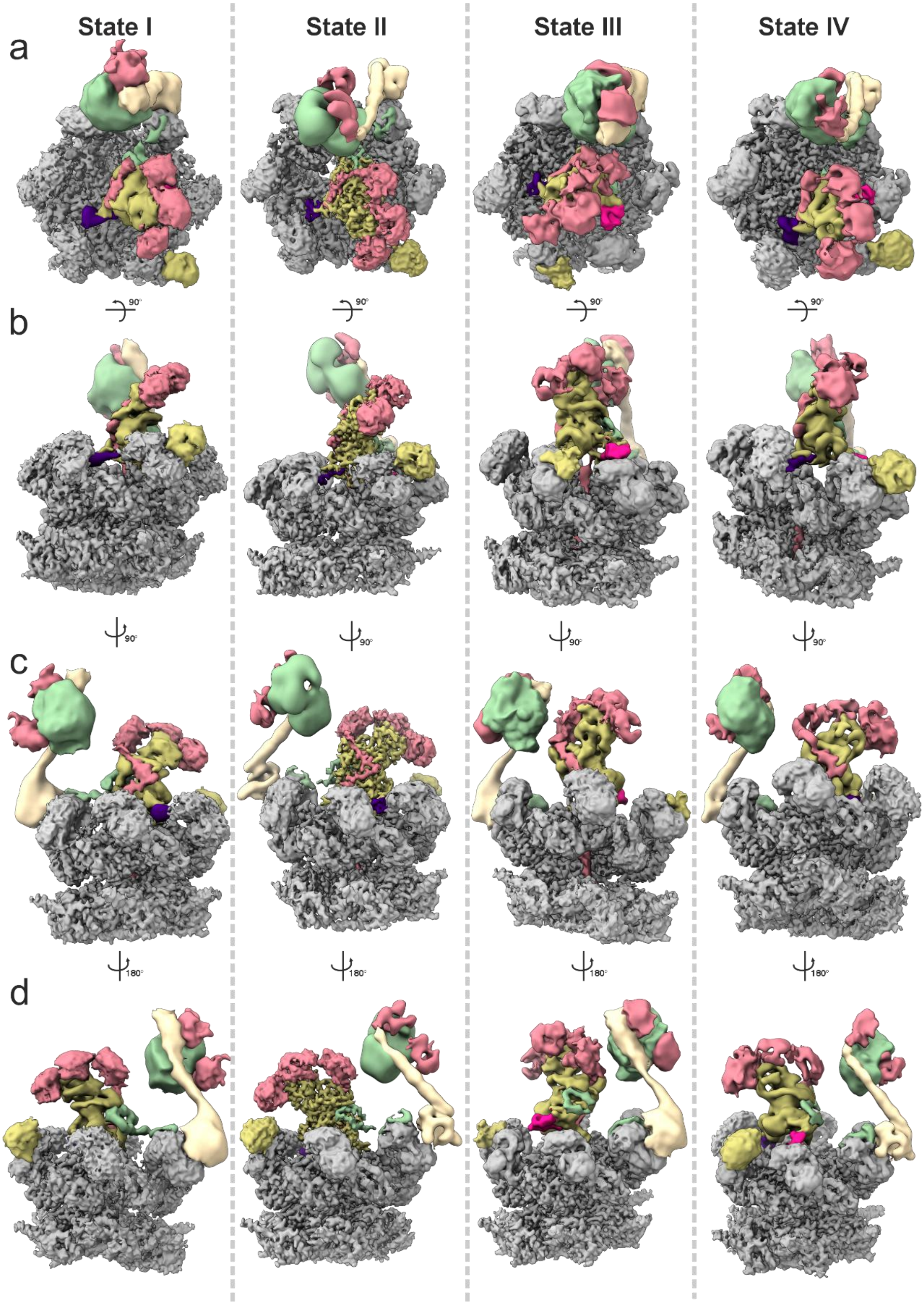

Supplementary Figure 15: Four states of substrate engaged p97-Ufd1-Npl4-FAF1. a-d) Different views of cryo-EM volumes of states I-IV (see also Figure 4). p97: gray, Npl4: khaki (ZF1: magenta, ZF2: pink), Ufd1: green, FAF1: beige, ubiquitins: pink. State I: p97–FAF1 consensus map (EMD-58587; 6.37σ); locally refined UN tower map (EMD-58588; 6.7σ). State II: p97 consensus map (EMD-58590; 7.8σ); locally refined UN tower map (EMD-58591; 6.2σ); locally refined FAF1 map (EMD-58592; 15.7σ). State III: p97–FAF1 consensus map (EMD-58594; 4.05σ); locally refined UN tower map (EMD-58595; 5.27σ). State IV: p97–FAF1 consensus map (EMD-58597; 5.13σ); locally refined UN tower map (EMD-58598; 4.68σ).

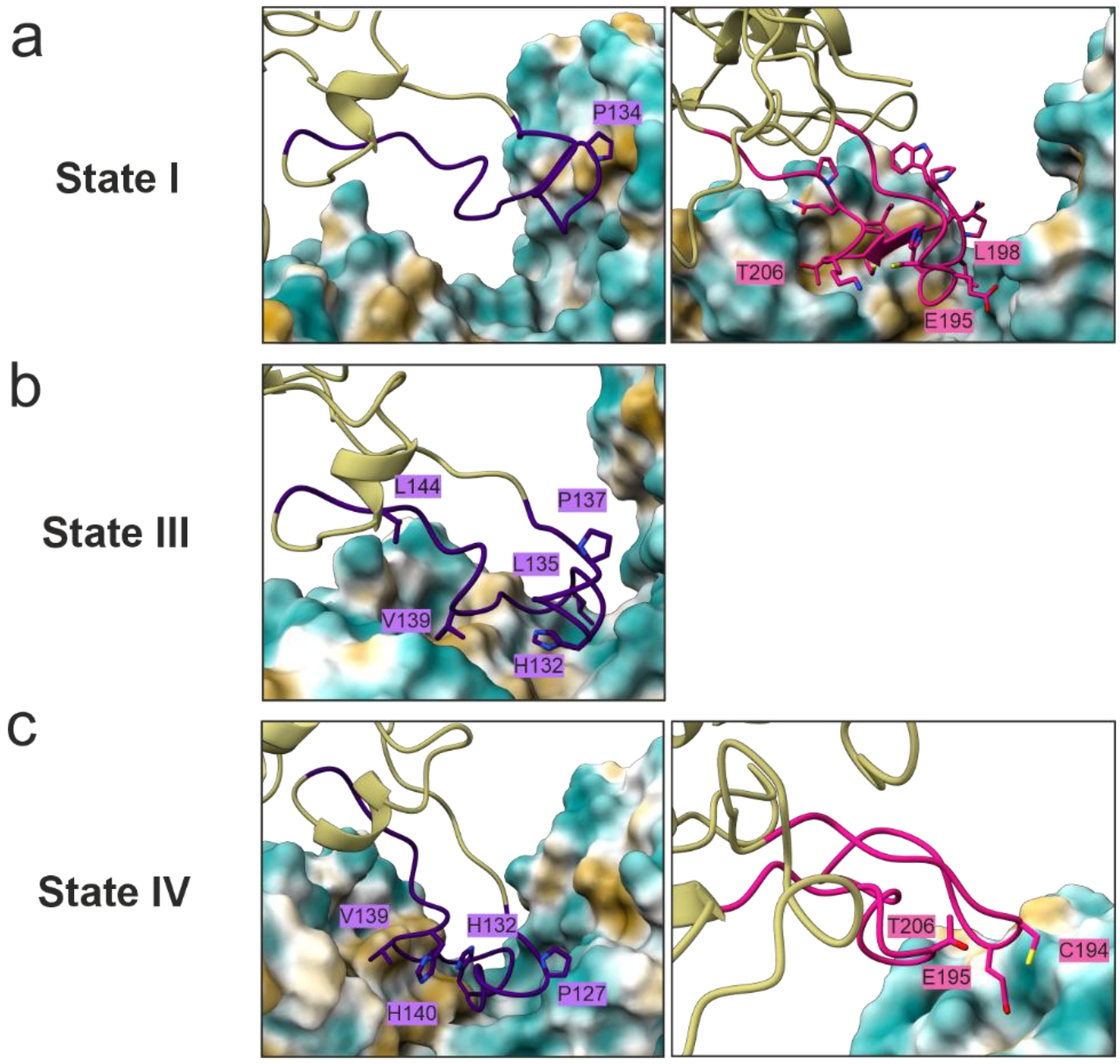

Supplementary Figure 16: Interfaces between ZF1 and ZF2 of Npl4 and p97 in states I, III and IV. a) State I: Interactions of ZF2 (magenta) and ZF1 (pink) with the D1 subunit A and the NTD of subunit C, respectively. **b)** State III: Interactions of ZF2 (magenta) with a groove between the NTD and D1 of the seam subunit. **c)** State IV: Interactions of ZF2 (magenta) with a groove between the NTD and D1 of the seam subunit and ZF1 with the NTD of subunit D. The respective regions of p97 involved in the interfaces are represented as surface and colored by hydrophobicity. Interfaces of ZFs in State II are shown in Figure 2e,f. Side-chains are not resolved by the density and are shown only to indicate the regions involved, as the local resolution of Npl4 is 5.0 (State I), 4.48 (State III), and 5.82 Å (State IV) in the respective maps.

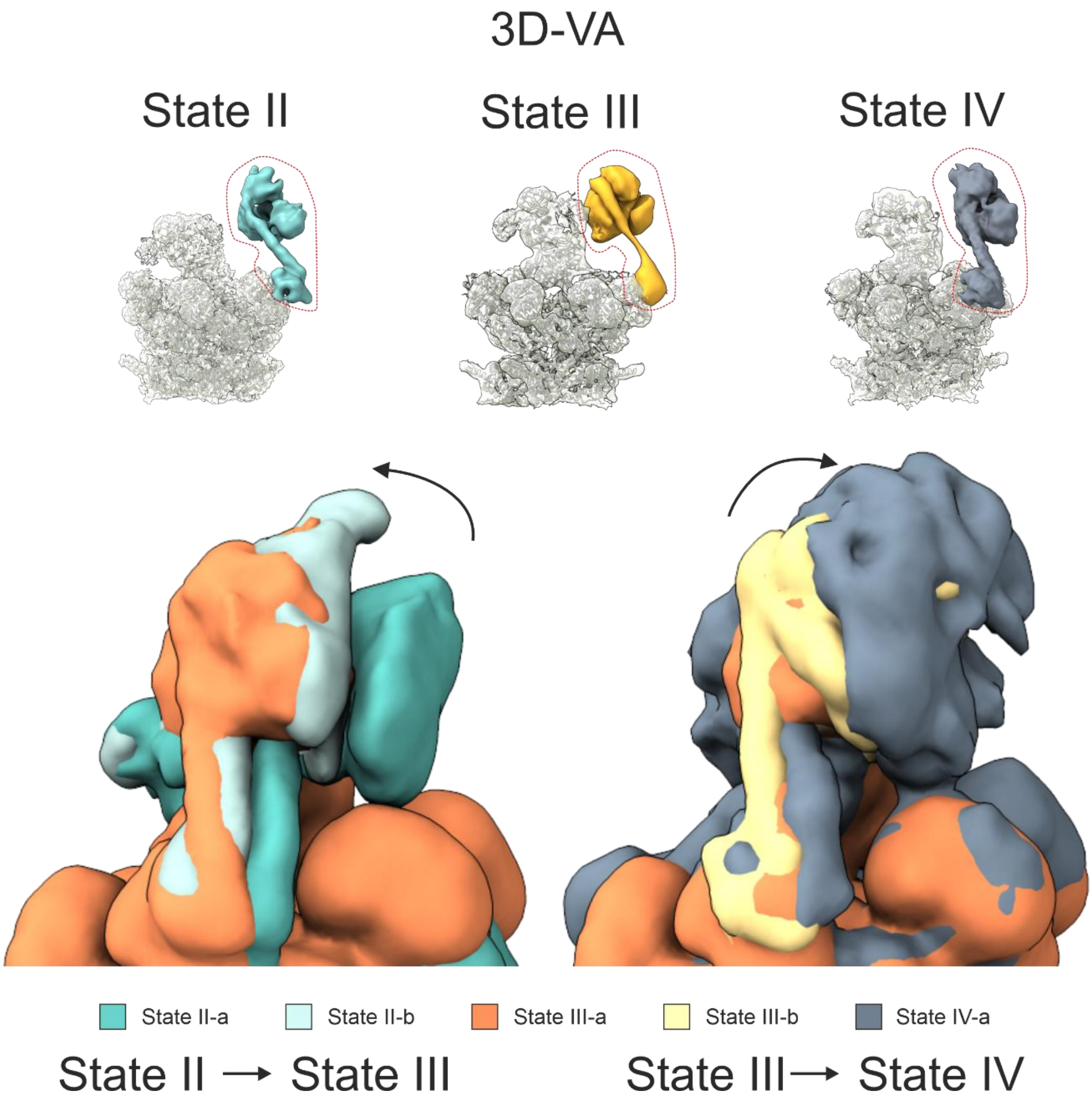

Supplementary Figure 17. 3D Variability (3D-VA) analysis of state II, III and IV focusing on FAF1, UT3 and proximal ubiquitins. The analysis reveals a continuous conformational trajectory involving FAF1, the Ufd1-UT3 domain, and the proximal ubiquitins. In particular, FAF1 undergoes a continuous tilting motion across the different states, supporting a continuous local conformational trajectory, while Npl4 and p97 retain distinct state-specific conformations. We show the two extreme states of 3D VA of the dataset of State-II (State-II-a; teal, State-II-b; cyan). We superposed them with the first extreme frame of State-III. Note that the two substates of State-II agree with a continuous movement (e.g. outward tilt of FAF1) towards State-III, consistent with the proposing sequential model. Similarly, two extreme frames of 3D VA of State-III (State-III-a; orange, State-III-b; yellow) and first extreme frame of State-IV (State-IV-a; gray) are shown (e.g. inward tilt of FAF1). Because 3D VA was conducted on separate, already classified subsets, the analysis captures only the residual heterogeneity present within each classified state.

Supplementary Table 1: *In vitro* reconstitution of only-C-term, ΔN-FAF1 and full length FAF1 complexes.

**Protein**	**Molar Ratio**
p97 (A232E, E578Q)	5
Ufd1/Npl4	1
$(Ub)^n$-m.Eos.3.2	1
Only-C-term FAF1 or ΔN FAF1 or full length FAF1	1

Supplementary Table 2: Cryo-EM data collection, refinement and validation statistics.

	Only-C-term FAF1				ΔN FAF1
**Data collection and processing**					
**Magnification**	270,000				270,000
**Voltage (kV)**	300				300
**Electron exposure (e⁻/Å²)**	50				50
**Defocus range (μm)**	-0.8 to -2.6				-0.8 to -2.6
**Pixel size (Å)**	0.46				0.46
**Symmetry imposed**	C1				C1
**Initial movies (no.)**	279,701				93,597
**Final movies (no.)**	247,139				81,869
**Initial particle images (no.)**	10,568,986				3,069,195
	**State-I**	**State-II**	**State-III**	**State-IV**	
**Final particle images (no.)**	43,423	64,905	49,215	39,948	43,625
**Map resolution (Å)**	p97 3.2<br>Npl4 5<br>FAF1 12	p97 3.08,<br>Npl4 3.14,<br>FAF1 8.6	p97 3.04<br>Npl4 4.48<br>FAF1 12	p97 3.23<br>Npl4 5.82<br>FAF1 12	4.31
**FSC threshold**	0.143				0.143
**Map resolution range (Å)**	3.2-12	3-10	3-12	3.2-12	4-18
**Refinement**					
**Model resolution (Å)**<br>**FSC threshold**	3.5<br>0.143	3.6<br>0.143	4.1<br>0.143	3.4<br>0.143	
**Model composition**<br>**Non-hydrogen atoms**<br>**Protein residues**<br>**Ligands**	46,163<br>5,821<br>ADP: 12<br>MG: 10<br>BEF: 10	46,212<br>5,833<br>ADP: 12<br>MG: 10<br>BEF: 10	46,259<br>5,839<br>ADP: 12<br>MG: 10<br>BEF: 10	46,066<br>5,811<br>ADP: 12<br>MG: 10<br>BEF: 10	
***B* factors (Å²)**<br>**Protein (min/max/mean)**	12.3/366.1/161.3	214.4/935.2/471.4	78.0/1067.5/439.4	127.8/622.7/293.2	
**Ligand (min/max/mean)**	84.2/262.4/137.8	397.1/530.4/462.4	258.1/705.2/458.3	163.9/391.5/263.2	
**R.m.s. deviations**<br>**Bond lengths (Å)**<br>**Bond angles (°)**	0.032<br>2.457	0.016<br>1.097	0.016<br>1.444	0.017<br>1.539	
**Validation**<br>**MolProbity score**<br>**Clashscore**<br>**Poor rotamers (%)**	1.45<br>2<br>1.2	1.85<br>10<br>0.2	1.82<br>9<br>0.28	1.61<br>5<br>0.68	
**Ramachandran plot**<br>**Favored (%)**<br>**Allowed (%)**<br>**Disallowed (%)**	93.75<br>6.21<br>0.03	95.31<br>4.69<br>0	95.14<br>4.84<br>0.02	95,78<br>4.17<br>0.05	

Supplementary Table 3: Plasmids used in this study.

Plasmid	Source and Reference
pET15 His-p97	(van den Boom *et. al* 2016)[1]
pFL His-p97	Weith *et al.* 2018[2]
pET41b+ Ufd1-TwinStrep	This study
pET41b+ Npl4	(van den Boom *et. al* 2016)[1]
pET28a His-diUbv-mEos3.2-TwinStrep	This study
pET28a His-diUbv-mEos3.2	Adapted from Blythe *et al*, 2017[3]
pET28 His-FAF1 Helix-UBX (481-650)	Kracht *et al.* 2026 [4]
pET28 His-FAF1 ΔN (Δ1-270)	Kracht *et al.* 2026[4]
pET28 His-FAF1	Kracht *et al.* 2026[4]
pET15 His-p97 E578Q A232E	This study
pET28 His-FAF1 528-650	This study
pET28 His-FAF1 542-650	This study
pET41 Ufd1-His S27R+S29D	This study
pET41 Ufd1-His S27R+S29D+L53K	This study
pET41 Ufd1-His L53K	This study; similarly described here [5]
pET41 Npl4 E446K+Y447A	This study; similarly described here [6]
pET23 Ubiquitin	Meyer *et al.* 2002[7]
pET23 Ubiquitin K48R	Wang *et. al* 2004 [8]
pET23 Ubiquitin K63R	Wang *et. al* 2004[8]

Supplementary Table 4: Cryo-EM maps, conformational states, EMDB accession entries and contour level for Main and Supplementary Figure panels.

**Figure**	**State**	**Panel**	**Map**	**EMDB ID**	**Contour level**
Figure 1	State II	b, c	Consensus map (p97)	58590	7.8σ
			Local Refinement (Npl4)	58591	6.2σ
			Local Refinement (FAF1)	58592	15.7σ
		d	Consensus map (p97)	58590	6.6σ
			Local Refinement (Npl4)	58591	6.4 σ
Figure 2	State II	Overview	Consensus map (p97)	58590	6.6σ
			Local Refinement (Npl4)	58591	6.4 σ
			Local Refinement (FAF1)	58592	15.7σ
		a	Local Refinement (Npl4)	58591	5.54σ
		b	Consensus map (p97)	58590	6.6σ
		c	Consensus map (p97)	58590	6.6σ
Figure 3	State II	a	Consensus map (p97)	58590	6.6σ
			Local Refinement (Npl4)	58591	6.2σ
			Local Refinement (FAF1)	58592	15.7σ
		c	Local Refinement (FAF1)	58592	15.7σ

		e	Consensus map (ΔNFAF1)	58586	1.69σ
Figure 4	State I	a	Consensus map (p97-FAF1)	58587	6.37σ
			Local Refinement (Npl4)	58588	6.7σ
		c	Consensus map (p97-FAF1)	58587	6.37σ
	State II	a	Consensus map (p97)	58590	7.8σ
			Local Refinement (Npl4)	58591	6.2σ
			Local Refinement (FAF1)	58592	15.7σ
		c	Consensus map (p97)	58590	7.8σ
	State III	a	Consensus map (p97-FAF1)	58594	4.05σ
			Local Refinement (Npl4)	58595	5.27σ
		c	Consensus map (p97-FAF1)	58594	4.05σ
	State IV	a	Consensus map (p97-FAF1)	58597	5.13σ
			Local Refinement (Npl4)	58598	4.68σ
		c	Consensus map (p97-FAF1)	58597	5.13σ
Supplementary Figure 5	State I	a	Consensus map (p97-FAF1)	58587	2.68σ

			Local Refinement (Npl4)	58588	5.66σ
	State II	b	Consensus map (p97)	58590	5.79σ
			Local Refinement (Npl4)	58591	4.28σ
			Local Refinement (FAF1)	58592	14.07σ
	State III	c	Consensus map (p97-FAF1)	58594	3.83σ
			Local Refinement (Npl4)	58595	6.17σ
	State IV	d	Consensus map (p97)	58597	5.67σ
			Local Refinement (Npl4)	58598	6.27σ
			Local Refinement (FAF1)	59179	6.57σ
Supplementary Figure 11	State II	a	Consensus map (p97)	58590	6.6σ
			Local Refinement (Npl4)	58591	6.2σ
			Local Refinement (FAF1)	58592	15.7σ
		b	Local Refinement (Npl4)	58591	9.24σ
		c	Local Refinement (Npl4)	58591	9.30σ
		d	Local Refinement (Npl4)	58591	7.07σ

Supplementary Figure 12	ΔNFAF1	c	Consensus map (ΔNFAF1)	58586	1.92σ
Supplementary Figure 13	State I	a	Consensus map (p97-FAF1)	58587	1.54σ
			Local Refinement (Npl4)	58588	4.64σ
	State II	b	Consensus map (p97)	58590	2.51σ
			Local Refinement (Npl4)	58591	5.14σ
			Local Refinement (FAF1)	58592	14.1σ
	State III	c	Consensus map (p97-FAF1)	58594	1.69σ
			Local Refinement (Npl4)	58595	6.12σ
	State IV	d	Consensus map (p97)	58597	4.06σ
			Local Refinement (Npl4)	58598	5.72σ
			Local Refinement (FAF1)	59179	6.07σ
Supplementary Figure 14	State I	a	Consensus map (p97-FAF1)	58587	8.94σ
	State II	b	Consensus map (p97)	58590	6.84σ
	State III	c	Consensus map (p97-FAF1)	58594	7.84σ
	State IV	d	Consensus map (p97)	58597	7.57σ

Supplementary Figure 15	State I	a-d	Consensus map (p97-FAF1)	58587	6.37σ
			Local Refinement (Npl4)	58588	6.7σ
	State II		Consensus map (p97)	58590	7.8σ
			Local Refinement (Npl4)	58591	6.2σ
			Local Refinement (FAF1)	58592	15.7σ
	State III		Consensus map (p97-FAF1)	58594	4.05σ
			Local Refinement (Npl4)	58595	5.27σ
	State IV		Consensus map (p97-FAF1)	58597	5.13σ
			Local Refinement (Npl4)	58598	4.68σ

References

1. van den Boom, J. *et al.* VCP/p97 Extracts Sterically Trapped Ku70/80 Rings from DNA in Double-Strand Break Repair. *Molecular cell* **64,** 189–198; 10.1016/j.molcel.2016.08.037 (2016).

2. Weith, M. *et al.* Ubiquitin-Independent Disassembly by a p97 AAA-ATPase Complex Drives PP1 Holoenzyme Formation. *Molecular cell* **72,** 766-777.e6; 10.1016/j.molcel.2018.09.020 (2018).

3. Blythe, E. E., Olson, K. C., Chau, V. & Deshaies, R. J. Ubiquitin- and ATP-dependent unfoldase activity of P97/VCP•NPLOC4•UFD1L is enhanced by a mutation that causes multisystem proteinopathy. *Proceedings of the National Academy of Sciences of the United States of America* **114,** E4380-E4388; 10.1073/pnas.1706205114 (2017).

4. Kracht, M. *et al.* The accessory adapters FAF1, FAF2, and UBXN7 accelerate proteasomal degradation by increasing prior p97-mediated substrate unfolding. *Science advances* **12,** eaea7381; 10.1126/sciadv.aea7381 (2026).

5. Dasgupta, P. *et al.* FAF1 and FAF2 enhance human p97-UFD1-NPL4 complex unfoldase activity enabling rational design of p97 activators (2026).

6. Liao, Z., Arkinson, C. & Martin, A. Faf1 accelerates p97-mediated protein unfolding by promoting ubiquitin engagement. *bioRxiv : the preprint server for biology*; 10.1101/2025.10.27.684972 (2025).

7. Meyer, H. H., Wang, Y. & Warren, G. Direct binding of ubiquitin conjugates by the mammalian p97 adaptor complexes, p47 and Ufd1-Npl4. *The EMBO journal* **21,** 5645–5652; 10.1093/emboj/cdf579 (2002).

8. Wang, Y., Satoh, A., Warren, G. & Meyer, H. H. VCIP135 acts as a deubiquitinating enzyme during p97-p47-mediated reassembly of mitotic Golgi fragments. *The Journal of cell biology* **164,** 973–978; 10.1083/jcb.200401010 (2004).
