# Supplementary material for: Structural dynamics of substrate processing by the p97-Ufd1-Npl4-FAF1 machinery
Source: Nat Commun. 2026 Oct 25;17:11081. doi: 10.1038/s41467-026-78639-x (PMC13696688; doi:10.1038/s41467-026-78639-x)
Supplement: Supplementary file 3 [file 41467_2026_78639_MOESM3_ESM.pdf]

Description of Additional Supplementary Files

Supplementary Data 1

Description: Predicted AlphaFold3 model of only-C-termFAF1 in complex with the UT3 domain of Ufd1 and four ubiquitin molecules.

Supplementary Data 2

Description: Predicted AlphaFold3 of model ΔN FAF1 and three ubiquitins.

Supplementary Data 3

Description: Predicted model of only-C-term FAF1, Ufd1 and N terminal of p97.

Supplementary Movie 1

Description: Morphing between the four conformational states of the p97, Ufd1/Npl4 and only-C-terminal FAF1 complex. The atomic models of States I-IV are morphed sequentially to illustrate the conformational changes between the identified states.
