# Supplementary material for: Structural dynamics of substrate processing by the p97-Ufd1-Npl4-FAF1 machinery
Source: Nat Commun. 2026 Oct 25;17:11081. doi: 10.1038/s41467-026-78639-x (PMC13696688; doi:10.1038/s41467-026-78639-x)
Supplement: Supplementary file 4 [file 41467_2026_78639_MOESM8_ESM.pdf]

nature portfolio

Peer Review File

Structural dynamics of substrate processing by the p97-Ufd1-Npl4-FAF1 machinery.

Corresponding Author: Dr Christos Gatsogiannis

This file contains all reviewer reports in order by version, followed by all author rebuttals in order by version.

Version 0:

Reviewer comments:

Reviewer #1

(Remarks to the Author)
This manuscript represents one of the best structural studies of p97/Cdc48 to date. The authors undertook an impressive effort, collecting an exceptionally large dataset (~280,000 micrographs) and resolving multiple distinct structural states. These structures provide important mechanistic insights into FAF1-facilitated and UN-mediated substrate engagement and processing by p97. The work substantially extends prior structural studies, which hinted at related states but were limited by resolution and thus could not provide comparable molecular detail. The manuscript is clearly written, well illustrated, and a pleasure to read. I would therefore support publication in its current form. Below are a few suggestions that the authors may consider to further strengthen the manuscript.
Major suggestions
1. I strongly encourage the authors to include a morph movie illustrating the domain and motif movements across the different structural states. While such a movie may not be trivial to generate, it would greatly enhance reader comprehension, especially for readers less familiar with the p97 system.
2. The authors propose a sequential model in which the four resolved states act in an ordered manner. Given the data presented, this interpretation is entirely reasonable, and I would likely reach a similar conclusion. That said, a caveat is the apparent absence of substrate translocation (i.e., movement of unfolded ubiquitin through the pore). An alternative interpretation could be that only one of these states represents a fully “productive” translocation-competent state, while the others are off-pathway or preparatory. Could the authors comment on whether this alternative scenario is at least plausible based on their data?
3. The authors state that ADP·BeF(x) was used to trap the pre-hydrolysis state. Do all nucleotide-binding sites show clear density corresponding to BeF(x)? In addition, did the authors observe the ISS motif conformational changes between the subunits reported previously (Ref. 12)?
4. The structures appear to support the presence of additional ubiquitin moieties between the substrate and the unfolded ubiquitin, in contrast to the second ubiquitin reported in Ref. 23. Could this difference be attributable to the addition of FAF1 in the current study? A brief discussion of this discrepancy would be valuable.
Minor points
1. The Npl4 β-hairpin motif is mentioned on page 6 (paragraph following “The initiator ubiquitin”) but is not clearly highlighted in the figures. Explicitly indicating this motif in Figure 2 would improve clarity.
2. On page 8 (paragraph following “Interactions of Npl4 with p97”), the authors refer to a “β-strand finger (Figure 2d).” Is this the same structural element as the β-hairpin mentioned above? It is not obvious in Figure 2d where the β-strand finger is located.
3. Page 12, line 2: “though” → “through”.
4. Page 15, paragraph before “Discussion,” line 2: “od” → “of”.

Reviewer #2

(Remarks to the Author)
In this manuscript, Drakonaki and colleagues report four cryo-EM structures of a p97-Ufd1/Npl4 (UN)-FAF1 complex engaged with a polyubiquitinated artificial substrate. The principal novelty of these structures is the visualisation of an extended C-terminal helix of FAF1 that simultaneously interacts with two proximal ubiquitin moieties and the UT3 domain of UN at one extremity, and with the N-terminal domain (NTD) of p97 and possibly the SHP box of Ufd1 at the other extremity.

This configuration may provide insight into how the accessory factor FAF1 contributes to the efficient engagement and unfolding of ubiquitinated substrate.

The structures presented are of exceptional quality and reveal detailed and novel intermolecular interactions. In particular, the visualisation of FAF1 is noteworthy, as this component has not previously been structurally resolved in this context, and the positioning of its extended C-terminal helix relative to the UT3 domain of Ufd1 and proximal ubiquitin moieties is discernible despite the lower local resolution. However, no functional assays are presented to directly assess the contribution of this helical interaction with ubiquitin and the UT3 domain to substrate translocation efficiency. Moreover, as shown in Fig. 3d, the UBX–helix construct confers a modest but significant enhancement of substrate unfolding (approximately twofold), whereas full-length FAF1 results in a substantially greater effect (approximately sixfold), indicating that additional regions of FAF1 make important contributions to function. Directly linking the observed structural interactions to these functional effects would further strengthen the mechanistic conclusions

The resolution of four distinct conformational states represents an important and valuable aspect of this work. These states are interpreted as showing that the Ufd1–Npl4 complex undergoes rotation around the central axis of p97 above the D1 ring, using the assumption that FAF1 remains bound to the same p97 N-terminal domain as a reference point. However, the rationale for assuming that the FAF1-p97 NTD interaction remains fixed across all states warrants further clarification. In particular, it would be helpful to explain why this interaction is considered static, whereas the UBXL domain of Npl4 is proposed to undergo detachment and reattachment. A more explicit comparison of the relative mobility or density continuity supporting these differing behaviours would strengthen the interpretation. Additionally, these conformational states are described as differing in their ATPase states (line 326). However, the nucleotide occupancy of the individual p97 subunits is not shown in the manuscript. At the reported resolution, such differences in nucleotide binding would be expected to be discernible, and explicit visualisation or annotation of nucleotide occupancy across subunits would help support this interpretation and improve clarity. Supplementary table 2 indicates that some ADP, Mg and BEF have been modelled. No information is available for the other states.

The scale of the cryo-EM dataset used in this study is notable: the authors report collecting approximately 280,000 movies, making it “one of the largest cryo-EM datasets”, presumably to date. Given the magnitude of this data collection effort, the manuscript would benefit from explicitly stating the approximate timeframe required to acquire such a dataset, to provide transparency regarding the practical implications of the approach. Moreover, given the absence of biochemical characterisation in this manuscript demonstrating stable complex formation prior to data collection, a clear justification for adopting such an exceptionally large data-collection strategy is warranted. Providing this context is important, as studies of this scale serve as benchmarks for best practice within the cryo-EM community.

Minor edits:
Line 46: Avoid using words like “believed”. Rephrase to refer to the proposed or current model. Similarly, rephrase “therefore” to reflect that this represents the current working model, not a definitive conclusion.
Line 55, ref 10 and 22 are for cdc48 not p97.
Line 177: “we investigated the specific role” suggests that a targeted experimental analysis was performed whereas the subsequent text describes an interpretative analysis.
Line 222-23: “density corresponds to the SHP box of Ufd1”. Please show the corresponding density, not only the fitted model. This would help justify the assignment and clarify the level of confidence in this interpretation.
Line 251: UN or substrate
Line 403: “, ,” type-o
Line 581: Clarify whether p97 was expressed using the baculovirus system or in E. coli cells.
Line 592: “desired plasmid”. Include detailed information about plasmids used.
Line 612: “Ubiquitin was added”. Include information about the source for ubiquitin and indicate how it was purified.
Line 757: check reference “Le Le, T.M” or “Le, L.T”?
Supplementary table 2: Include the number of movies collected

Reviewer #3

(Remarks to the Author)
Drakonaki et al. present a high-quality cryo-EM structural investigation of the substrate-engaged p97–Ufd1–Npl4–FAF1 complex, capturing the molecular machinery of AAA-ATPase-driven ubiquitylated protein unfolding at near-atomic resolution. The authors assembled an extraordinarily large dataset (~280,000 movies) to resolve four distinct conformational states, directly correlating Npl4 "seesaw" dynamics with specific positions of the p97 ATPase seam subunit. Key advances include: the first side-chain-resolved view of initiator ubiquitin threading from the Npl4 groove through both the D1 and D2 pore rings; the discovery that only a single FAF1 molecule engages the hexameric p97 ring; a mechanistic description of how the FAF1 C-helix recruits and positions the Ufd1-UT3 domain for proximal ubiquitin engagement; and the assignment of ubiquitin-chain topology on the cis face of the complex. Collectively, this study represents a significant advance in our understanding of p97-mediated protein quality control and should be of broad interest to the cell biology, structural biology, and ubiquitin biology communities.

The structural work is outstanding; however, I recommend revisions addressing several biochemical concerns, as a number of claims appear insufficiently substantiated. I also note that I was unfortunately unable to access the maps and models

directly and would request that these be made available in a future revision.

Major Comments
(1) The linkage type of ubiquitin chains — particularly for longer chains — should be experimentally demonstrated. Although the assumption based on the experimental system is reasonable, the structure itself cannot unambiguously resolve linkage type, and biochemical validation is therefore essential. Supporting evidence from mass spectrometry, DUB treatment, or K48R mutant ubiquitin would substantially strengthen this aspect of the work.

(2) The finding that only a single FAF1 molecule engages the p97 hexamer is among the most significant claims of the paper and thus warrants orthogonal experimental support. Although the cryo-EM density is compelling, low occupancy, conformational flexibility, or averaging artefacts could in principle mask the presence of additional subunits. Solution-phase validation by mass photometry or SEC-MALS would provide important reassurance and considerably strengthen this conclusion.

(3) The placement of proximal ubiquitins relies substantially on AlphaFold predictions, which is a reasonable approach; however, the manuscript should report the associated confidence metrics (pLDDT scores and PAE plots) to allow readers to assess model quality. In addition, mutagenesis experiments targeting the predicted binding interface would be strongly recommended to validate the specificity of the proposed interactions.

(4) Assigning a sequential order to the observed states is inherently speculative. The current state definition is based on seam position and a hand-over-hand model; however, to convincingly argue that these represent successive catalytic intermediates — rather than a set of independently trapped conformations — the manuscript should more explicitly connect each state to its nucleotide occupancy and pore-loop engagement pattern. Specifically, the authors should: (i) report nucleotide identity and occupancy per subunit in D1 and D2 (where density permits), with particular attention to the seam and adjacent subunits; (ii) provide quantitative descriptors (angles, translations) for Npl4 and FAF1 motions across states and demonstrate that these correlate systematically with seam progression rather than reflecting classification artefacts; and (iii) where feasible, include a 3D variability or continuous other heterogeneity analysis (e.g. cryodrgn) to support a genuine conformational trajectory rather than a set of discrete, unrelated clusters.

(5) The proposed "tilt-engage" model is interesting and mechanistically plausible; however, the manuscript currently employs language that implies direct mechanical causation (e.g., "assistance," "swing-in/swing-out provides force/processivity") that is not directly demonstrated by the structural data alone. The authors should either moderate this language — using phrasing such as "consistent with," "may facilitate," or "is suggestive of" — and clearly delineate observation from hypothesis, or alternatively provide experimental evidence that tests mechanical coupling directly (e.g., rigidification of the FAF1 helix, systematic variation of linker length, or engineered crosslinks that restrict tilt).

Minor Comments
(6) The manuscript requires careful proofreading. Several typographical errors were noted, including "polyubiquitinaed," "crowing," and "titled," and there are likely additional instances throughout the text.

(7) The term "cis-side" should be defined clearly upon first use and employed consistently throughout both the main text and figure legends.

(8) (page 6) The hypothesis that the hydrophobic pockets disengage upon encountering bulkier or charged residues is intriguing. The authors should consider either proposing or, ideally, performing a straightforward mutational test (e.g., substituting pocket-lining hydrophobic residues with polar equivalents) to provide experimental support for this model.

(9) The functional assay effectively supports the importance of the FAF1 helix segment; however, the interpretation would be strengthened by the inclusion of binding assays alongside the activity measurements. Such controls are necessary to confirm that the observed functional defects of mutants or truncations reflect disrupted catalytic coupling rather than simply abolished complex assembly.

Version 1:

Reviewer comments:

Reviewer #1

(Remarks to the Author)
The authors have addressed all of my questions and further improved the manuscript. As noted in my first-round review, I support publication of this manuscript.

Reviewer #2

(Remarks to the Author)
I thank the authors for their careful and thoughtful responses to my comments. I appreciate the effort taken to revise the manuscript and to clarify several important points. Overall, the responses are clear and satisfactory, and they further

strengthen what is already a high-quality and impactful structural study.
The authors have responded comprehensively to all comments and have made appropriate revisions to the manuscript. I have no further concerns.
I support for publication of this work in its current form.

Reviewer #3

(Remarks to the Author)
I thank the authors for their thorough responses to my concerns. All of my previous concerns have been fully addressed and I am satisfied on those points. In my first review I was unable to access the maps and models. I have now inspected them and this raises several concerns about how the maps were generated, displayed, and interpreted. I consider these important to resolve before publication, but I believe they are all addressable.

(1) The Methods state that the final maps were built with phenix.combine_focused_maps from separately, locally refined regions. Building composites from local refinements is an accepted approach and I have no objection in principle. My concern is that, by default, this tool normalizes each input map before combining (each is scaled to an r.m.s. of 1.0) and weights regions by local map-to-model correlation. This rescales a genuinely low-occupancy, flexible region to the same density level as the well-ordered core. The difference here is large: per Supplementary Table 2 the p97 motor is at about 3.0 to 3.2 A, whereas Npl4 is at 4.5 to 5.8 A and FAF1 only at 8.6 to 12 A. Equalizing these densities asserts a comparable degree of order that the data do not support and makes the flexible cis-side appear far better defined than it is. I ask the authors either to rebuild the composites without per-map normalization (for example normalize=False, or a maximum-value composite from locally refined maps such as ChimeraX volume maximum), or at minimum to also show the unsharpened consensus reconstruction of each state alongside with the maps and not bury them in the supplement so the true relative density of the UN and FAF1 regions is visible.

(2) The flexible cis-side is presented as discrete, well-defined states. The four states are defined by the p97 seam position, which rests on the high-resolution core, so I do not dispute that the seam configurations are real. But the normalization makes the Ufd1-Npl4, FAF1, and ubiquitin density appear to adopt a single defined conformation per state, when the data indicate a continuum. The authors in fact acknowledge this in their rebuttal: their own per-state 3D variability analysis reveals continuous heterogeneity within each state, including a continuous tilt of FAF1, and describes a continuous motion linking the four states (Rebuttal Figure 1). This important information is currently confined to the rebuttal. I ask that a flexibility or continuous-heterogeneity analysis be included in the manuscript itself. Since the authors report that variability analysis on the full dataset was dominated by the large motor motions and did not converge, a deep generative approach such as cryoDRGN would be well suited to capturing the combined large-scale and local heterogeneity across the whole particle set. At minimum, some analysis of the continuum should appear in the main text so that the discrete states are framed as representative points along a continuous trajectory.

(3) Side chains should not be modeled or interpreted in the low-resolution regions. At the resolutions reported for Npl4 (4.5 to 5.8 A) and for FAF1 and the associated ubiquitins (8.6 to 12 A), side chains are not resolved and reliable main-chain register is not achievable. At least in the maps I have seen I dont think ubiquitin can be unambiguously placed. Supplementary Figure 11, which shows detailed interactions of the distal ubiquitins with the Npl4 tower and colours the surface by hydrophobicity, implies a level of side-chain detail the density does not support. I ask that these regions be shown as rigid-body-docked or backbone-level models only, without interaction claims that depend on specific side-chain placements. I am not disputing the FAF1 C-helix to UT3 interface itself, which is supported by the authors' biochemistry and by independent recent work; my point is only that side-chain detail should not be read out of this particular density.

(4) The ubiquitin densities show features consistent with mask artefacts, with sharp boundaries suggesting density has been carved by the mask rather than reflecting its true extent. I credit the authors for already omitting ubiquitin moieties that lacked density (Supplementary Figure 8). I ask them to extend the same transparency to the retained ubiquitins by showing the unmasked, unsharpened maps in these regions, so the reader can judge which density is genuine.

(5) It should be stated which map is displayed in each figure. I found it difficult to get similar clear representations of fits with the composite maps provided and to assess the quality of the fits and it is not always clear which map underlies each panel. Please state explicitly, for every panel showing density, which map is shown (consensus, locally refined, or composite, and which state). Please at least for me include with the contour level and EMDB accession; a short table mapping figures to maps would be ideal.

Version 2:

Reviewer comments:

Reviewer #3

(Remarks to the Author)
I thank the authors for a constructive revision. The new 3D variability analysis, the new Supplementary Figure 13, the improved local refinement of the State IV FAF1/UT3 region, the revised Supplementary Figure 11, and the new

Supplementary Table 4 all move the manuscript in the right direction, and my concerns about the biological conclusions are resolved. The central claims, namely, a single FAF1 per p97 hexamer, docking of the UBX domain onto the same NTD that binds the Ufd1 SHP box, engagement of proximal ubiquitins by the FAF1 C-helix and the Ufd1 UT3 domain, and four seam-defined conformational states, are well supported by the data as now presented.

However, some of the changes described in the response letter are either not evident in the submitted files or have not been implemented consistently throughout. The revision has also introduced a small number of internal inconsistencies. I would ask the authors to address the following points before acceptance.

(1) The response states that the Discussion was expanded to emphasize that the identified states represent distinct conformational ensembles while capturing the intrinsic flexibility of FAF1. I could not identify this specific clarification in the submitted Discussion; in particular, the term “conformational ensemble” does not occur in the manuscript. The new explicit treatment of continuous variability appears instead as a paragraph at the end of the Results. Please either incorporate the clarification promised in the response into the Discussion or indicate explicitly where this point has been addressed.

(2) My earlier comment about modelling and interpreting side chains in lower-resolution regions has been addressed for Figures 1–3 and for Supplementary Figure 11, and I am satisfied with the revised Supplementary Figure 11. The same issue, however, remains in Supplementary Figure 16, which was not correspondingly modified. That figure annotates individual residues on surfaces coloured by hydrophobicity, in states for which Supplementary Table 2 reports Npl4 local resolutions of 5.0, 4.48 and 5.82 Å, respectively. Please either present these interfaces at backbone/rigid-body level without residue-specific annotation, or state the local resolution in the legend together with an explicit statement that the side-chain positions are not determined by the density and are shown only to indicate the region involved. There is also a minor labeling inconsistency: the heading of Supplementary Figure 16 refers to States I, II and IV, whereas the panels shown are States I, III and IV.

(3) The improved State IV FAF1/UT3 local refinement added in response to my concern about possible mask artefacts reports a resolution of approximately 6.8 Å in Supplementary Figure 5. However, its FSC panel does not show the same full set of curves as the other refinements. Instead, the displayed curves are labelled “Auto-tight Mask,” “Auto-tight Mask, corr” and “Input Mask,” whereas the other panels show the No Mask, Loose, Tight and Corrected curves. In addition, the corrected curve appears to drop extremely abruptly over a very narrow resolution range. Without the corresponding unmasked and loose-mask FSC curves, it is difficult to exclude an effect of an overly restrictive mask, which is particularly relevant because this refinement was introduced in response to my concern about masking. Please show the full set of FSC curves for this refinement, consistent with the other panels.

(4) I appreciate the addition of Supplementary Figure 13. However, the unmasked consensus maps there are shown for the Npl4 and FAF1/UT3 region as a whole at a single contour, so the individual distal ubiquitins Ub(1), Ub(2) and Ub(3) still cannot readily be assessed against the unmasked density. The per-ubiquitin panels in Supplementary Figure 11b–d appear to show locally refined density, but the legend does not specify whether the displayed maps are unmasked. In panels c and d, portions of the modelled β-sheet also appear to extend outside the displayed density envelope.

(5) The new 3D variability analysis is a useful addition, but it was performed separately within each already-classified state. Residual heterogeneity within an already classified subset can include conformations extending toward neighbouring classes, so the observation that the extreme frames of State II point toward State III is consistent with, but does not by itself establish, a sequential trajectory. I therefore ask the authors to soften “supporting sequential state transition” in the Results and “supporting the proposing sequential model” in the legend of Supplementary Figure 17 to wording such as “consistent with the proposed sequential transition/model,” and to add one sentence acknowledging this limitation. Generally cryodrgn analysis should do a better job here or at least not run variability on pooled particles and not individual states.

(6) Contour levels are currently given only in the legend of Figure 1. Figures 2, 3 and 4 and the supplementary figures showing cryo-EM density do not provide them, although my previous request was for contour level together with the corresponding accession code. Supplementary Table 4 is a helpful addition, but it is organized per figure rather than per panel, despite its title, and does not cover the supplementary density figures, including at least Supplementary Figures 5, 11, 12, 13, 14 and 15.

The table also disagrees with the main-figure legends. The legend of Figure 2 lists EMD-58590, EMD-58591 and EMD-58592, whereas Supplementary Table 4 lists only EMD-58590 and EMD-58592. Similarly, the legend of Figure 3 lists EMD-58590, EMD-58591 and EMD-58592, whereas the table lists only EMD-58592. The newly deposited State IV FAF1 refinement, EMD-59179, is also absent from Supplementary Table 4. Please complete and reconcile the table and provide contour levels for the density panels.

(7) Figure 1 displays three separate maps at independently chosen contour thresholds: 15.7σ for FAF1, 6.2σ for the UN tower and 7.8σ for p97. I accept that these are not composite maps and that the poorer local resolution of the FAF1/ubiquitin region is visually apparent. Nevertheless, independently chosen thresholds mean that relative density strength or apparent occupancy cannot be compared directly between the three displayed components. One sentence in the legend stating that the components are contoured independently and that apparent occupancy or relative density strength should therefore not be compared between them would remove any ambiguity.

Overall, the manuscript has improved substantially, and I consider the major biological and mechanistic conclusions adequately supported. The remaining points concern consistency of presentation, transparency of map provenance and validation, and ensuring that interpretations are stated at a level appropriate to the local resolution. I do not think these issues require substantial new experiments or a fundamentally different analysis, but they should be resolved before publication.

Open Access This Peer Review File is licensed under a Creative Commons Attribution 4.0 International License, which permits use, sharing, adaptation, distribution and reproduction in any medium or format, as long as you give appropriate credit to the original author(s) and the source, provide a link to the Creative Commons license, and indicate if changes were made.
In cases where reviewers are anonymous, credit should be given to 'Anonymous Referee' and the source.
The images or other third party material in this Peer Review File are included in the article's Creative Commons license, unless indicated otherwise in a credit line to the material. If material is not included in the article's Creative Commons license and your intended use is not permitted by statutory regulation or exceeds the permitted use, you will need to obtain permission directly from the copyright holder.
To view a copy of this license, visit https://creativecommons.org/licenses/by/4.0/

We thank the reviewers for their helpful comments on our work and useful guidance in revising our paper. Below is a point-by-point response to all comments and a detailed description of all changes we have made to our manuscript after considering their suggestions.

Reviewers' comments:

Reviewer 1

This manuscript represents one of the best structural studies of p97/Cdc48 to date. The authors undertook an impressive effort, collecting an exceptionally large dataset (~280,000 micrographs) and resolving multiple distinct structural states. These structures provide important mechanistic insights into FAF1-facilitated and UN-mediated substrate engagement and processing by p97. The work substantially extends prior structural studies, which hinted at related states but were limited by resolution and thus could not provide comparable molecular detail. The manuscript is clearly written, well illustrated, and a pleasure to read. I would therefore support publication in its current form. Below are a few suggestions that the authors may consider to further strengthen the manuscript.

We thank the reviewer for the positive evaluation of our manuscript.

Major suggestions

1. *I strongly encourage the authors to include a morph movie illustrating the domain and motif movements across the different structural states. While such a movie may not be trivial to generate, it would greatly enhance reader comprehension, especially for readers less familiar with the p97 system.*

We agree with the reviewer and added a morph movie across the different structural states (Supplementary Movie 1).

2. *The authors propose a sequential model in which the four resolved states act in an ordered manner. Given the data presented, this interpretation is entirely reasonable, and I would likely reach a similar conclusion. That said, a caveat is the apparent absence of substrate translocation (i.e., movement of unfolded ubiquitin through the pore). An alternative interpretation could be that only one of these states represents a fully "productive" translocation-competent state, while the others are off-pathway or preparatory. Could the authors comment on whether this alternative scenario is at least plausible based on their data?*

We appreciate the reviewer's insightful question. Although we used ADP.BeF(x) to mimic the pre-hydrolysis ATP-bound state, which precludes processive substrate translocation, all four structures reveal that p97 adopts the expected helical arrangement and the unfolded substrate extends through the pore to a consistent position. Notably, these features are characteristic of a fully 'productive' translocation-competent state, as previously repeatedly described for p97 in previous studies and seen in other AAA unfoldases following the 'hand-over-hand' mechanism. We therefore propose that each of the four states is capable of continuing the cycle in the presence of ATP, supporting our sequential model.

3. *The authors state that ADP·BeF(x) was used to trap the pre-hydrolysis state. Do all nucleotide-binding sites show clear density corresponding to BeF(x)?*

We thank the reviewer for this comment. The nucleotide binding pockets of subunits A-E in both D1

and D2 rings contain density consistent with ADP.BeF(x), whereas the seam subunit F shows density corresponding to ADP (New Supplementary Figure 13).

4. *In addition, did the authors observe the ISS motif conformational changes between the subunits reported previously (Ref. 12)?*

We indeed observe the ISS motif conformational changes between subunits, consistent with the previous report (Ref. 12). The ISS motif adopts a triangular loop conformation that inserts into the neighboring subunit's nucleotide-binding site, coordinating the nucleotide with additional residues in D2. This conformational change is observed when ADP.BeF(x) is engaged, and is limited to two to four consecutive binding sites, similar to Ref. 12. Notably, this conformation is linked to the spiral staircase conformation.

5. *The structures appear to support the presence of additional ubiquitin moieties between the substrate and the unfolded ubiquitin, in contrast to the second ubiquitin reported in Ref. 23. Could this difference be attributable to the addition of FAF1 in the current study? A brief discussion of this discrepancy would be valuable.*

We apologize for the misunderstanding and would like to clarify that our study does not suggest any discrepancy with Ref. 23 regarding the arrangement of the initiator and second ubiquitin moiety. As shown in Fig. 3, we propose that Ub(-1) is at least the second ubiquitin in our structure, with Ub(1-) positioned at the expected distance and orientation to the unfolded ubiquitin. We rather observe an additional proximal ubiquitin, Ub(x), bound to UT3, which is further away and not oriented for direct linkage with either Ub(-1) or the unfolded ubiquitin (Ub(0)). The most plausible explanation is that additional ubiquitins, not resolved in our structure, are positioned between Ub(-1) and Ub(x), leading us to assign it a non-specific proximal designation. The inclusion of FAF1 had a significant impact, as its C-terminal helix stabilizes UT3, enabling direct visualization and resolution of its interactions with these two ubiquitins for the first time. We have revised the relevant sentence to clarify this point.

Minor points

6. *The Npl4 β-hairpin motif is mentioned on page 6 (paragraph following "The initiator ubiquitin") but is not clearly highlighted in the figures. Explicitly indicating this motif in Figure 2 would improve clarity.*

Thank you for the suggestion We revised Figure 2.

7. *On page 8 (paragraph following "Interactions of Npl4 with p97"), the authors refer to a "β-strand finger (Figure 2d)." Is this the same structural element as the β-hairpin mentioned above? It is not obvious in Figure 2d where the β-strand finger is located.*

It is indeed the same structural element. We revised the text and indicated this motif in Revised Figure 2d.

8. *Page 12, line 2: "though" → "through".*

Done

9. *Page 15, paragraph before "Discussion," line 2: "od" → "of".*

Done

Reviewer 2

In this manuscript, Drakonaki and colleagues report four cryo-EM structures of a p97-Ufd1/Npl4 (UN)-FAF1 complex engaged with a polyubiquitinated artificial substrate. The principal novelty of these structures is the visualisation of an extended C-terminal helix of FAF1 that simultaneously interacts with two proximal ubiquitin moieties and the UT3 domain of UN at one extremity, and with the N-terminal domain (NTD) of p97 and possibly the SHP box of Ufd1 at the other extremity. This configuration may provide insight into how the accessory factor FAF1 contributes to the efficient engagement and unfolding of ubiquitinated substrate.

The structures presented are of exceptional quality and reveal detailed and novel intermolecular interactions. In particular, the visualisation of FAF1 is noteworthy, as this component has not previously been structurally resolved in this context, and the positioning of its extended C-terminal helix relative to the UT3 domain of Ufd1 and proximal ubiquitin moieties is discernible despite the lower local resolution.

We thank the reviewer for the positive evaluation of our manuscript.

Major suggestions

10. *However, no functional assays are presented to directly assess the contribution of this helical interaction with ubiquitin and the UT3 domain to substrate translocation efficiency. Moreover, as shown in Fig. 3d, the UBX–helix construct confers a modest but significant enhancement of substrate unfolding (approximately twofold), whereas full-length FAF1 results in a substantially greater effect (approximately sixfold), indicating that additional regions of FAF1 make important contributions to function. Directly linking the observed structural interactions to these functional effects would further strengthen the mechanistic conclusions.*

We appreciate the reviewer's insightful question. A recent study has demonstrated the importance of the helical interaction between FAF1 and the UT3 domain for substrate translocation efficiency (doi: 10.1126/sciadv.aea7381). Specifically, in accordance to our detailed structural insights, mutants V507A/K508A of FAF1, which disrupt this interface, exhibit reduced substrate unfolding, providing direct evidence for the crucial role of this interaction. This finding is now cited in the revised manuscript. Furthermore, full-length FAF1's additional ubiquitin binding sites may further enhance translocation efficiency by further optimization of polyUb positioning. However, as the detailed interactions and dynamics remain unresolved, we prefer not to speculate on these aspects.

11. *The resolution of four distinct conformational states represents an important and valuable aspect of this work. These states are interpreted as showing that the Ufd1–Npl4 complex undergoes rotation around the central axis of p97 above the D1 ring, using the assumption that FAF1 remains bound to the same p97 N-terminal domain as a reference point. However, the rationale for assuming that the FAF1-p97 NTD interaction remains fixed across all states warrants further clarification. In particular, it would be helpful to explain why this interaction is considered static, whereas the UBXL domain of Npl4 is proposed to undergo detachment and reattachment. A more explicit comparison of the relative mobility or density continuity supporting these differing behaviours would strengthen the interpretation.*

We have added a morph movie (Supplementary Movie 1) to illustrate the conformational changes across the different states. Please note: while FAF1 remains bound to the same p97 N-terminal domain, its characteristic inward-outward tilt and the simultaneous constrained rotation of the

Ufd1-Npl4 complex can fully accommodate the sequential spiral staircase rearrangements of p97, which is consistent with the established 'hand-over-hand' mechanism from a plethora of previous studies. We also performed 3D VA analysis on its individual dataset of each state, supporting a sequential trajectory (please refer to comment 30 of Reviewer 3).

12. *Additionally, these conformational states are described as differing in their ATPase states (line 326). However, the nucleotide occupancy of the individual p97 subunits is not shown in the manuscript. At the reported resolution, such differences in nucleotide binding would be expected to be discernible, and explicit visualisation or annotation of nucleotide occupancy across subunits would help support this interpretation and improve clarity. Supplementary table 2 indicates that some ADP, Mg and BEF have been modelled. No information is available for the other states.*

We thank the reviewer for this comment. To address this, we have added Supplementary Figure 13, which illustrates the nucleotide binding sites for each conformational state. Additionally, we have updated Supplementary Table 2 to include the relevant information for all states.

13. *The scale of the cryo-EM dataset used in this study is notable: the authors report collecting approximately 280,000 movies, making it "one of the largest cryo-EM datasets", presumably to date. Given the magnitude of this data collection effort, the manuscript would benefit from explicitly stating the approximate timeframe required to acquire such a dataset, to provide transparency regarding the practical implications of the approach. Moreover, given the absence of biochemical characterisation in this manuscript demonstrating stable complex formation prior to data collection, a clear justification for adopting such an exceptionally large data-collection strategy is warranted. Providing this context is important, as studies of this scale serve as benchmarks for best practice within the cryo-EM community.*

We appreciate the reviewer's acknowledgement of this aspect of our work. The data collection for the only-C-terminal FAF1 dataset spanned 12.5 days, and 4.2 days for the ΔN FAF1 dataset, utilizing a KRIOS G4 equipped with a Falcon 4i detector. The substantial conformational heterogeneity of the complex, evident in preliminary classifications, necessitated such a dataset. Initial processing of a smaller dataset (530,983 particles from 20,702 movies) confirmed that a significant population of particles (34 %) included all components of the complex, although resolution in the UN/FAF1 region was limited due to conformational heterogeneity. This motivated us to collect a large number of particles for classification, enabling the resolution of multiple conformational states. We acknowledge that datasets of this scale pose significant practical challenges, including data acquisition, processing time, and computational demands. To provide context for the community, we have clarified these aspects in the revised manuscript, highlighting the opportunities and challenges associated with large-scale cryo-EM data collection.

Minor edits:

14. *Line 46: Avoid using words like "believed". Rephrase to refer to the proposed or current model. Similarly, rephrase "therefore" to reflect that this represents the current working model, not a definitive conclusion.*

We have rephrased the respective sentence.

15. *Line 55, ref 10 and 22 are for cdc48 not p97.*

We thank the reviewer for pointing this out. Done.

16. *Line 177: "we investigated the specific role" suggests that a targeted experimental analysis was performed whereas the subsequent text describes an interpretative analysis.*

We revised the manuscript, as per the reviewer's suggestion.

17. *Line 222-23: "density corresponds to the SHP box of Ufd1". Please show the corresponding density, not only the fitted model. This would help justify the assignment and clarify the level of confidence in this interpretation.*

Thank you for this comment. We revised Figure 2.

18. *Line 251: UN or substrate*

Done

19. *Line 403: ", ," type-o*

Done

20. *Line 581: Clarify whether p97 was expressed using the baculovirus system or in E. coli cells.*

We have updated the Methods section of the manuscript.

21. *Line 592: "desired plasmid". Include detailed information about plasmids used.*

We have included detailed information about plasmids used in Supplementary Table 3.

22. *Line 612: "Ubiquitin was added". Include information about the source for ubiquitin and indicate how it was purified.*

We added this information in the Materials and Method section of the manuscript.

23. *Line 757: check reference "Le Le, T.M" or "Le, L.T"?*

We thank the reviewer for pointing this out. Done.

24. *Supplementary table 2: Include the number of movies collected*

Done

Reviewer 3

Drakonaki et al. present a high-quality cryo-EM structural investigation of the substrate-engaged p97–Ufd1–Npl4–FAF1 complex, capturing the molecular machinery of AAA-ATPase-driven ubiquitylated protein unfolding at near-atomic resolution. The authors assembled an extraordinarily large dataset (~280,000 movies) to resolve four distinct conformational states, directly correlating Npl4 "seesaw" dynamics with specific positions of the p97 ATPase seam subunit. Key advances include: the first side-chain-resolved view of initiator ubiquitin threading from the Npl4 groove through both the D1 and D2 pore rings; the discovery that only a single FAF1 molecule engages the hexameric p97 ring; a mechanistic description of how the FAF1 C-helix recruits and positions the Ufd1-UT3 domain for proximal ubiquitin engagement; and the assignment of ubiquitin-chain topology on the cis face of the complex. Collectively, this study represents a significant advance in our understanding of p97-mediated protein quality control and should be of broad interest to the cell biology, structural biology, and ubiquitin biology communities.

The structural work is outstanding; however, I recommend revisions addressing several biochemical concerns, as a number of claims appear insufficiently substantiated. I also note that I was

unfortunately unable to access the maps and models directly and would request that these be made available in a future revision.

We appreciate the Reviewer's positive feedback and thoughtful questions. As requested, we have compiled a downloadable package containing all maps and models, which we have forwarded to the editor for distribution.

Major Suggestions

25. *The linkage type of ubiquitin chains — particularly for longer chains — should be experimentally demonstrated. Although the assumption based on the experimental system is reasonable, the structure itself cannot unambiguously resolve linkage type, and biochemical validation is therefore essential. Supporting evidence from mass spectrometry, DUB treatment, or K48R mutant ubiquitin would substantially strengthen this aspect of the work.*

We thank the reviewer for raising this point. We agree that biochemical validation of the substrate used in this study is necessary. Therefore, we conducted poly-ubiquitination reactions of the Eos substrate with ubiquitin mutants, K48R and K63R. The Eos substrate was separated from unanchored ubiquitin chains via Strep pulldown (See updated Material and Methods). With our set of ubiquitination enzymes (gp78-Ubc7), no poly-ubiquitination is observed with K48R ubiquitin, demonstrating that the linkage type in our substrate is K48 (Supplementary Figure 1b).

26. *The finding that only a single FAF1 molecule engages the p97 hexamer is among the most significant claims of the paper and thus warrants orthogonal experimental support. Although the cryo-EM density is compelling, low occupancy, conformational flexibility, or averaging artefacts could in principle mask the presence of additional subunits. Solution-phase validation by mass photometry or SEC-MALS would provide important reassurance and considerably strengthen this conclusion.*

We performed mass photometry measurements using the Refeyn TwoMP Mass Photometer (see updated Materials and Methods). Under non-crosslinked conditions the individual components of the complex were detectable (data not shown), but not the intact complex, due to the required low nanomolar concentrations required for the instrument. After crosslinking with DSSO, distinct subcomplexes (Supplementary Figure 6) were visible suggesting a stoichiometry of one FAF1 per p97 (hexamer)-Ufd1-Npl4. Consistent with this observation functional unfolding assays of the polyubiquitinated substrate Eos performed at varying ratios of FAF1 to p97-UN confirmed that maximal simulation of p97 unfolding activity was reached at a ratio of one FAF1 per p97 (hexamer)-Ufd1-Npl4. Increasing the FAF1 concentration beyond this ratio didn't further enhance the unfolding rate, suggesting that one FAF1 is enough to stimulate the p97-Ufd1-Npl4 complex.

27. *The placement of proximal ubiquitins relies substantially on AlphaFold predictions, which is a reasonable approach; however, the manuscript should report the associated confidence metrics (pLDDT scores and PAE plots) to allow readers to assess model quality. In addition, mutagenesis experiments targeting the predicted binding interface would be strongly recommended to validate the specificity of the proposed interactions.*

We appreciate the reviewer's suggestion and have added confidence metrics for the AlphaFold models used in this study (Supplementary Figure 8). To experimentally validate the predicted interaction interfaces between Ufd1 and proximal ubiquitins, we introduced site-specific mutations in Ufd1 that disrupt ubiquitin binding. Our substrate unfolding assays show that mutations targeting the Ub(-1) site (L53K) or both Ub(-1) and Ub(x) sites strongly impair substrate unfolding, whereas mutation of

the Ub(x) site (S27R+S29D) alone has a milder, yet significant effect. Notably, these mutations do not affect the assembly of the UN heterodimer (equal amounts of Npl4 are co-purified via the tag on Ufd1) and show no signs of aggregation in gel filtration (Supplementary 9). These results directly support the validity of the proposed interactions.

28. *Assigning a sequential order to the observed states is inherently speculative. The current state definition is based on seam position and a hand-over-hand model; however, to convincingly argue that these represent successive catalytic intermediates — rather than a set of independently trapped conformations — the manuscript should more explicitly connect each state to its nucleotide occupancy and pore-loop engagement pattern. Specifically, the authors should: (i) report nucleotide identity and occupancy per subunit in D1 and D2 (where density permits), with particular attention to the seam and adjacent subunits;*

We have included the nucleotide binding sites in Supplementary Figure 13. We have also added a morph movie (Supplementary Movie 1) to illustrate the conformational changes across the different states. Please note that the characteristic inward-outward tilt of FAF1 and the simultaneous constrained rotation of the Ufd1-Npl4 complex can fully accommodate the sequential spiral staircase rearrangements of p97, which is consistent with the 'hand-over-hand' model.

29. *(ii) provide quantitative descriptors (angles, translations) for Npl4 and FAF1 motions across states and demonstrate that these correlate systematically with seam progression rather than reflecting classification artefacts;*

We appreciate the reviewer's suggestion and have incorporated quantitative descriptors for Npl4 and FAF1 motions across all states.

30. *and (iii) where feasible, include a 3D variability or continuous other heterogeneity analysis (e.g. cryodrgn) to support a genuine conformational trajectory rather than a set of discrete, unrelated clusters.*

We thank the reviewer for their valuable suggestion. We performed 3D variability analysis (3D VA) on the full dataset; however, this initial attempt focused primarily on large structural rearrangements in p97's spiral staircase. Therefore, we applied 3D VA to each particle stack independently, that yielded the reconstructions of the four states presented in this study. Given the defined spiral staircase conformation in each stack, we masked the cis-side of the complex and analyzed variability within this region. This analysis revealed continuous heterogeneity *within* each state, explaining the limited resolution observed in this region. For example, we observed a characteristic tilt in FAF1 that varied continuously between two extreme positions around the consensus structure of the respective state. Consequently, 3D VA within each state revealed multiple substates and intermediates along these conformational changes. When we combine the 3DVA results, we thus observe a continuous motion linking the four states (Rebuttal Figure 1). Although the 3DVA was not succesful for the full dataset and we had to combine and sort results from independent 3DVA analysis of the four stacks, this supports that these four states rather represent a genuine conformational trajectory rather than a collection of discrete, unrelated clusters. However, each substate contains a limited number of particles, preventing us from achieving higher resolution.

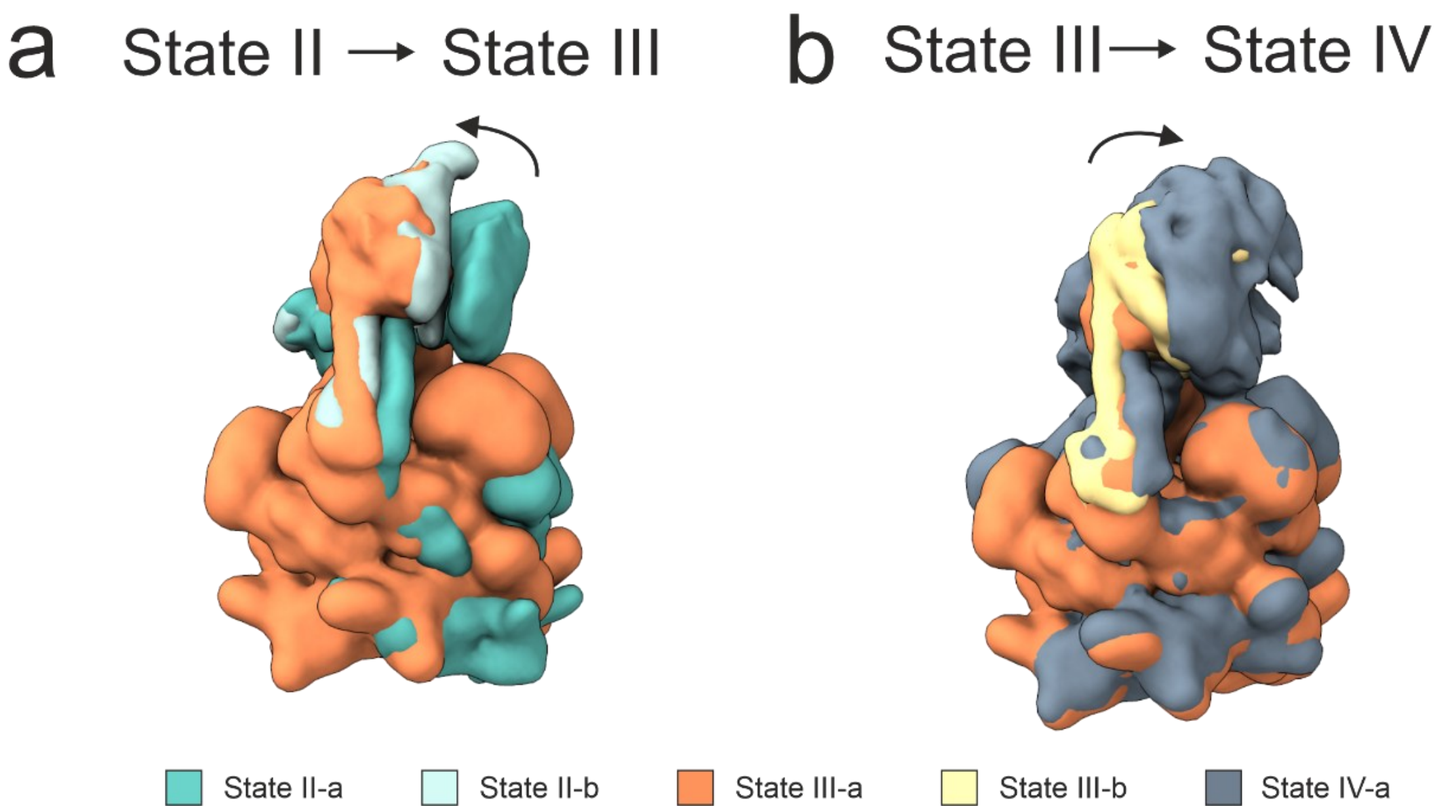

Rebuttal Figure 1: 3D variability analysis of the dataset of State II and State III. a) We show the two extreme frames of 3VA of the dataset of State-II (State-II-a; teal, State-II-b; cyan). We superposed them with the first extreme frame of State-III. Note that the two substates of State-II agree with a continuous movement (e.g. outward tilt of FAF1) towards State-III, supporting the proposing sequential model. **b)** Similarly, two extremes frames of 3D VA of State-III (State-III-a; orange, State-III-b; yellow) and first extreme frame of State-IV (State-IV-a; gray) are shown (e.g. inward tilt of FAF1).

31. *The proposed "tilt-engage" model is interesting and mechanistically plausible; however, the manuscript currently employs language that implies direct mechanical causation (e.g., "assistance," "swing-in/swing-out provides force/processivity") that is not directly demonstrated by the structural data alone. The authors should either moderate this language — using phrasing such as "consistent with," "may facilitate," or "is suggestive of" — and clearly delineate observation from hypothesis, or alternatively provide experimental evidence that tests mechanical coupling directly (e.g., rigidification of the FAF1 helix, systematic variation of linker length, or engineered crosslinks that restrict tilt).*

We have revised the manuscript to moderate the language.

Minor Comments

32. *The manuscript requires careful proofreading. Several typographical errors were noted, including "polyubiquitinaed," "crowing," and "titled," and there are likely additional instances throughout the text.*

Thank you for pointing this out. The revised manuscript has been carefully edited to eliminate any remaining typos.

33. *The term "cis-side" should be defined clearly upon first use and employed consistently throughout both the main text and figure legends.*

Done

34. *(page 6) The hypothesis that the hydrophobic pockets disengage upon encountering bulkier or charged residues is intriguing. The authors should consider either proposing or, ideally, performing a straightforward mutational test (e.g., substituting pocket-lining hydrophobic residues with polar equivalents) to provide experimental support for this model.*

We appreciate the reviewer's suggestion and have performed a mutational test to assess the functional importance of substrate interactions with the Npl4 groove. Specifically, we introduced mutations (E446K+Y447A) that abolish binding between the groove and the helix of the unfolded ubiquitin, and found that these mutations significantly reduce activity in the Eos unfolding assay (Supplementary Figure 7a,b). These results support the functional relevance of substrate engagement with the Npl4 groove. In light of these findings, we have revised the text to clarify that the proposed disengagement mechanism remains speculative, and instead we favor a model where the substrate disengages from the groove upon initiation of ATP-driven threading, rather than progressively translocating along the groove.

35. *The functional assay effectively supports the importance of the FAF1 helix segment; however, the interpretation would be strengthened by the inclusion of binding assays alongside the activity measurements. Such controls are necessary to confirm that the observed functional defects of mutants or truncations reflect disrupted catalytic coupling rather than simply abolished complex assembly.*

We thank the reviewer for his constructive suggestion. We biotinylated full- length FAF1 and the two truncations of the Helix-UBX (528-650 and 542-650) of FAF1. We incubated the proteins with p97 and carried out pulldown assays (See updated Materials and Methods). We examined the amounts of p97 that co-isolated with FAF1 truncations. Full-length FAF1 and its truncated versions showed similar binding to p97 (Supplementary Figure 10). The loss of unfolding boost in the truncations is therefore not due to abolished complex formation with p97.

We thank the reviewers for their additional comments on our work. Below, we provide a point-by-point response to each comment and describe the revisions made to the manuscript in light of their suggestions.

Reviewers' comments:

Reviewer 1

The authors have addressed all of my questions and further improved the manuscript. As noted in my first-round review, I support publication of this manuscript.

We thank the reviewer for their supportive feedback and for recommending the manuscript for publication.

Reviewer 2

I thank the authors for their careful and thoughtful responses to my comments. I appreciate the effort taken to revise the manuscript and to clarify several important points. Overall, the responses are clear and satisfactory, and they further strengthen what is already a high-quality and impactful structural study. The authors have responded comprehensively to all comments and have made appropriate revisions to the manuscript. I have no further concerns.

I support for publication of this work in its current form.

We thank the reviewer for their positive assessment and for their support of the publication of our work. We are grateful for the constructive feedback that helped us improve the clarity and quality of this study.

Reviewer 3

I thank the authors for their thorough responses to my concerns. All of my previous concerns have been fully addressed and I am satisfied on those points. In my first review I was unable to access the maps and models. I have now inspected them and this raises several concerns about how the maps were generated, displayed, and interpreted. I consider these important to resolve before publication, but I believe they are all addressable.

We thank the reviewer for their careful assessment of our revised manuscript and for confirming that our previous responses were satisfactory. We appreciate the reviewer's detailed inspection of the maps and models. We have carefully addressed the new concerns regarding the generation, display, and interpretation of the cryo-EM volumes. Our detailed responses to these points are provided below, and we hope they fully address the reviewer's concerns regarding the structural data.

1. *The Methods state that the final maps were built with phenix.combine_focused_maps from separately, locally refined regions. Building composites from local refinements is an accepted approach and I have no objection in principle. My concern is that, by default, this tool normalizes each input map before combining (each is scaled to an r.m.s. of 1.0) and weights regions by local map-to-model correlation. This rescales a genuinely low-occupancy, flexible region to the same density level as the well-ordered core. The difference here is large: per Supplementary Table 2 the p97 motor is at about 3.0 to 3.2 A, whereas Npl4 is at 4.5 to 5.8 A and FAF1 only at 8.6 to 12 A. Equalizing these densities asserts a comparable degree of order that the data do not support and makes the flexible cis-side appear far better defined than it is. I ask the authors either to rebuild the composites without per-map normalization (for*

example normalize=False, or a maximum-value composite from locally refined maps such as ChimeraX volume maximum), or at minimum to also show the unsharpened consensus reconstruction of each state alongside with the maps and not bury them in the supplement so the true relative density of the UN and FAF1 regions is visible.

We thank the reviewer for their insightful comments regarding the normalization of composite maps. To address these concerns, we would like to clarify the following points:

The composite maps generated using phenix.combine_focused_maps are not featured in any of the manuscript's figures. These volumes were created solely to facilitate a comprehensive inspection of the overall complex structure, to assist the reviewer, and to enable upload to the EMDB. This composite volume allows for a direct comparison by fitting all chains of the submitted PDB at a single threshold value.

Structural interpretation and model building were performed using the consensus reconstructions and locally refined maps, filtered according to their respective local resolutions. We discuss the heterogeneity of the *cis*-side extensively in the manuscript and have added further text to highlight this. Furthermore, it is visually evident in Figure 1 that the locally refined and filtered map of the FAF1/Ub region is significantly less resolved than the p97/Npl4 region, demonstrating that these structural regions do not possess a comparable degree of order. This approach is widely accepted in the field.

To address the reviewer's concerns, we have uploaded the consensus map (prior to local refinements) as the main EMDB entry, linked to the locally refined maps. The normalized composite map is included as an additional file for the main EMDB entry of the respective state, and we are updating the EMDB entries to include both normalized and non-normalized composite maps.

Finally, we now explicitly state in the main text and figure legends that locally refined maps are shown in the main figures (see response to comment 5), while the consensus maps and refinement workflows are detailed in the supplement.

2. *The flexible cis-side is presented as discrete, well-defined states. The four states are defined by the p97 seam position, which rests on the high-resolution core, so I do not dispute that the seam configurations are real. But the normalization makes the Ufd1-Npl4, FAF1, and ubiquitin density appear to adopt a single defined conformation per state, when the data indicate a continuum. The authors in fact acknowledge this in their rebuttal: their own per-state 3D variability analysis reveals continuous heterogeneity within each state, including a continuous tilt of FAF1, and describes a continuous motion linking the four states (Rebuttal Figure 1). This important information is currently confined to the rebuttal. I ask that a flexibility or continuous-heterogeneity analysis be included in the manuscript itself. Since the authors report that variability analysis on the full dataset was dominated by the large motor motions and did not converge, a deep generative approach such as cryoDRGN would be well suited to capturing the combined large-scale and local heterogeneity across the whole particle set. At minimum, some analysis of the continuum should appear in the main text so that the discrete states are framed as representative points along a continuous trajectory.*

We thank the reviewer for raising this important point and appreciate the opportunity to further clarify our interpretation of the data.

We acknowledge that our 3D variability analysis, as presented in the previous rebuttal, reveals continuous heterogeneity within each state, but as we previously discussed, this continuous heterogeneity is local and restricted mostly to the FAF1 region. We want to emphasize that our 3D classification of the dataset is highly reproducible, resulting in four distinct states. These states are not solely defined by the p97 seam position but also exhibit unique conformational features in the associated cis-side components, most notably in Npl4. The four different states are accompanied by clear and reproducible changes in the Npl4 and p97 conformation, supported by the corresponding density (resolution of 5 Å, 3.15 Å, 4.5 Å, and 5.8 Å for Npl4 in States I, II, III, and IV, respectively). These conformational differences contribute to the structural distinctions observed between the states, which we describe in detail in the manuscript.

We consider these states to represent distinct, populated conformational ensembles of the complex. Within these states, we acknowledge that more flexible components, such as FAF1 and the poly-ubiquitinated substrate, display conformational variability. This variability is evident due to the lower local resolution achieved for these components. Our 3D variability analysis supports a motion around the consensus position of FAF1 in State II, III and IV, which further explains the lower local resolution for these components (the 3D VA analysis was not successful for State I).

To address the reviewer's suggestion, we have expanded the discussion in the revised manuscript to emphasize that the identified states represent distinct conformational ensembles while also capturing the intrinsic flexibility of FAF1. Furthermore, we have incorporated and extended the analysis presented in previous Rebuttal Figure 1 into the revised manuscript as Supplementary Figure 17 (see below). Together with revisions of the main text, this provides a more comprehensive view of both the distinct states and the continuous heterogeneity within each state. We believe this approach provides a balanced and accurate representation of the data, acknowledging both the distinct states and the continuous heterogeneity within each state. We hope this clarification addresses the reviewer's concerns.

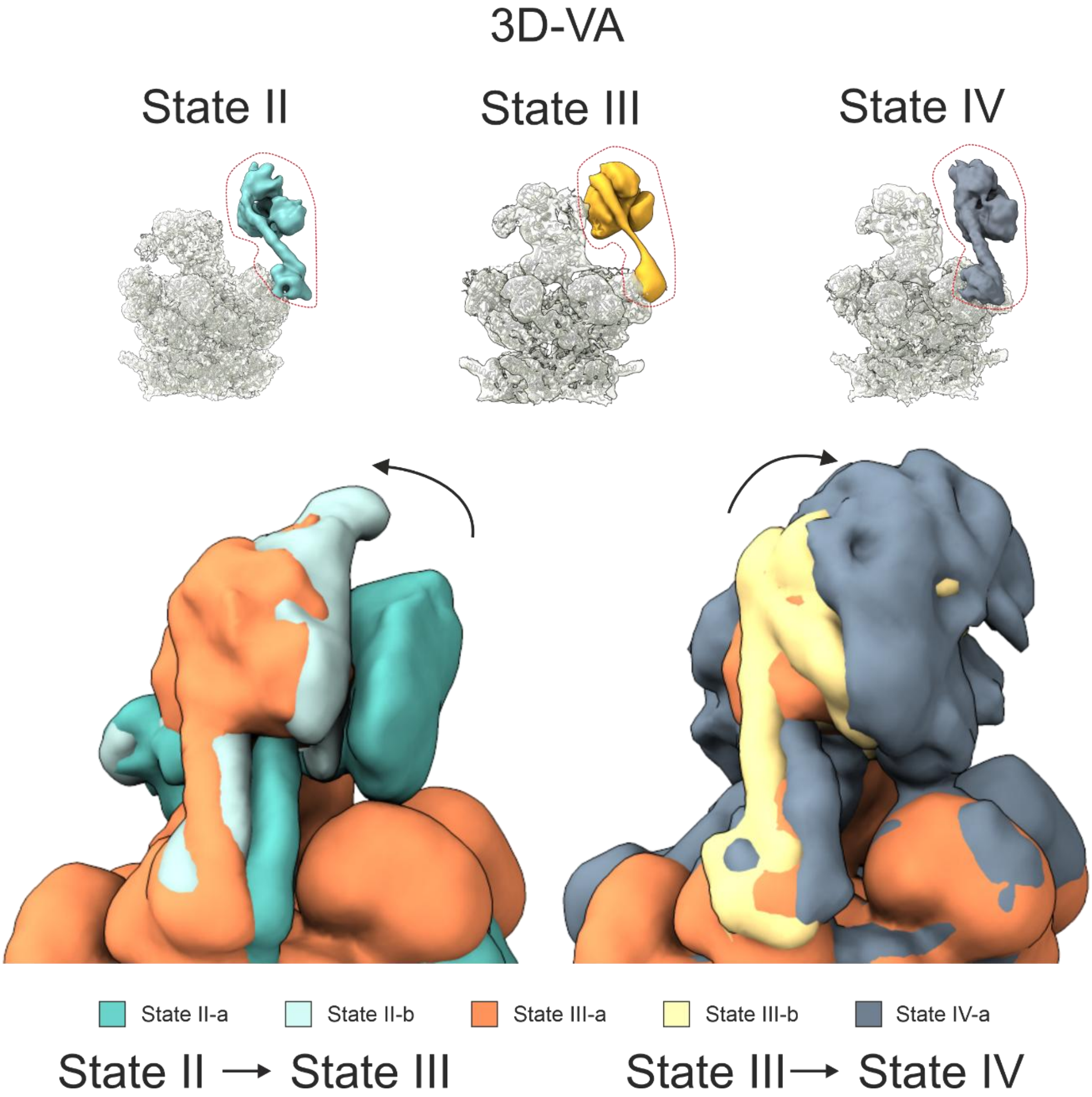

Supplementary Figure 17: *3D Variability (3D-VA) analysis of state II, III and IV focusing on FAF1, UT3 and proximal ubiquitins. The analysis reveals a continuous conformational trajectory involving FAF1, the Ufd1-UT3 domain, and the proximal ubiquitins. In particular, FAF1 undergoes a continuous tilting motion across the different states, supporting a continuous local conformational trajectory, while Npl4 and p97 retain distinct state-specific conformations. We show the two extreme states of 3D VA of the dataset of State-II (State-II-a; teal, State-II-b; cyan). We superposed them with the first extreme frame of State-III. Note that the two substates of State-II agree with a continuous movement (e.g. outward tilt of FAF1) towards State-III, supporting the proposing sequential model. Similarly, two extreme frames of 3D VA of State-III (State-III-a; orange, State-III-b; yellow) and first extreme frame of State-IV (State-IV-a; gray) are shown (e.g. inward tilt of FAF1).*

3. *Side chains should not be modeled or interpreted in the low-resolution regions. At the resolutions reported for Npl4 (4.5 to 5.8 A) and for FAF1 and the associated ubiquitins (8.6 to 12 A), side chains are not resolved and reliable main-chain register is not achievable. At least in the maps I have seen I dont think ubiquitin can be unambiguously placed. Supplementary Figure 11, which shows detailed interactions of the distal ubiquitins with the Npl4 tower and colours the surface by hydrophobicity, implies a level of side-chain detail the density does not support. I ask that these regions be shown as rigid-body-docked or backbone-level models only, without interaction claims that depend on specific side-chain placements. I am not disputing the FAF1 C-helix to UT3 interface itself, which is supported by the authors' biochemistry and by independent recent work; my point is only that side-chain detail should not be read out of this particular density.*

We thank the reviewer for this important comment regarding the modeling of low-resolution regions. We fully agree that side-chain interpretation must be supported by the resolution of the density.

We would like to clarify that in Figures 1, 2, and 3, as well as in Supplementary Figure 11, we exclusively present State II, which provided the highest resolution for the local refinements of FAF1 and the ubiquitins. In this state, the placement of the ubiquitins is consistent with the density and aligns well with AlphaFold predictions (for proximal ubiquitins) and previous structural studies for distal ubiquitins (doi: 10.1073/pnas.221370312). We have carefully reviewed the manuscript and ensure that our interpretations remain conservative and are fully supported by the data.

However, we agree with the reviewer regarding the representation in Supplementary Figure 11. We appreciate the reviewer pointing out that the display of side-chains in this figure implied a level of detail not supported by the resolution. To address this, we have updated Supplementary Figure 11 to show the ubiquitins as rigid-body docked models only (see below). Npl4, which was refined to 3.15 Å in State II, continues to be represented as a surface colored by hydrophobicity, as this is appropriate for the achieved resolution. Furthermore, we now display these docked models alongside the corresponding cryo-EM density for State II to provide a transparent illustration of the fitting quality.

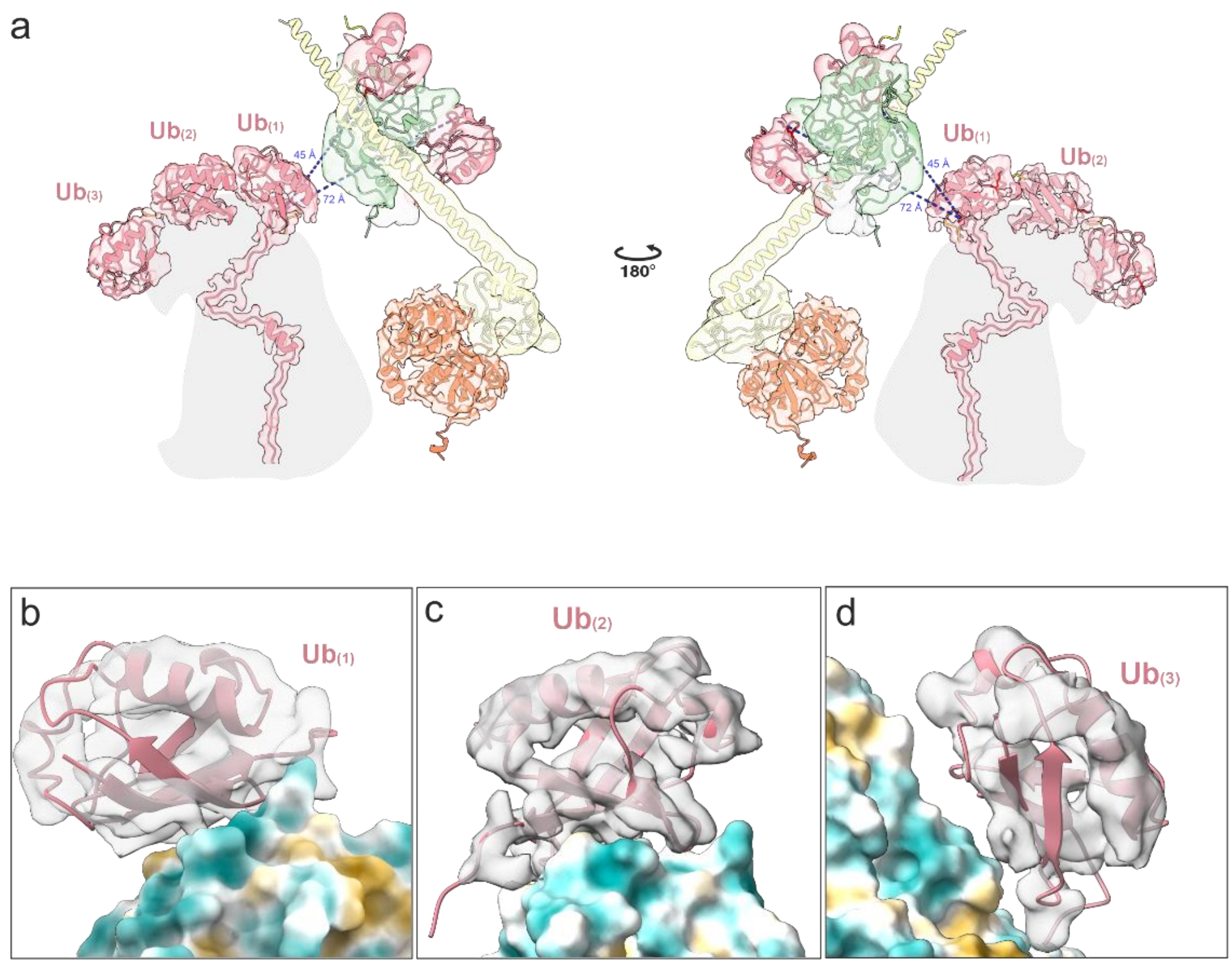

Revised Supplementary Figure 11: *Distal ubiquitins capping the Npl4 tower. a) Side view of the cryoEM volume and fitted models of stat II with Npl4 and p97 removed (except the NTD interacting with FAF1) (see Figure 3a). Dashed lines indicate the distance of the C-term of the initiator ubiquitin to the branchpoints of proximal to the substrate ubiquitins Ub(-1) and Ub(x) bound to FAF1 and UT3. b-d) View of the distal ubiquitins Ub(1) (b), Ub(2) (c) and Ub(3) (d) on the top of Npl4. Ubiquitins are fitted in their corresponding density colored in gray. Npl4 is represented as surface and colored by hydrophobicity.*

4. *The ubiquitin densities show features consistent with mask artefacts, with sharp boundaries suggesting density has been carved by the mask rather than reflecting its true extent. I credit the authors for already omitting ubiquitin moieties that lacked density (Supplementary Figure 8). I ask them to extend the same transparency to the retained ubiquitins by showing the unmasked, unsharpened maps in these regions, so the reader can judge which density is genuine.*

We thank the reviewer for this comment and for the suggestion to provide unmasked maps to ensure full transparency. We would like to clarify the following points:

- The ubiquitin densities presented in Figures 1, 2, 3, and Supplementary Figure 11 exclusively show State II, which provided the highest resolution and the most defined density for the ubiquitin-associated regions. The density in these figures is highly consistent with the rigid-body fitting of AlphaFold models (proximal) and available structures (distal), supporting the orientation of the ubiquitins and their termini (see also Revised Supplementary Figure 11).
- As noted by the reviewer, ubiquitin moieties in AlphaFold models lacking interpretable density were omitted from the final models (as shown in Supplementary Figure 8) to avoid over-interpretation.
- For States I, III, and IV, the local refinement of the FAF1/UT3 and proximal ubiquitin regions was initially more challenging. To demonstrate the overall inward-outward tilt of FAF1 and its associated components (Figure 4), we used the resulting State II model as a rigid-body reference to fit the respective densities of the other states, as we describe in the main manuscript. This approach was used solely to illustrate the global conformational changes and not to make specific side-chain or interaction claims.
- We have since further improved the local refinement for the FAF1/UT3 and proximal ubiquitin regions of State IV (updated Supplementary Figure 13; see below). This refined map confirms that the rigid-body model from State II agrees well with the improved density of State IV upon tilting of the FAF1 α-helix.
- Following the reviewer's suggestion, we have included new views of the retained ubiquitin densities using unmasked, unsharpened, and locally filtered maps in the new Supplementary Figure 13. These representations allow for a direct evaluation of the genuine density supporting the AlphaFold models for the locally refined maps of State II, the updated map of State IV, and the consensus maps of all states.

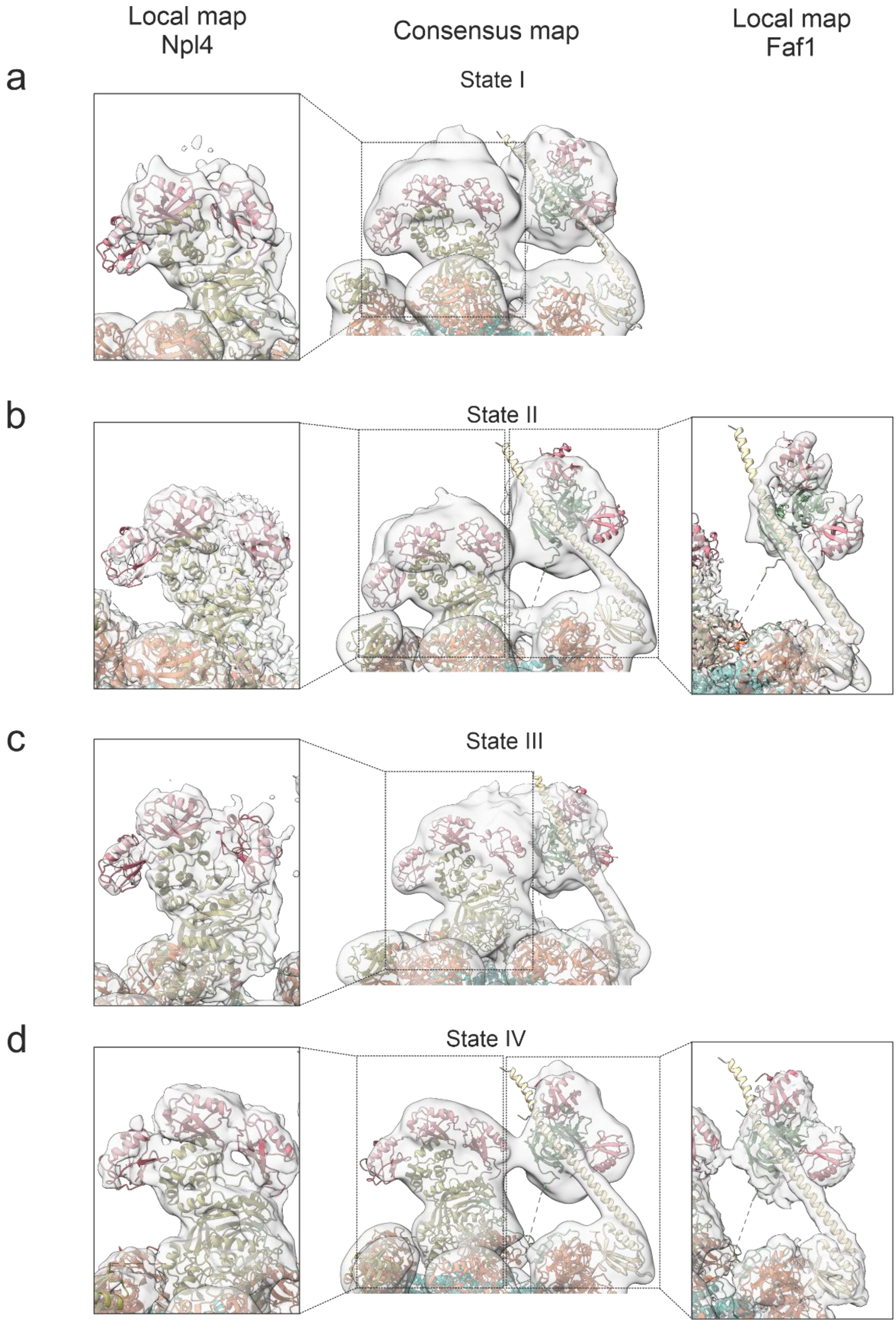

Supplementary Figure 13*: Consensus and locally refined maps of the Npl4 and FAF1/UT3–proximal ubiquitin regions across States I–IV. The four states are shown in (a–d), with State I in (a), State II in (b), State III in (c), and State IV in (d). Unmasked filtered consensus maps are shown in the middle column with the corresponding fitted models. Where available, locally refined maps are shown in the left and right columns. The left column shows the locally refined Npl4 region with the corresponding fitted model (for states I, II, III and IV), while the right column shows the locally refined FAF1/UT3–proximal ubiquitin region with the corresponding fitted models (for states II and IV).*

5. *It should be stated which map is displayed in each figure. I found it difficult to get similar clear representations of fits with the composite maps provided and to assess the quality of the fits and it is not always clear which map underlies each panel. Please state explicitly, for every panel showing density, which map is shown (consensus, locally refined, or composite, and which state). Please at least for me include with the contour level and EMDB accession; a short table mapping figures to maps would be ideal.*

We thank the reviewer for this recommendation, as it significantly improves the transparency and accessibility of our data. We would like to clarify that the composite maps are not used in any of the figures in the manuscript; we regret any confusion this may have caused.

To ensure full clarity, we have implemented the following:

1. We have updated the legends for all figures to explicitly state which map is shown in each panel (e.g., locally refined map of State II) and have included the corresponding EMDB accession codes.
2. As suggested, we have included a summary table (see Supplementary Table 4) that maps every figure panel to its corresponding map, state, and EMDB entry.

Please find the maps and models in the following link:

Link: https://uni-muenster.sciebo.de/s/yXnocGQpSemgtkJ

Password: FAF1paper

We thank Reviewer 3 for their additional comments on our work. Below, we provide a point-by-point response to each comment and describe the revisions made to the manuscript in light of their suggestions.

<u>Reviewer 3</u>

I thank the authors for a constructive revision. The new 3D variability analysis, the new Supplementary Figure 13, the improved local refinement of the State IV FAF1/UT3 region, the revised Supplementary Figure 11, and the new Supplementary Table 4 all move the manuscript in the right direction, and my concerns about the biological conclusions are resolved. The central claims, namely, a single FAF1 per p97 hexamer, docking of the UBX domain onto the same NTD that binds the Ufd1 SHP box, engagement of proximal ubiquitins by the FAF1 C-helix and the Ufd1 UT3 domain, and four seam-defined conformational states, are well supported by the data as now presented.

However, some of the changes described in the response letter are either not evident in the submitted files or have not been implemented consistently throughout. The revision has also introduced a small number of internal inconsistencies. I would ask the authors to address the following points before acceptance.

Overall, the manuscript has improved substantially, and I consider the major biological and mechanistic conclusions adequately supported. The remaining points concern consistency of presentation, transparency of map provenance and validation, and ensuring that interpretations are stated at a level appropriate to the local resolution. I do not think these issues require substantial new experiments or a fundamentally different analysis, but they should be resolved before publication.

We are grateful to the reviewer for their continued guidance and for acknowledging the substantial improvements to the manuscript. We have carefully addressed the remaining points and we trust that the detailed responses provided below fully satisfy the reviewer's requirements.

1. *The response states that the Discussion was expanded to emphasize that the identified states represent distinct conformational ensembles while capturing the intrinsic flexibility of FAF1. I could not identify this specific clarification in the submitted Discussion; in particular, the term "conformational ensemble" does not occur in the manuscript. The new explicit treatment of continuous variability appears instead as a paragraph at the end of the Results. Please either incorporate the clarification promised in the response into the Discussion or indicate explicitly where this point has been addressed.*

We have now revised the Discussion to ensure the promised clarification is explicitly stated. We have introduced the term 'conformational ensemble' and expanded the text to better explain the link between the identified states and the continuous flexibility of the *cis*-side components.

2. *My earlier comment about modelling and interpreting side chains in lower-resolution regions has been addressed for Figures 1–3 and for Supplementary Figure 11, and I am satisfied with the revised Supplementary Figure 11. The same issue, however, remains in Supplementary Figure 16, which was not correspondingly modified. That figure annotates individual residues on surfaces coloured by hydrophobicity, in states for which Supplementary Table 2 reports Npl4 local resolutions of 5.0, 4.48 and 5.82 Å, respectively. Please either present these interfaces at backbone/rigid-body level without residue-specific annotation, or state the local resolution in the legend together with an explicit statement that the side-chain positions are not determined by the density and are shown only to indicate the region involved. There is also a minor*

labelling inconsistency: the heading of Supplementary Figure 16 refers to States I, II and IV, whereas the panels shown are States I, III and IV.

We have revised the legend of Supplementary Figure 16 to report the local resolution of Npl4 for each state and to explicitly clarify that, at these resolutions, the density does not support unambiguous assignment of side-chain positions, which are shown only to indicate the approximate regions involved. We have also corrected the heading to accurately list States I, III, and IV.

3. *The improved State IV FAF1/UT3 local refinement added in response to my concern about possible mask artefacts reports a resolution of approximately 6.8 Å in Supplementary Figure 5. However, its FSC panel does not show the same full set of curves as the other refinements. Instead, the displayed curves are labelled "Auto-tight Mask," "Auto-tight Mask, corr" and "Input Mask," whereas the other panels show the No Mask, Loose, Tight and Corrected curves. In addition, the corrected curve appears to drop extremely abruptly over a very narrow resolution range. Without the corresponding unmasked and loose-mask FSC curves, it is difficult to exclude an effect of an overly restrictive mask, which is particularly relevant because this refinement was introduced in response to my concern about masking. Please show the full set of FSC curves for this refinement, consistent with the other panels.*

We apologize for the inconsistency in the FSC panels. The State IV FAF1/UT3 local refinement was performed using a more recent version of CryoSPARC, which employs different default FSC labels and output formats than the versions used for the other refinements.

To address the reviewer's concern and ensure consistency, we have updated Supplementary Figure 5 to include the full set of available FSC curves from the recent refinement. We have also updated the figure legend to clarify the labelling differences between the versions. These data, specifically the inclusion of the unmasked and loose-mask curves, confirm that the mask is not overly restrictive and that the reported resolution estimate is robust.

4. *I appreciate the addition of Supplementary Figure 13. However, the unmasked consensus maps there are shown for the Npl4 and FAF1/UT3 region as a whole at a single contour, so the individual distal ubiquitins Ub(1), Ub(2) and Ub(3) still cannot readily be assessed against the unmasked density. The per-ubiquitin panels in Supplementary Figure 11b–d appear to show locally refined density, but the legend does not specify whether the displayed maps are unmasked. In panels c and d, portions of the modelled β-sheet also appear to extend outside the displayed density envelope.*

The densities presented in Supplementary Figure 11 were obtained via segmentation of the map based on the fitted model. We wish to assure the reviewer that this process was performed rigorously to avoid introducing artifacts or bias, and specifically we ensure that no genuine unassigned densities were removed. The masking was applied solely to show the density of individual Ubs without any surrounding noise and other proteins/domains; therefore, there is no difference between the masked and unmasked densities that would influence the appearance, fitting or orientation of the Ubiquitins.

To address the reviewer's concern, we provide a direct comparison in Rebuttal Figure 1, where the locally refined masked maps (upper row, as seen in Supp. Fig. 11) are shown alongside the unmasked density prior to segmentation (lower row). This comparison demonstrates that the densities for the distal ubiquitins are genuine.

Regarding the visibility of the density, Ub(1), Ub(2), and Ub(3) exhibit varying levels of local resolution. Consequently, in Supplementary Figure 11, we have applied different contour thresholds for each ubiquitin to best represent their respective densities; this is explicitly stated in the revised figure legend. We acknowledge that some portions of the modeled β -sheets for Ub(1) and Ub(3) extend beyond the visible density envelope. This is a result of the lower local resolution for these two moieties. However, the rigid-body fitting remains unambiguous, and the final model is fully consistent with the established structural models of distal ubiquitins reported in [DOI: 10.1126/science.aax1033] and [DOI: 10.1073/pnas.2213703120]

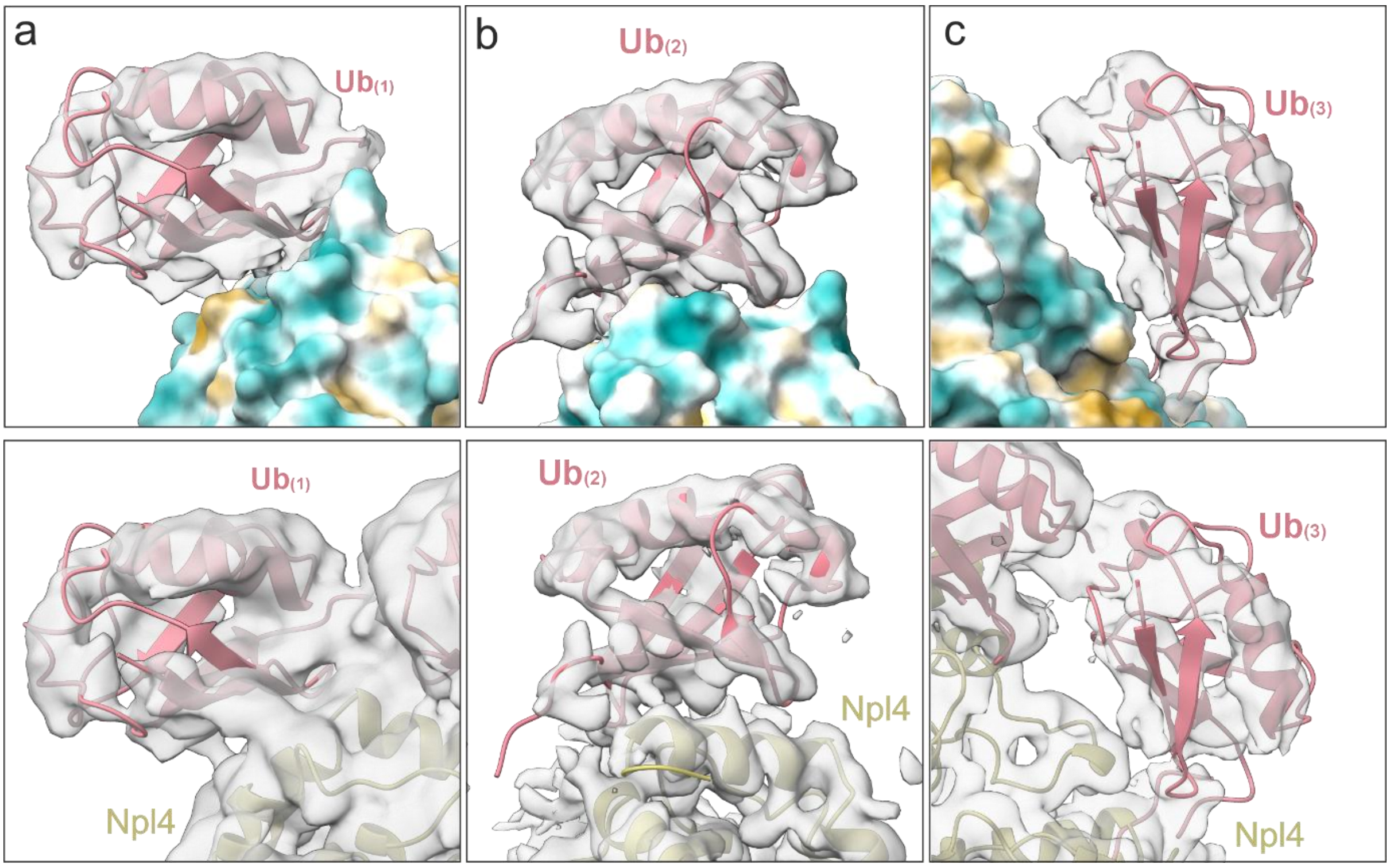

Rebuttal Figure: *Comparison of segmented and unmasked cryo-EM densities for the distal ubiquitins. Upper panels show the locally refined density maps after model-based segmentation, as presented in Supplementary Figure 11, whereas lower panels show the corresponding unmasked densities prior to segmentation. Npl4 is shown as a surface representation, coloured according to hydrophobicity in the upper panels and dark yellow ribbon in the lower panels. The contours for the lower panel are 9.24σ, 9.3σ and 7.07σ, respectively.*

5. *The new 3D variability analysis is a useful addition, but it was performed separately within each already-classified state. Residual heterogeneity within an already classified subset can include conformations extending toward neighbouring classes, so the observation that the extreme frames of State II point toward State III is consistent with, but does not by itself establish, a sequential trajectory. I therefore ask the authors to soften "supporting sequential state transition" in the Results and "supporting the proposing sequential model" in the legend of Supplementary Figure 17 to wording such as "consistent with the proposed sequential transition/model," and to add one sentence acknowledging this limitation. Generally, cryodrgn analysis should do a better job here or at least not run variability on pooled particles and not individual states.*

   We have revised the wording in the Results and the legend of Supplementary Figure 17 to state that the observations are 'consistent with' rather than 'supporting' the sequential transition/model. We have also added a sentence in the legend acknowledging that 3DVA performed within individual states captures residual heterogeneity and may not

independently establish a sequential trajectory. However, we wish to clarify that our proposed sequential model is not based solely on these 3DVA trajectories, but is derived from the distinct structural relationships between the four classified states, integrated with biochemical data and the known progression of the p97 ATPase cycle.

6. *Contour levels are currently given only in the legend of Figure 1. Figures 2, 3 and 4 and the supplementary figures showing cryo-EM density do not provide them, although my previous request was for contour level together with the corresponding accession code. Supplementary Table 4 is a helpful addition, but it is organized per figure rather than per panel, despite its title, and does not cover the supplementary density figures, including at least Supplementary Figures 5, 11, 12, 13, 14 and 15.*

   The table also disagrees with the main-figure legends. The legend of Figure 2 lists EMD-58590, EMD-58591 and EMD-58592, whereas Supplementary Table 4 lists only EMD-58590 and EMD-58592. Similarly, the legend of Figure 3 lists EMD-58590, EMD-58591 and EMD-58592, whereas the table lists only EMD-58592. The newly deposited State IV FAF1 refinement, EMD-59179, is also absent from Supplementary Table 4. Please complete and reconcile the table and provide contour levels for the density panels.

We have revised Supplementary Table 4 to ensure that information is provided for each individual panel, and updated the title to reflect this change. The table has been expanded to provide a comprehensive list of contour levels and EMDB accession codes for all maps in the main and supplementary figures, including the previously omitted supplementary density figures and the State IV FAF1 refinement (EMD-59179). Furthermore, we have reconciled the discrepancies between the figure legends and the table to ensure consistency across the manuscript and have added the required contour levels to the relevant figure legends.

7. *Figure 1 displays three separate maps at independently chosen contour thresholds: 15.7σ for FAF1, 6.2σ for the UN tower and 7.8σ for p97. I accept that these are not composite maps and that the poorer local resolution of the FAF1/ubiquitin region is visually apparent. Nevertheless, independently chosen thresholds mean that relative density strength or apparent occupancy cannot be compared directly between the three displayed components. One sentence in the legend stating that the components are contoured independently and that apparent occupancy or relative density strength should therefore not be compared between them would remove any ambiguity.*

We have added a sentence to the Figure 1 legend clarifying that the FAF1, UN tower, and p97 components are contoured independently and that their apparent occupancy or relative density strength should therefore not be compared directly.
